# SMURF: soft-segmentation for single-cell reconstruction and topological analysis of spatial transcriptomic data

**DOI:** 10.1038/s41467-026-74464-4

**Published:** 2026-06-26

**Authors:** Juanru Guo, Simona Sarafinovska, Ryan A. Hagenson, Mark C. Valentine, David Y. Chen, William H. McCoy IV, Joseph D. Dougherty, Robi D. Mitra, Brian D. Muegge

**Affiliations:** 1 Department of Genetics, Washington University in St. Louis School of Medicine, Saint Louis, MO USA; 2 Edison Family Center for Genome Sciences and Systems Biology, Washington University in St. Louis School of Medicine, Saint Louis, MO USA; 3 Department of Psychiatry, Washington University in St. Louis School of Medicine, Saint Louis, MO USA; 4 Department of Medicine, Washington University in St. Louis School of Medicine, Saint Louis, MO USA; 5 Department of Obstetrics and Gynecology, Washington University in St. Louis School of Medicine, Saint Louis, MO USA; 6 Division of Dermatology, Washington University in St. Louis School of Medicine, Saint Louis, MO USA; 7 Intellectual and Developmental Disabilities Research Center, Washington University in St. Louis School of Medicine, Saint Louis, MO USA; 8 McDonnell Genome Institute, Washington University in St. Louis School of Medicine, Saint Louis, MO USA; 9 Department of Medicine, VA Medical Center, Saint Louis, MO USA

**Keywords:** Systems biology, Computational biology and bioinformatics, Developmental biology, Genomics

## Abstract

High-resolution spatial transcriptomics requires computational methods to accurately assign transcripts to individual cells. We present SMURF (Segmentation and Manifold UnRolling Framework), a cross-platform soft-segmentation algorithm that uses deep learning to map mRNAs from capture spots to nearby nuclei. SMURF also unrolls complex tissue architectures by projecting cells onto Cartesian coordinates, enabling analyses of cell-type organization and gene expression gradients. We show that SMURF assigns mRNAs to single cells more accurately than existing approaches and robustly unrolls complex tissues to reveal zonated transcriptional programs and cell-type organization across multiple tissues and spatial transcriptomic technologies. To showcase the biological insights enabled by SMURF, we segment over 400,000 cells from the mouse ileum using Visium HD data. We identify zonated gene expression programs along the maturing intestinal villus and the transcription factors that regulate them. Importantly, we show that gene expression gradients along the proximal-distal axis of the intestine accumulate in the upper villus and that upper villus gene expression is reprogrammed by environmental signals in the lumen, suggesting that environmental inputs are major determinants of regional transcriptional identity. Together, these results establish SMURF as a powerful framework for analyzing gene expression of cells within native tissue contexts.

## Abstract (web summary)

SMURF uses deep learning and topological analysis to assign mRNAs to single cells in spatial transcriptomics data, recovering rare cell types and uncovering environmentally regulated gene expression programs in the mouse intestine.

## Introduction

Biology is poised to address a fundamental challenge: describing the molecular features of all cell types in the body at single-cell resolution. But a full understanding of how cells function cannot be achieved without considering how they exist within their microenvironments, as cells of the same subtype may have substantially different properties depending on their niche. Spatial transcriptomics (ST) addresses this challenge by allowing researchers to measure cellular gene expression while preserving spatial context.

Earlier capture-based ST platforms used relatively coarse, multi-cell capture spots, but emerging high-resolution methods are now approaching single-cell or even subcellular granularity. High-resolution capture-based ST platforms (e.g., Visium HD, Stereo-seq, Slide-seq) dramatically increase spatial granularity but also introduce analytical challenges^1–7^. First, the capture elements (spots/pixels/beads/tiles) are not intrinsically co-registered to cell boundaries; many lie in extracellular space and therefore capture few or no transcripts. Second, transcripts from a single cell can be dispersed across several nearby capture elements, and conversely, individual capture elements near cell edges often contain transcripts from multiple neighboring cells. Most existing high-resolution segmentation methods implicitly assume one cell contributes mRNA to one capture element^8,9^, an assumption that is frequently violated at these resolutions. Thus, there is a need for computational approaches that accurately segment and deconvolve mRNA in capture spots and assign them to single cells.

Another obstacle to fully harnessing the power of ST is that most tissues exhibit complex three-dimensional architectures. Many organs—including the intestine, retina, and cerebral cortex—are composed of repeating or curved substructures that can be conceptualized as biological manifolds^10,11^. Within these manifolds, cells are arranged along defined spatial axes that reflect essential biological gradients, such as differentiation, signaling, and metabolic specialization^12–14^. However, current analytical tools are poorly suited to interrogate transcriptional organization along these non-Cartesian geometries. This challenge is compounded by tissue deformation and distortion during sample preparation, which obscures native spatial relationships. A computational framework capable of unrolling such complex tissue structures onto standard Cartesian coordinates would therefore enable quantitative analyses of regional gene-expression programs across diverse tissue substructures.

To address these challenges, we developed the Segmentation and Manifold UnRolling Framework (SMURF). SMURF introduces the concept of soft segmentation, in which mRNA counts within a capture spot can be assigned wholly to a single cell or fractionally distributed among multiple nearby cells in a manner that optimizes the cosine similarity of each cell to its corresponding gene-expression cluster. This approach increases the number of mRNAs assigned to individual cells and improves both clustering precision and transcript recovery, yielding state-of-the-art performance across diverse datasets. Beyond segmentation, SMURF provides a platform-independent framework to computationally unroll complex tissue structures using topological theory, thereby revealing transcriptional organization along continuous biological axes that are otherwise hidden. Applying SMURF to sequencing-based datasets (e.g., Visium HD and Stereo-seq) and imaging-based datasets (e.g., CosMx, MERFISH, and Xenium Prime), we recovered fine-grained spatial structure and cell-type organization in brain, retina, skin, and intestinal tissues.

To showcase the biological insights enabled by SMURF, we segmented and analyzed more than 400,000 cells from the healthy mouse ileum (distal small intestine) using Visium HD data. The intestine presents a unique and important place to study spatial organization because millions of repeating crypt-villus units across the length of the intestine are exposed to regionally diverse dietary components, microbes, and host signals. We found SMURF captured all major intestinal cell types, including rare cell types that were not identified with conventional methods. Using SMURF’s manifold unrolling capability, we identified zonated transcriptional programs along both the crypt-to-villus and proximal-to-distal axes, and we present a community resource of enterocyte maturation markers along the ileal villus that has single-cell resolution. We also identified transcription factor networks that regulate these programs.

Finally, we used SMURF to investigate a fundamental question in the development of the small intestine: are proximal-to-distal gradients in gene expression predetermined, or are they established in response to signals in the luminal environment? We found that gene programs that vary along the proximal-to-distal axis are almost exclusively expressed in the upper villus, leading us to hypothesize that signaling molecules in the lumen drive proximal-to-distal gradients in gene expression. We verified this hypothesis using a single-cell dataset where a segment of the distal small intestine was surgically moved to a proximal location in an adult mouse. Together, our analyses show that SMURF robustly segments high-resolution spatial data, sensitively detects rare cell types, and computationally unrolls cells onto Cartesian coordinates to enable manifold-level analysis of tissue architecture. By integrating manifold learning with soft segmentation in a platform-independent framework, SMURF establishes a general computational foundation for analyzing transcriptional organization in complex tissue geometries.

## Results

### Soft-segmentation for spatial transcriptomics

The capture elements, or spots, of high-resolution ST methods are not aligned with cellular boundaries. Some spots collect mRNA from multiple cells, and conversely, most cells have transcripts that are distributed across multiple spots. This makes it difficult to reliably assign transcripts to individual cells. SMURF overcomes this limitation using a three-step soft segmentation approach.

**Fig. 1:**
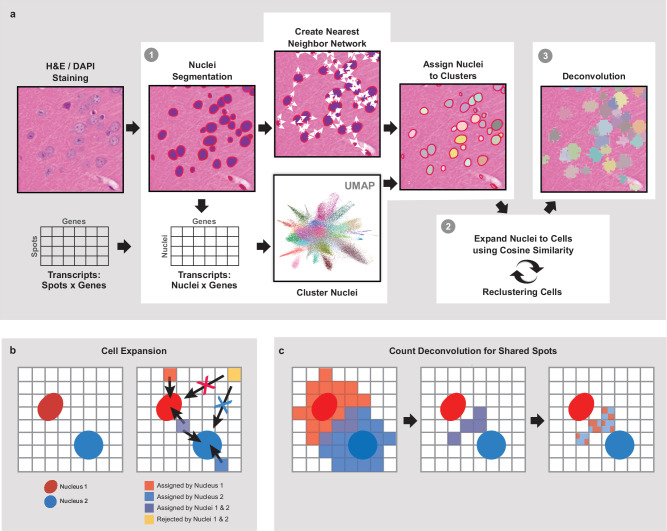
SMURF uses soft segmentation to reconstruct single-cell transcriptomes. **a** Schematic of SMURF's soft-segmentation algorithm. SMURF segments nuclei from the image data, assigns transcripts to nuclei from overlapping capture spots and clusters the resulting nuclei-by-genes matrix (Step 1). The algorithm then iteratively expands cell boundaries to assign transcripts from capture spots that do not overlap nuclei to cells (Step 2). Finally, SMURF leverages deep learning to deconvolve spots assigned to multiple cells during the expansion step (Step 3). **b** Example of capture spot assignment to cells. Capture spots can be assigned to zero, one or multiple nuclei during cell expansion (Step 2), depending on their proximity and expression similarity. **c** Count deconvolution for shared spots. After the cell expansion step, capture spots may be shared between multiple cells (purple squares in the first two panels). Transcripts from shared capture spots are divided among cells using a quadratic programming or deep learning algorithm (see Methods).

In the first step, SMURF identifies nuclei from H&E or DAPI/IF images using established segmentation methods^15–18^. It then constructs a gene expression matrix by assigning mRNA counts from the capture spots that directly overlie each nucleus. Nuclei are then clustered by expression similarity using Scanpy^19–21^. Simultaneously, SMURF builds a nearest-neighbor network based on nuclear positions that is used in the second step to assign surrounding capture spots to nearby nuclei (Fig. 1a).

Because nuclei occupy only a small fraction of total cell volume, the initial single-nucleus gene expression matrix is sparse, with low UMI counts^22^. Thus, the second step of soft-segmentation expands the nucleus-anchored gene expression by incrementally assigning nearby spots to nuclei through concentric expansion. Nearby spots are added only if they increase the cosine similarity between the expression profiles of the nucleus and its cluster. During concentric expansion, new spots can be assigned asymmetrically, allowing SMURF to recover non-circular and elongated cell morphologies when supported by gene expression similarity and proximity. This process is repeated iteratively: nucleus assignments and cluster identities are alternately updated until gene expression clustering stabilizes according to normalized mutual information (NMI)^23^. SMURF allows flexible assignment at this step, as a spot can be linked to one nucleus, several nuclei, or none (Fig. 1b).

The third and final step of SMURF’s soft-segmentation is count deconvolution for spots shared by multiple cells. SMURF deconvolves individual mRNA counts from capture spots assigned to multiple cells, using a combination of quadratic programming and deep learning (Fig. 1c, Methods)^24,25^.

### SMURF improves single-cell reconstruction of high-resolution ST data

**Fig. 2:**
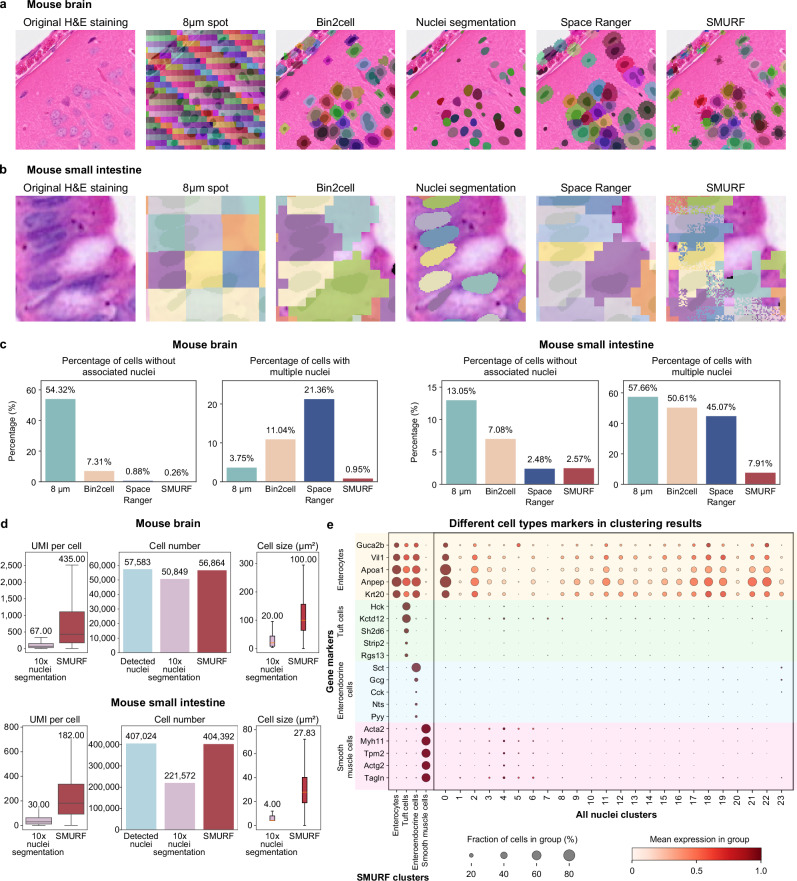
SMURF accurately assigns transcripts to cells in mouse brain and mouse small intestine data. **a**,**b** Representative images illustrating how SMURF and other ST segmentation methods aggregate Visium HD capture spots to define cells in the mouse brain (top row) and mouse small intestine (bottom row). Capture spots are shaded according to their assigned cell. SMURF uniquely manages to both assign only one nucleus per cell and analyze captured spots under cell bodies. **c** Percentage of cells without an associated nucleus or containing multiple nuclei in the mouse brain and mouse small intestine using different ST segmentation strategies. **d** Comparison of UMIs per cell, detected cell numbers, and cell sizes obtained using SMURF or 10x nuclei-based segmentation in one mouse brain tissue section and one mouse small intestine tissue section. Left, UMIs per cell; middle, number of detected nuclei or segmented cells; right, cell sizes. Numbers above box plots indicate median values, whereas numbers above bars indicate the total number of detected nuclei or segmented cells. Box plots show the median, interquartile range, and 1.5 × interquartile range whiskers. No biological replicate comparison was performed. **e**, Cell type detection in mouse small intestine using clusters generated by SMURF or nuclei segmentation. Expression of marker genes for four intestinal cell types is plotted across clusters. SMURF, but not nuclei segmentation, has clusters that clearly correspond to each cell type. Abbreviation: UMI unique molecular identifier.

We benchmarked SMURF’s soft segmentation algorithm using Visium HD data from the mouse brain and distal small intestine (ileum)^26,27^. We compared SMURF to seven commonly used ST methods, evaluating runtime and performance (See Supplementary Note, Supplementary Table 1, Supplementary Figs. 1 and 2). Here, we focus on comparison with the four algorithms specifically designed to analyze Visium HD data (Fig. 2a, b). The first of these is the 8 μm method, which was 10x Genomics’ original default analysis algorithm, which aggregates 16 2-μm-spots into 8 × 8 μm bins to increase UMI counts for clustering and differential expression. It does not consider nuclear boundaries, often assigning transcripts to bins that lack nuclei, only partially cover a nucleus, or span multiple nuclei (Fig. 2a, b, 2nd column). The second method is Bin2cell, a recently described approach for single-cell segmentation which performs better than the 8 μm method in the mouse brain but often merges multiple cells or misses nuclei entirely in the densely packed small intestine (Fig. 2b, 3rd column)^9^. The third method we benchmarked is nuclei segmentation, which only uses spots directly overlapping nuclei; while this strategy prevents the assignment of transcripts to cells lacking nuclei or to cells with multiple nuclei, this approach results in a sparse expression matrix, as it ignores many transcripts from the surrounding cytoplasm (Fig. 2a, b, 4th column). The fourth method is Space Ranger, which is the current default method for 10x Genomics Visium HD analysis. In our benchmark, Space Ranger’s segmentation algorithm assigned a relatively high number of UMIs per cell (Supplementary Fig. 1), but this came at the cost of merging a substantial number of neighboring cells (Fig. 2a, b, 5th column). In contrast to these other methods, SMURF reliably achieves single-cell resolution while accurately recovering cytoplasmic transcripts (Fig. 2a, b, last column).

We next quantified segmentation accuracy by measuring the percentage of cells lacking a nucleus and the percentage containing multiple nuclei for each method (Fig. 2c). SMURF was the most accurate in both brain and small intestine data. Only 0.26% of SMURF cells in the mouse brain lacked a nucleus, and only 0.95% contained multiple nuclei. This was significantly better than Bin2cell, the 8 μm method, or the default 10x method, Space Ranger. For example, 21.36% of the cells detected by Space Ranger in the mouse brain contained multiple nuclei, and this proportion rose to 45.07% in the small intestine (Fig. 2c). SMURF also outperformed these methods in the densely populated small intestine, with a substantially lower rate of multinucleated cells (7.91% versus 45–58% for all other methods). SMURF and Space Ranger had comparable rates of nuclei-less cells (2.57% versus 2.48%), greatly outperforming the 8 *μ*m method and bin2cell, which had rates of 13.05% and 7.08%, respectively, demonstrating that SMURF more accurately assigns transcripts to cells than existing methods (Fig. 2c, right panels). Compared to the nuclei segmentation approach, SMURF assigned 6-7 × more UMIs to each cell, and the average cell size was bigger (Fig. 2d). We also found that SMURF’s cell-level normalization corrects a severe count imbalance artifact observed in transcript-sparse regions when using the default 8 *μ*m method in Visium HD (Supplementary Note, Supplementary Fig. 3a). Reconstructed high-resolution spatial single-cell counts were also more accurately modeled by a negative binomial distribution than by Poisson-based alternatives, consistent with substantial overdispersion in these data (Supplementary Note, Supplementary Table 1, Supplementary Figs. 4 to 7). Together, these results demonstrate that SMURF accurately assigns Visium HD counts to single cells, with substantially more counts per cell than nuclei segmentation alone.

We next evaluated whether SMURF’s soft segmentation generalizes across high-resolution, capture-based spatial transcriptomic platforms by applying it to Stereo-seq mouse brain and Seq-Scope mouse liver datasets. In both, SMURF assigned substantially more UMIs per cell than nuclei-based segmentation (Supplementary Fig. 3c). To benchmark against another nuclei expansion approach, we compared SMURF soft segmentation to results obtained with the Spateo morphological expansion algorithm^2^. SMURF’s probabilistic soft-expansion produced higher correlations between nuclei masks and their corresponding expanded regions than morphological expansion (Supplementary Fig. 3d). Together, these results demonstrate that SMURF effectively works across capture-based spatial transcriptomic datasets and provides more accurate transcript assignment than conventional morphological methods.

### SMURF improves cell type identification and capture of rare cell types

We hypothesized that SMURF’s higher transcript assignment per cell would lead to more accurate cell-type identification and improved estimation of their relative abundances. To test this, we used cell-type proportions from a probe-based ST study of the mouse cortex as an external reference dataset^28^. Using Kullback-Leibler (KL) divergence^29^, we quantified how closely SMURF and nuclei segmentation (without expansion) reproduced the probe-based ST-derived proportions. SMURF showed substantially lower KL divergence (0.122 vs. 0.738), indicating a more accurate representation of cell-type abundances (Supplementary Fig. 3b).

Comparable ground-truth data for the small intestine are limited, so to benchmark SMURF’s ability to detect rare cell types, we clustered and annotated cells independently for the nuclei segmentation method and for SMURF (Fig. 2e). Both methods detected enterocytes, the dominant epithelial population (Fig. 2e). However, only SMURF reliably identified rarer epithelial cell types, such as tuft cells and enteroendocrine cells (EECs), as well as smooth muscle cells from the muscularis layer. These cell types were not captured by nuclei segmentation, potentially due to irregular nuclear morphology, low nuclear transcript abundance, or mRNA polarization, factors known to affect these cell populations. Similarly, Space Ranger cell assignments did not robustly recover these rare cell types (Supplementary Fig. 8). The calculated abundance of epithelial cell types using SMURF assignments was broadly consistent with previous scRNA-seq and ST intestinal surveys (Supplementary Table 3)^30–32^. SMURF detected the expected cell types from epithelial, stromal, and immune compartments (Supplementary Table 2).

Taken together, our results show that SMURF accurately assigns transcripts from both the nucleus and cytoplasm to individual cells. Compared to nuclei segmentation, SMURF recovers more transcripts per cell and improves the identification of known cell types in Visium HD data. SMURF’s increased count depth enhances statistical power for downstream analyses and enables more reliable detection of rare or morphologically irregular cell types.

### SMURF utilizes manifold learning to project ST data from complex tissue substructures onto Cartesian coordinates

Having assigned transcripts to individual cells, the next challenge is to reconstruct how these cells are spatially arranged within the intricate geometries of real tissues. Spatial relationships are critical for interpreting gene-expression patterns, but most tissues contain curved or layered substructures that do not align with standard Cartesian grids, hampering the analysis of cell organization or gene expression gradients in these substructures. To address this, we developed a manifold learning approach that projects cells from complex tissue shapes onto a coordinate system aligned with the structure of interest (Methods). Manifold learning is a form of topological analysis that characterizes complex structures by establishing local homeomorphisms between the neighborhoods of a surface and Euclidean space. SMURF uses cell-resolved transcriptomes to identify tissue structures (e.g., the cortical layers of the brain or the muscularis mucosae of the intestine) and defines a manifold along that structure. SMURF then unrolls this manifold, projecting the cells onto standard Cartesian axes. This functionality enables the quantitative analyses of cellular organization and regional specification within the coordinate system most interpretable for each tissue.

**Fig. 3:**
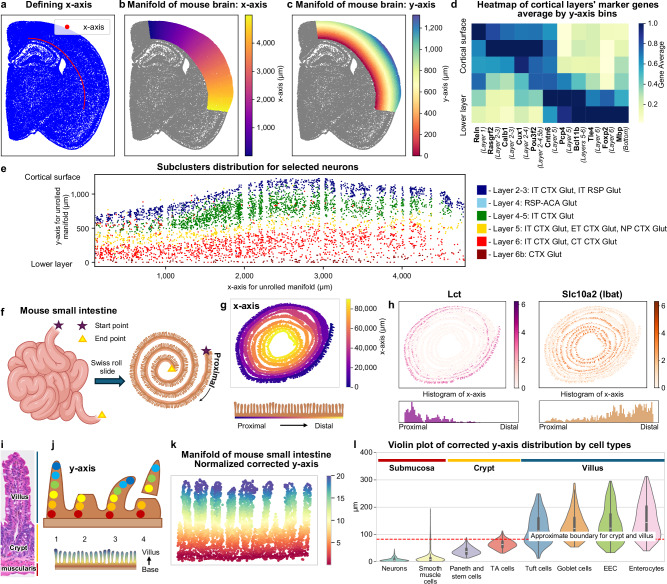
Manifold unrolling of Visium HD data in the cortex and small intestine. **a** Selection of the *x*-axis contour in the mouse cortex based on the positions of cells in the basement membrane cluster. **b** Overlay of cortical manifold onto selected mouse brain section, colored by *x*-axis (*μ*m). **c** Overlay of cortical manifold onto selected mouse brain section, colored by *y*-axis (*μ*m). **d** Heatmap of marker genes for cortical layers across different *y*-axis bins of the mouse brain manifold on the selected mouse brain section. **e** Distribution of cortical layer-specific neurons in the unrolled rectilinear coordinate system. A total of 5161 cells are shown. **f** Schematic illustration of the mouse small intestine and its Swiss roll histological orientation. Created in BioRender. Muegge, B. (2026) https://BioRender.com/g4khpv1. **g** Overlay of mouse small intestine data, colored by unrolled *x*-axis based on cells in the muscle layer (*μ*m). **h** Expression of selected proximal and distal enriched genes in the original ST image and histogram plot of those genes on the unrolled *x*-axis of the mouse small intestine. **i** Illustration of the crypt-villus axis in the mouse small intestine. **j** Schematic of four common villus structures encountered during construction of the *y*-axis for mouse small intestine data. **k**, Overlay of intestinal manifold in selected villi with cells colored by normalized, corrected *y*-axis. **l**, Cell-type distributions along the corrected *y*-axis in one mouse small intestine Visium HD section (biological replicate *n* = 1). Each violin shows the corrected *y*-axis positions of individual cells per type; widths indicate cell density. The overlaid box plot shows the median, first and third quartiles, and whiskers extending to the most extreme values within 1.5 × the interquartile range. The red dashed line indicates the approximate crypt-villus boundary. Panels g-l use coordinates computed across the full tissue section. Abbreviations: TA transit-amplifying, EEC enteroendocrine cell.

We evaluated SMURF’s manifold learning capabilities by analyzing the mouse brain cortex. We first identified a one-dimensional manifold path representing the spatial distribution of cells in the cortical basement membrane using a locally linear embedding algorithm^33^ (Fig. 3a). We then extended the manifold to two dimensions to include all cortical cells, assigning each cell a pair of (*x*, *y*) coordinates based on its position relative to the basement membrane (Fig. 3b, c). This effectively projected the cortex onto a rectilinear coordinate system, unrolling the tissue for spatial analysis.

To illustrate how SMURF’s manifold algorithm can reveal regional patterning of cell types and gene expression, we divided the unrolled *y*-axis of the cortex into six bins spanning from the cortical base to the surface. We plotted the expression of known cortical layer markers across these bins as a heatmap (Fig. 3d)^34^. Layer-specific gene expression aligned precisely with SMURF-assigned locations: markers of layers 2-4 neurons, such as *Cux1* and *Pou3f2*, were enriched nearer the cortical surface, while markers of layers 5 and 6, including *Bcl11b* and *Foxp2*, were expressed near the base. Next, we assigned every cortical cell in the ST data a cell-type identity based on its transcriptional profile, using the Allen Brain Cell Atlas as a reference^35,36^. As expected, cell types stratified along the projected *y*-axis, with Layer 2 and 3 neurons at the cortical surface and Layer 6 neurons at the base (Fig. 3e). Non-neuronal cell types also projected according to their known anatomic distributions (Supplementary Fig. 9)^37,38^. Taken together, these results demonstrate that SMURF accurately unrolls a manifold of cells onto rectilinear coordinates while preserving patterns of gene expression and cellular organization.

### SMURF accurately transforms complex and discontinuous tissue onto rectilinear coordinates

We next applied SMURF to investigate regional transcriptional programs in the mouse small intestine, a tissue characterized by complex geometry and continuous spatial gradients. Gene expression, cell-type composition, and physiological function vary systematically along both the proximal-distal (longitudinal) and crypt-villus (radial) axes. Despite intense research, defining the factors that shape the spatial organization of the intestine remains a challenge. Important studies using Laser Capture Microdissection (LCM) or early generations of ST technologies revealed interesting transcriptional dynamics along the crypt-to-villus or longitudinal axes but were limited because they lacked single-cell resolution^39–43^.

We analyzed Visium HD data from the healthy mouse ileum (distal small intestine) prepared in a Swiss roll configuration (Fig. 3f)^27^. SMURF soft-segmented over 400,000 cells and applied its generalized unrolling algorithm to reconstruct the tissue’s native longitudinal layout (Fig. 3g). Pseudobulked epithelial gene expression along the reconstructed *x*-axis correlated strongly with a recently published single-cell RNA-seq atlas of the mouse small intestine (Supplementary Fig. 10b)^31^. SMURF accurately localized canonical regional markers, including proximally enriched Lactase (*Lct*) and distally enriched Ileal bile acid transporter (*Ibat*) (Fig. 3h).

We next assigned cells to coordinates along the crypt-to-villus axis of the intestine. This dataset contained over 1000 villi and presented several analytical challenges (Fig. 3i). Individual villi often bend from perpendicular alignment with the muscularis base, and some are partially severed or completely detached during sectioning. To correct these distortions, we developed an iterative method inspired by dynamic programming that identifies sequential layers of cells to reconstruct a corrected villus-specific *y*-axis representing a projected perpendicular alignment for each villus (Fig. 3k, Supplementary Fig. 10c and 11; Methods). SMURF accurately localized expected cell types along this reconstructed axis: neuronal and muscle cells in the muscularis propria underneath the epithelium, stem and Paneth cells at the crypt base, proliferative transit-amplifying (TA) cells above the stem cell zone, and differentiated epithelial populations along the villus (Fig. 3i). Marker genes exhibited the anticipated spatial order, with *Olfm4* marking stem cells in the crypt, *Krt19*-expressing TA cells above the crypt, *Fabp2* enterocytes along the villus, and mature *Ada*-expressing cells at the villus tip (Supplementary Figs. 10d, e). Interestingly, some enteroendocrine and tuft cells were detected near the base of the TA zone, suggesting occasional escape from the typical upward migration of differentiated cells. While retrograde migration has been reported for EECs^44,45^, it has not previously been described for tuft cells. Collectively, these results show that SMURF precisely defines manifolds and unrolls complex and discontinuous tissue structures, projecting cells onto rectilinear coordinates that preserve in vivo spatial relationships, even in regions that are bent, torn, or partially missing.

### SMURF projects high-resolution ST data from multiple platforms and tissues

**Fig. 4:**
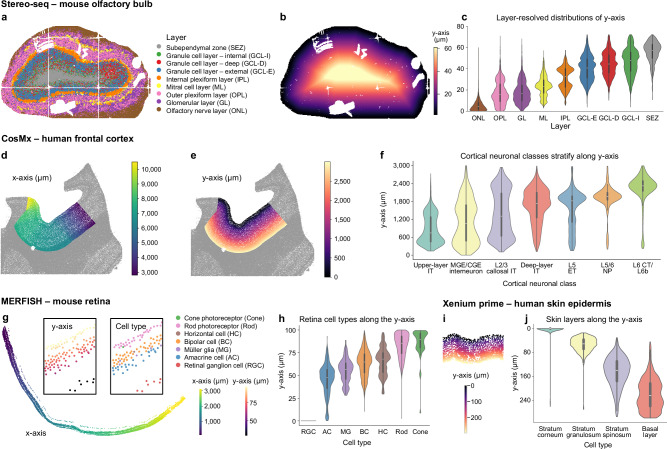
SMURF accurately projects high-resolution ST data from multiple platforms and tissues. **a–c** Stereo-seq, mouse olfactory bulb. **a** Tissue map with spots colored by annotated layers. **b** Unrolled coordinate field showing *y*-axis manifold position across the olfactory bulb. **c** Layer-resolved distributions of the unrolled *y*-axis. **d–f** CosMx, human frontal cortex. **d**,**e** Segmented cells with colors indicating the unrolled *x*-axis or *y*-axis coordinate (*μ*m). **f** Distributions of cortical neuronal classes on the unrolled *y*-axis. **g**,**h** MERFISH, mouse retina. **g** Low-dimensional manifold of retinal cells colored by *x*-axis (left to right) and *y*-axis (inner to outer), with insets showing the same embedding labeled by major cell classes. **h** Distributions of cell types on the unrolled *y*-axis. **i**,**j** Xenium Prime, human skin epidermis. **i**, Segmented epidermal cells colored by the unrolled *y*-axis (*μ*m), using the outermost stratum corneum as the manifold base. **j** Distributions of the unrolled *y*-axis across epidermal strata. In all violin plots (**c**,**f**,**h**,**j**), violin width represents the density of observations, with a single cell assigned a *y*-axis coordinate by SMURF. Box plots show the median, first and third quartiles, and whiskers extending to the most extreme values within 1.5 × the interquartile range; outliers beyond whiskers are not shown. Each analysis used one tissue sample or section from the corresponding public dataset (biological replicate *n* = 1). Where applicable, cell-type labels were taken from the original dataset annotations. Abbreviations: AC amacrine cell, BC bipolar cell, CGE caudal ganglionic eminence, CT corticothalamic, ET extratelencephalic, GCL-D granule cell layer-deep, GCL-E granule cell layer-external, GCL-I granule cell layer-internal, GL glomerular layer, HC horizontal cell, IPL internal plexiform layer, IT intratelencephalic, L2/3 cortical layers 2/3, L5 cortical layer 5, L5/6 cortical layers 5/6, L6 cortical layer 6, L6b cortical layer 6b, MERFISH multiplexed error-robust fluorescence in situ hybridization, MGE medial ganglionic eminence, MG Müller glia, ML mitral cell layer, NP near-projecting, ONL olfactory nerve layer, OPL outer plexiform layer, RGC retinal ganglion cell, SEZ subependymal zone.

We next asked whether SMURF’s unrolling and projection algorithms generalize across different high-resolution ST platforms. We first applied SMURF to another sequencing-based method, Stereo-seq, using data from the mouse olfactory bulb^2^. The olfactory bulb is organized concentrically into ring-like layers (Fig. 4a). Using the authors’ provided cell segmentation, we anchored the SMURF projection on the outermost olfactory nerve layer and unwrapped the tissue into a two-dimensional coordinate space (Fig. 4b). SMURF successfully resolved the expected radial progression of cell types from outermost to innermost, including the glomerular layer, mitral cell layer, and deep granule cell layer (Fig. 4c). These results demonstrate that SMURF generalizes across other sequencing-based ST platforms and complex tissue geometries.

Sequencing-based technologies, such as Visium HD and Stereo-seq, capture and amplify mRNAs using spatially barcoded oligonucleotides that are mapped to histologic images to infer location^1,6^. In contrast, imaging-based ST methods directly quantify gene expression in situ at subcellular resolution using labeled probes. To test SMURF’s performance on these datasets, we applied it to several imaging-based platforms, including CosMx, MERFISH, and 10x Xenium Prime^46–48^. We used the supplied cell segmentations and applied SMURF’s manifold unrolling algorithm to project each dataset into standardized Cartesian coordinates. Using CosMx data from the human frontal cortex, SMURF successfully recapitulated the laminar organization of cortical neurons^49^. The SMURF *y*-axis stratified excitatory and inhibitory neuronal subclasses in line with known cortical layers (Fig. 4d–f). We next analyzed a MERFISH mouse retina dataset^48^. Anchoring SMURF’s manifold learning on the innermost retinal layer (ganglion cells) revealed the expected organization of cell types along the *y*-axis: ganglion and amacrine cells at the base, bipolar and horizontal cells in the inner nuclear layer, and photoreceptor rods and cones in the outermost layer (Fig. 4g, h). Finally, we applied SMURF to a 10x Xenium Prime human skin melanoma dataset, focusing on the non-malignant epidermis^50^. We annotated epidermal keratinocyte subtypes using canonical marker genes^51^. SMURF unrolled and correctly ordered cells along the stratified epidermal axis from the stratum corneum at the surface, through the stratum granulosum and stratum spinosum, to the basal layer at the epidermal-dermal junction (Fig. 4i, j). Extending this analysis, SMURF projected the tissue along an isodepth axis anchored at the basal layer, revealing distinct layer-specific organization near the surface, while deeper regions beyond the basal boundary lacked clear laminar structure (Supplementary Fig. 12). These results demonstrate that SMURF can integrate data collected using a variety of ST platforms across a number of tissue types into a common coordinate space, enabling the quantitative analysis of cell organization and gene-expression gradients in complex tissue architectures.

### SMURF identifies the zonation of transcriptional programs in intestinal development

**Fig. 5:**
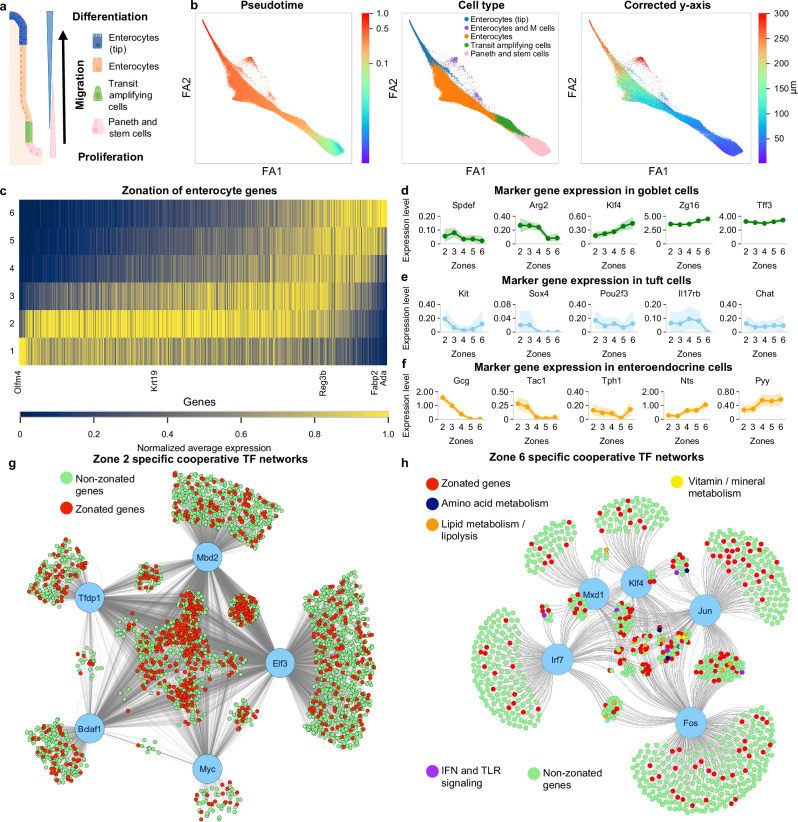
Spatial, temporal, and transcription-factor program organization along the ileal crypt-villus axis. **a** Schematic illustrating the differentiation trajectory of the epithelial lineage in intestinal villi from proliferative stem cells (bottom) to differentiated enterocytes (top). Created in BioRender. Muegge, B. (2026) https://BioRender.com/ff4xl16. **b** Concordance of pseudotime trajectory and corrected *y*-axis position during epithelial maturation. PAGA embedding of crypt-base Paneth/stem cells, TA cells, and enterocytes (*n* = 139,637 cells), colored by pseudotime position (left), cell type (center) and corrected *y*-axis position (right). **c** Heatmap of normalized gene expression across zones. Gene expression in all stem and Paneth cells, TA cells and enterocytes is included. The heatmap displays normalized expression of each gene (columns) in each zone (rows). **d–f** Zonation profiles of selected genes in goblet cells (*n* = 4006) (**d**), tuft cells (*n* = 731) (**e**), and EECs (*n* = 1297) (**f**). In (**d–f**), zonation boundaries were first defined from intact villi using the normalized corrected *y*-axis. Cells of each cell type were then assigned to zones according to their normalized corrected *y*-axis positions relative to these predefined boundaries. For each cell type and zone, expression values were summarized as the mean across cells in that zone. Shaded regions indicate 95% bootstrap confidence intervals for the mean, calculated by resampling cells within each zone with replacement (500 iterations). **g** Integrated TF target networks for Zone 2. TFs are depicted in blue circles. Lines connect TFs to their gene targets. Selected target genes are color-coded by their predominant functional category. **h** Integrated TF target networks for Zone 6. Coloring is as in (**g**). Stem and Paneth cells, TA cells, and enterocytes of intact villi are included in panels g and h. Abbreviations: TA transit-amplifying, EEC enteroendocrine cell, TF transcription factor, PAGA partition-based graph abstraction.

To demonstrate the potential of SMURF to discover biological insights, we returned to the intestine. Stem cells at the crypt base continuously give rise to rapidly dividing TA cells, which differentiate into specialized digestive and barrier functions as they migrate up the villus (Fig. 5a)^52^. We hypothesized that SMURF’s projection of complex tissue structures onto rectilinear coordinates could be used to quantitatively examine the relationship between intestinal enterocytes’ developmental state and their spatial position on the crypt-villus axis. Pseudotime trajectories were measured using diffusion maps and visualized with partition-based graph abstraction (PAGA) and force-directed graph layouts (Fig. 5b)^53–55^. The pseudotime ordering captured a continuous transition from the crypt-base epithelial compartment through TA cells toward mature villus enterocyte states (Fig. 5b, left and middle panels). SMURF-assigned *y*-axis position followed a similar distribution (Fig.5b, right panel), and was significantly correlated with pseudotime (two-sided Pearson correlation, *corr* = 0.78, *P* value  < 10^−10^, *n* = 139,637*cells*; Supplementary Fig. 13a). These findings demonstrate SMURF can recapitulate the spatial-temporal coupling of cell differentiation along the crypt-villus axis solely from spatial location.

We next sought to identify the gene programs that change along the crypt-villus axis during epithelial cell maturation in the ileum. We binned cells from intact villi into six zones spanning from the crypt to the villus tip, based on their corrected *y*-axis positions (Supplementary Fig. 13b). We chose six zones so that we could directly compare our results to a previous LCM study of jejunal zonation^39^. Using cells from stem/Paneth, TA, and enterocyte populations within this section, we observed strong zone-associated differences in gene expression across the crypt-villus axis (Fig. 5c). Using pairwise cell-level comparisons of each zone against all other zones, 2846 genes met our predefined criteria for zone-associated differential expression (Methods, Supplementary Data 1). We further refined this list to a set of landmark genes highly specific for each zone by algorithmic and manual curation (Supplementary Fig. 14, Supplementary Note). KEGG pathway enrichment analysis revealed specialized transcriptional programs in each zone, including WNT signaling and proliferation in the crypts, with emerging capacities for carbohydrate, protein, and lipid absorption as enterocytes matured along the villus (Supplementary Fig. 13c). Recent reports in the mouse jejunum and human duodenum showed functional specialization along the crypt-villus, but the genes and pathways in the ileum are distinct, highlighting the regionally specialized nature of intestinal metabolism^39,56^.

We next turned our attention to the crypt-to-villus zonation of less abundant secretory epithelial cell types. Goblet cells, tuft cells, and the heterogeneous EECs are individually scattered throughout the villus and cannot be isolated by LCM or conventional spatial analysis with large bin sizes. Using the same six zones, SMURF analysis revealed clear zone-associated expression patterns along the crypt-to-villus axis for each of these secretory cell types (Fig. 5d–f). In contrast to a recent study of the human small intestine that showed tuft cell markers zonated towards the bottom of the villus^56^, the markers were variably expressed along the crypt-villus axis in mice. The broad tuft marker *Kit* was enriched at the bottom of the villus. *Sox4* is known to initiate the tuft cell program, including the lineage-specifying TF *Pou2f3*. SMURF projections recovered this relationship with *Sox4* at the base, and *Pou2f3* was broadly expressed (Fig. 5e)^57^. Immune and sensory receptors, including *Il17rb* and *Chat*, had variable expression along the villus axis, in line with recent reports of spatial segregation of tuft cell types in the intestine (Fig. 5e)^58–61^. Similarly, EEC hormone expression was highly zonated. EECs in the villus base expressed Substance P (*Tac1*) and GLP-1 (*Gcg*), while EECs expressing Neurotensin (*Nts*) and Peptide YY (*Pyy*) were only detected in villus zones, consistent with prior immunohistochemical studies (Fig. 5f)^62,63^.

This analysis describes ileal transcriptional zonation using ST data and provides a resource of landmark genes in this organ. The functional progression of metabolic functions in enterocytes is concordant with prior studies in the jejunum^39^, but our ST methods additionally resolve zonation in multiple rare cell types.

### Zonated transcription factor regulatory networks along the villus

The network of transcription factors regulating zonated gene expression in the mature villus remains poorly defined. Using SCENIC, we identified active TFs and their targets in each zone based on expression profiles from stem/Paneth, TA, and enterocyte cells^64^. We detected 187 TFs with average activity scores in at least one of the six zones ≥ 0.05, (Supplementary Note, Supplementary Table 4).

Cooperative TF binding is a well-established mechanism for regulating gene expression; to identify putative cooperative interactions governing the zonation of gene expression in the ileum, we mapped TF interaction networks within each zone. In Zone 2, a highly interconnected network of five TFs emerged, anchored by *Elf3* (Fig. 5g). *Elf3* mediates *Wnt*-independent *β*-catenin activation in colon cancer^65,66^. Other members of this network include *Myc* and *Tfdp1*, which are known to regulate proliferation and cell-cycle progression in the intestine, as well as *Bclaf1*, and *Mbd2*, which are not as well studied in intestinal zonation^67–69^. Genes with enriched expression in Zone 2 were significantly more likely to be targets of multiple TFs in this network (*P* value = 2.03 × 10^−9^; Fig. 5g, Methods). Genes co-targeted by two or more TFs were enriched for RNA biogenesis and mitochondrial metabolism, core TA cell functions. Thus, we identified a cooperative TF network associated with TA cell proliferation.

At the villus tip, we identified a distinct TF network, including AP-1 complex members *Jun* and *Fos*, previously linked to tip identity, as well as *Irf7*, *Mxd1*, and *Klf4*^39,56,70,71^. As in Zone 2, tip-zonated genes in Zone 6 were also over-represented among those targeted by multiple TFs (*P* value  < 10^−10^; Fig. 5h). These genes are involved in amino acid, lipid, and vitamin metabolism, as well as host defense, suggesting that AP-1 complex and environmental sensors, such as *Irf7* cooperate to drive tip-specific functions.

### Regionally specific gene programs accumulate in the upper epithelial villus

**Fig. 6:**
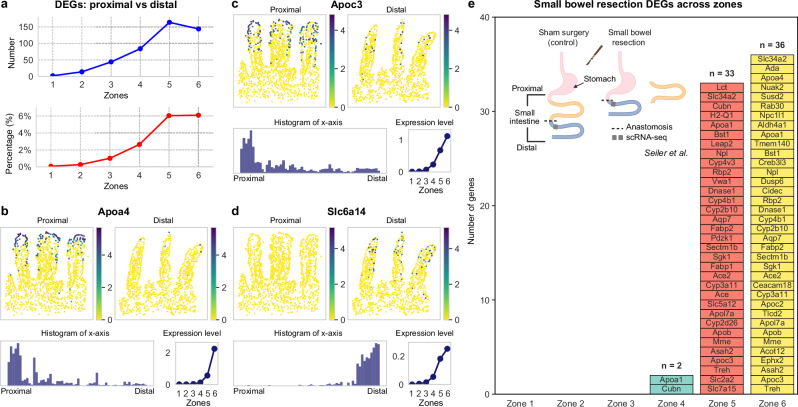
Regionally specific gene programs along the proximal-distal intestinal axis accumulate in the villus tip. **a** Number (top) and fraction (bottom) of genes differentially expressed between proximal and distal regions of villi within each zone. Within each zone, mitochondrial genes were excluded, and only genes expressed in at least 100 cells were considered. Genes were retained when absolute ${\log }_{2}$ fold-change exceeded 1.5 and adjusted *P* < 0.001. Fractions were calculated relative to the number of genes retained after filtering in each zone. **b** Expression of proximal-tip enriched gene *Apoa4*. Data show expression in the original ST image, histogram of expression along the unrolled *x*-axis, and expression across zones. **c** Expression of proximal-tip enriched gene *Apoc3*. Data are shown as in (**b**). **d** Expression of distal-tip enriched gene *Slc6a14*. Data are shown as in (**b**). **e** Distribution of SBR-associated differentially expressed genes across healthy ileum zones. Created in BioRender. Muegge, B. (2026) https://BioRender.com/b5ohlm7. Abbreviation: DEG, differentially expressed gene.

The intestine is functionally specialized along its proximal-distal axis, yet the molecular mechanisms that maintain regional identity remain unclear. To address this, we divided cells from intact villi in the ileal section into proximal and distal groups according to the *x*-axis and performed differential-expression analysis comparing proximal and distal cells within each zone. The zones of the upper villus displayed considerably more proximal-to-distal differential gene expression than zones at the base, suggesting that transcriptional programs that vary along the length of the ileum are concentrated in the villus tips (Fig. 6a)^11,31,72^. Our ST data are derived from the mouse ileum. To test the idea that villus bottom zonation is preserved across regions while villus top zonation is regionally specific, we plotted markers of jejunal crypt-villus zonation identified previously by LCM (Supplementary Fig. 15a, b)^39^. The genes identified as bottom markers in the jejunum were also expressed predominantly in Zones 2 and 3 of the ileum. In contrast, the jejunal villus top markers were not segregated at the top of the ileum, supporting the idea that villus tips have regionally specific transcriptional programs. Proximal ileal villus tips upregulated genes involved in typical proximal metabolic pathways, including lipid transporters *Apoa4* and *Apoc3*, consistent with the proximal ileum’s role in absorbing excess dietary fats (Fig. 6b, c)^73^. In contrast, distal ileal tips are enriched for known distal ileum genes, such as the beta-alanine transporter *Slc6a14*, along with genes involved in bile acid absorption and steroid biosynthesis (Fig. 6d)^74^.

These data suggest that proximal-distal cellular identity is not dictated solely by stem cell-intrinsic transcriptional programs. Although intestinal stem cells have been thought to encode much of their progeny’s regional identity, recent evidence challenges this view^31,75,76^. We therefore hypothesized that luminal gradients of environmental signals, including dietary components, xenobiotics, and microbes, interact with intestinal TF networks to establish regional identity.

To test this, we examined a small bowel resection (SBR) model, in which 50% of the small intestine is surgically removed, and the remaining segments are joined, exposing the distal ileum to proximal luminal contents (Fig. 6e)^77^. Our hypothesis predicts that this exposure would induce proximal transcriptional programs in the upper zones of distal villi. We re-analyzed previously reported scRNA-seq from SBR ileal tissue and sham-operated controls, identified differentially expressed genes, and mapped these genes to spatial zones in the healthy ileum using our ST dataset. As predicted, genes upregulated after resection showed a non-random distribution across spatial zones, with the strongest enrichment in upper-villus zonated genes (two-sided permutation-based chi-square-like enrichment test, *P* value  = 9.99 × 10^−6^; Fig. 6e). None of the induced genes were expressed in stem and TA cells, indicating that most transcriptional changes occur downstream of any stem cell reprogramming. As reported, the SBR-upregulated genes were enriched for proximal lipid metabolism pathways^77^. Together, these results support our hypothesis that environmental cues, rather than stem cell-intrinsic programs alone, shape regional identity. However, we cannot exclude additional effects from epithelial proliferation or hormonal signaling triggered by surgery.

## Discussion

The emergence of high-resolution, capture-based ST platforms has created an urgent need for algorithms that can accurately assign transcripts in densely tiled capture spots to individual cells. Here, we showed that SMURF’s soft-segmentation method accurately and comprehensively assigns mRNAs to cells, segments cells with high accuracy, and reliably detects rare cell types. SMURF also unrolls tissue substructures, even when parts of them are damaged or missing, placing cells on Cartesian coordinates, thus enabling the quantitative analysis of the in vivo spatial organization of cell types and transcriptional programs. SMURF’s unrolling functionality generalizes across platforms, as demonstrated by the successful application to datasets from five ST technologies: Visium HD, Stereo-seq, CosMx, MERFISH, and Xenium Prime.

We applied SMURF to analyze ST data from the small intestine and characterize the spatial organization of this organ. Prior work in this area had revealed important principles, but was constrained by coarse resolution or the use of labor-intensive methods^39,40,42,56,78^. Using SMURF, we identified cell-type-specific zonated gene expression programs and TF regulatory networks along the crypt-villus axis. We also used SMURF to investigate a fundamental question about small intestine development: are proximal-to-distal gradients in gene expression predetermined or established in response to environmental signals? We found that regional gene expression differences are largely restricted to upper villus cells and can be reprogrammed by relocating the distal intestine to a proximal environment, demonstrating that proximal-distal patterning in adult tissue is shaped more by environmental cues than by intrinsic stem cell programming.

SMURF’s segmentation algorithm has some caveats. Because Visium HD does not directly provide plasma-membrane boundaries, transcript assignment can be more challenging for large columnar or elongated cells. While SMURF can assign spots to cells asymmetrically, this process depends on a user-defined parameter that specifies the maximum expansion distance from the nucleus, which may need to be tuned to maximize the capture of cytoplasmic transcripts. Another limitation inherent to sequencing-based ST datasets like those produced by Visium HD is that the low number of UMIs captured per cell limits sensitivity to detect lowly expressed genes and increases uncertainty in downstream analyses, including annotation of rare populations or closely related subtypes, because of marker dropout (Supplementary Note).

One limitation of SMURF’s unrolling algorithm is that, while it enabled biological insights that could be validated using published data in the intestine, it is difficult to rigorously benchmark how well SMURF’s unrolling algorithm resolves the fine structure of tissues because there are few gold-standard reference datasets for such analysis. Despite this limitation, we show that SMURF’s output is highly consistent with biological expectations. Another caveat with our analysis is that spatial zonation of mRNA transcripts does not always correspond directly to lineage progression or protein-level function. For enterocytes, maturation and zonation are broadly aligned, but for some secretory cell types, such as Paneth cells, this relationship is obfuscated by the retrograde migration of these cells^79–82^. In addition, spatial transcriptomic data measure RNA and not protein abundance, and these are not perfectly correlated^41^. Our analysis should therefore be interpreted as describing the zonation of transcript abundance rather than protein function.

As ST continues to evolve, new platforms will employ even higher-resolution capture spots (for capture methods), analyze more genes (for imaging-based methods), and profile larger numbers of cells. The full potential of these technologies will only be achieved with approaches like SMURF that consider all capture spots, have the ability to assign mRNAs in the same capture spot to different cells, and can unroll tissues in a platform-agnostic manner. SMURF’s segmentation method is inherently extensible to emerging technologies for three-dimensional tissue ST. SMURF’s segmentation and unrolling approach complements existing methods to study signaling gradients in ST^83–85^. Finally, SMURF’s unrolling algorithm will likely be adaptable to protein- or metabolite-based spatial technologies, although this will require further validation on high-quality datasets (Supplementary Fig. 16).

Our study highlights SMURF’s utility to uncover principles of tissue organization during normal development. An important future direction will be to deploy this approach to study disease models at single-cell resolution. Many diseases disrupt normal tissue structures, such as the loss of normal crypt-villus architecture in inflammatory bowel disease, or additional cell populations, such as vascular and immune cells in cancer. SMURF offers a framework to dissect how multiple cell types interact in these contexts, including 4D studies of evolving responses over time. We have demonstrated SMURF’s utility in multiple tissues (Supplementary Note) and anticipate that SMURF will provide insight into other spatially complex organs, such as the kidney, retina, and lung.

## Methods

### Soft segmentation analysis

#### Introduction

In this section, we provide a detailed description of the soft-segmentation method implemented in SMURF. Cells are denoted by the index *i*, genes by *j*, and spots by *k*. Since cells are typically associated with exactly one nucleus, we denote both a cell and its corresponding nucleus by *i*, emphasizing their one-to-one correspondence. The inputs to the soft segmentation algorithm consist of: (1) the 2 *μ*m spot gene expression matrix *S*; (2) the H&E or DAPI/IF tissue image Img^*o*^; and (3) the spot-to-pixel data frame.

We define the 2 μm spot gene expression matrix *S* to have dimensions *K*_spots_ × *J*_genes_, where *K*_spots_ represents the number of spots and *J*_genes_ the number of genes, with each element *S*_*kj*_≥0. Each pixel in the original image Img^*o*^ represents a grayscale value (0–255) for DAPI/IF images or an RGB array for H&E-stained images. The matrix *S* is linked to the processed image Img via the spot-to-pixel data frame. We limited our analysis to the section of the tissue bounded by the Visium HD capture area, defined as Img. We denote by Img_*k*_ the set of pixels corresponding to spot *k*.

SMURF requires users to generate their own nuclei segmentation results. To facilitate this, we provide tutorials for DAPI/IF images (using Cellpose v3.0.5^15,86^) and H&E-stained images (using StarDist v0.8.5^16–18^); users may also choose their preferred segmentation tools. The final nuclei segmentation output, denoted as Nuclei_img, is a two-dimensional matrix with the same dimensions as Img. In Nuclei_img, regions without nuclei are assigned a value of 0, whereas regions containing nuclei receive unique numerical identifiers.

Given the substantial volume of data and resulting outputs, SMURF defines a Python object, the Spatial Object (SO), initially constructed from the three inputs. The SO records the nuclei segmentation results, detailed information for each cell, and visualization outputs. For clarity, we refer to the components of SO by their object names rather than describing each element of SO here.

The soft-segmentation in SMURF involves three main steps: nuclei analysis, cell expansion, and count deconvolution for shared spots. For the first step, a nucleus-by-gene matrix is constructed and clustered to provide initial cell type annotations, and a nearest neighbor network is constructed to estimate approximate cell size; second, cells are expanded by iteratively assigning adjacent spots based on their similarity to the cell’s cluster identity, with periodic reclustering to refine assignments; finally, overlapping spots that are shared by multiple cells are resolved through a combination of quadratic programming and a custom deep-learning module implemented in PyTorch (v2.2.1), which learns soft assignments for candidate cells and reconstructs accurate gene-expression profiles for each cell. Finally, SMURF outputs a spatial single-cell AnnData object containing the gene expression matrix *G* (*I*_cells_ × *J*_genes_). Each cell is annotated with its gene expression profile, spatial coordinates, preliminary cluster assignment, and the cosine similarity to its assigned cluster.

#### Step one: nuclei analysis

The nuclei analysis includes mainly three parts: building the nuclei gene expression array, clustering nuclei, and estimating the potential sizes of cells based on location. It takes in the 2 μm spot gene expression matrix *S* and the nuclei segmentation results Nuclei_img from the image.

Before the analysis, we first filter the 2 μm spot gene expression matrix *S* based on the criterion

$${\sum }_{k=1}^{{n}_{{{{\rm{spots}}}}}}{S}_{kj}\ge {mincounts}\,,$$

with the default threshold set at mincounts = 1000. We denote the resulting filtered matrix as ${S}^{{\prime} }$.

The Nuclei_img contains pixel-level data, and each 2 μm spot corresponds to approximately 50 pixels. Consequently, many nuclei overlap multiple capture spots and, conversely, some capture spots are shared by multiple nuclei. We define pct_*ik*_ = (number of pixels in nucleus*i* of spot*k*)/Img_*k*_, with 0≤pct_*ik*_≤1, to denote the percentage of pixels in spot *k* that overlap the nucleus of cell *i*. For each nucleus *i*, we record all spot IDs for which the fraction of overlapping pixels is at least a user-adjustable minimum threshold (default =1/6). The transcripts captured in all spots that meet this criterion for nucleus *i* are assigned to that nucleus. Using this initial assignment of transcripts to cells, we create the gene count matrix *X*^(0)^, defined as:

$${X}_{ij}^{(0)}={\sum }_{k=1}^{{n}_{{{{\rm{spots}}}}}}{{{\bf{1}}}}({{{{\rm{pct}}}}}_{ik}\ge 1/6)\times {S}_{kj}^{{\prime} }$$

where *X*^(0)^ is an *I*_cells_ × *J*_genes_filtered_ matrix and ${X}_{ij}^{(0)}$ indicates the count expression of gene *j* in nucleus *i*.

In the next part, we filter out nuclei with few transcripts assigned to them, since extremely low counts could be due to errors like water drops on the capture surface and influence the accuracy of further analysis. We then analyze matrix *X*^(0)^ using the standard Scanpy^19,20^ workflow, including filtering out genes that are not expressed in some minimum number of cells, count normalization, dimensionality reduction via principal component analysis (PCA), and Leiden clustering. For this initial clustering step, we use a default Leiden resolution of 2.0, although this parameter is user-adjustable and may be tuned according to dataset complexity and the desired clustering granularity. For these analyses, we followed standard Scanpy/Seurat best practices^19,87^. Through this approach, we obtain initial clustering results *C*^(0)^ based on the *X*^(0)^, where *C*^(0)^ is a 1*n_cells_ array that assigns each nucleus into one of *n*^(0)^ clusters based on gene expression profile. These initial cluster assignments are based solely on transcripts assigned to nuclei from overlapping capture spots.

Finally, we estimate the potential number of spots belonging to a cell based on its cell density. We calculate the center of each nucleus by averaging the locations of the identified spots associated with that nucleus. We employ the k-nearest neighbors (KNN) algorithm^88^, linking each nucleus to its three closest neighbors. For each nucleus *i*, we record size_*i*_ as the number of spots occupied by the nucleus and the distances *d*_*io*_ (for *o* = 1, 2, 3) from the center of nucleus *i* to the centers of its three nearest neighbors. Assuming that the nuclei are approximately circular, we calculate ${d}_{i}^{{\prime} }$ as:

$${d}_{i}^{{\prime} }=\frac{1}{3}{\sum }_{o=1}^{3}{d}_{io}-2\sqrt{\frac{{{{{\rm{size}}}}}_{i}}{\pi }},$$

which represents the average distance between nearby cells minus twice the estimated radius (calculated from the cell size), thus approximating the maximum extent of the spots.

#### Step two: cell expansion

Having assigned transcripts to nuclei from all overlapping capture spots, we next sought to assign transcripts from nearby, non-overlapping capture spots, effectively expanding these nuclei into cells. During this nuclei expansion phase, we use an alternating optimization strategy to expand the nuclei, which generates robust cell gene profiles and reliable clustering results. In essence, we iteratively assign adjacent spots to cells based on whether their addition increases the cell’s similarity to the cluster to which it is assigned. We perform periodic reclustering to refine the assignments.

Here, we use the iteration variable *r* (starting from *r* = 1) to record each iteration and define the initial nuclei results as the starting point (*r* = 0). For example, as previously introduced, *X*^(0)^ represents the nuclei-by-gene expression matrix, *C*^(0)^ denotes the nuclei clustering results.

In the beginning of each iteration *r*, we calculate the average expression of each cluster, denoted by *w*^(*r*−1)^, which has dimensions *n*^(*r*−1)^ × *n*_genes_filtered_. For each cluster $c=1,2,\ldots,{n}_{c}^{(r-1)}$ and for each gene *j*, the average expression is defined as

$${w}_{cj}^{(r-1)}=\frac{1}{\parallel {C}_{c}^{(r-1)}\parallel }{\sum}_{i\in {C}_{c}^{(r-1)}}{X}_{ij}^{(r-1)},$$

where ${C}_{c}^{(r-1)}$ denotes the set of cells belonging to cluster *c* in *C*^(*r*−1)^ and $\parallel {C}_{c}^{(r-1)}\parallel$ represents the number of cells in this set. Then, we normalize the gene expression by

$${\hat{w}}_{c}^{(r-1)}=\frac{{w}_{c}^{(r-1)}}{\parallel {w}_{c}^{(r-1)}\parallel }.$$

Additionally, we calculate

$${\bar{d}}_{c}^{(r-1)}=\frac{1}{\parallel {C}_{c}^{(r-1)}\parallel }{\sum}_{i\in {C}_{c}^{(r-1)}}{d}_{i}^{{\prime} },$$

to decide the maximum expansion of spots for each nuclei. By default, we use

$${\tilde{d}}_{c}^{(r-1)}=\max \left(1,\min \left(2,\lceil{\bar{d}}_{c}^{(r-1)}\rceil+1\right)\right).$$

In the second stage, our goal is to derive the matrix *X*^(*r*)^(*n*_cells_ × *n*_genes_filtered_) so that cells within the same cluster exhibit high cosine similarity. We denote the gene expression vector for cell *i* in the current iteration as ${{{{\bf{X}}}}}_{i}^{(r)}$ and let ${{{{\rm{cell}}}}}_{i}^{r}$ represent the spots assigned to cell *i* that were not previously associated with nucleus *i* in iteration *r*. The objective function is defined as follows:

1 $${\Phi }_{1}({X}^{(r)})={\sum }_{c=1}^{{n}_{c}^{(r-1)}}{\sum }_{i\in {C}_{c}^{(r-1)}}{cos}_{{{{\rm{sim}}}}}({{{{\bf{X}}}}}_{i}^{(r)},{\hat{w}}_{c}^{(r-1)})\\={\sum }_{c=1}^{{n}_{c}^{(r-1)}}{\sum }_{i\in {C}_{c}^{(r-1)}}{cos}_{{{{\rm{sim}}}}}\left({X}_{i}^{(0)}+{\sum }_{k\in {{\mbox{cell}}}_{i}^{r}}{S}_{k}^{{\prime} },{\hat{w}}_{c}^{(r-1)}\right)$$

Here, our objective is to determine ${{{{\rm{cell}}}}}_{i}^{r}$ for *i* = 1, 2, …, *n*_cells_. For each spot *k* included in ${{{{\rm{cell}}}}}_{i}^{r}$, the distance from the spot to the nucleus must be less than ${\tilde{d}}_{c}^{(r-1)}$, and the spots within a cell must form a connected region.

To achieve this within limited memory resources, we employ a greedy algorithm that expands the spots one by one in a specified order. A spot is added if its inclusion improves the objective function (1), and expansion in a particular direction ceases if the previously added spot is excluded.

In the next phase of iteration *r*, we analyze *X*^(*r*)^ following the Scanpy tutorial as described earlier, thereby obtaining the clustering information *C*^(*r*)^. To compare the clustering outcomes between iterations *r* and *r* − 1, we calculate the normalized mutual information score (NMI) using the formula:

$${NMI}\,({C}^{(r)},{C}^{(r-1)})=\frac{2\times I({C}^{(r)};{C}^{(r-1)})}{H({C}^{(r)})+H({C}^{(r-1)})}$$

where *I*(*C*^(*r*)^; *C*^(*r*−1)^) denotes the mutual information between the clustering iterations, defined as:

$$I({C}^{(r)};{C}^{(r-1)})=\mathop{\sum}_{u\in {C}^{(r)}}\mathop{\sum}_{v\in {C}^{(r-1)}}P(u,v)\log \left(\frac{P(u,v)}{P(u)P(v)}\right),$$

*H*(*C*^(*r*)^) and *H*(*C*^(*r*−1)^) represent the Shannon entropies of the respective clustering iterations, defined as:

$$H({C}^{(r)})=-{\sum}_{u\in {C}^{(r)}}P(u)\log P(u).$$

This calculation quantifies the consistency of clustering results between iterations *r* and *r* − 1, with higher NMI values indicating greater similarity. Typically, the NMI increases as the iterations progress, and by default, the process terminates when there is no improvement in the NMI between two consecutive iterations.

#### Step three: count deconvolution for shared spots

The previous step assigns transcripts to cells from spots that do not overlap with nuclei; however, transcripts from a single spot may be assigned to multiple cells. In this step, we deconvolve counts from shared spots so that every transcript is assigned to at most one cell. We denote these assignments as *r* = *. At this stage, we isolate spots that are exclusively associated with a single cell and sum their gene counts to obtain ${X}^{{{{{\rm{final}}}}}_{0}}$. Next, we focus on the ambiguous spots, which can be categorized into two types: those shared by cells from different clusters and those shared by cells within the same cluster. We first address the former by transforming situations where multiple clusters share spots into scenarios resembling the latter case. Finally, after resolving spots shared by cells from different clusters, we optimize the refined objective function (Equation 9) using gradient-based optimization in PyTorch to determine the proportions of cells within the remaining shared spots.

For the first scenario, we use a deconvolution algorithm for transcript assignment. We simplify the problem to a case where cells from different clusters, denoted by the set *u*_*k*_, share a single spot *k*, with each cluster’s contribution represented by the weight matrix ${\hat{w}}_{c}^{*}$. Due to the resolution of Spatial technologies, *u*_*k*_ typically contains 2 or 3 clusters. We then employ sequential least-squares programming and define the equation as:

$${\sum}_{c\in {u}_{k}}{{{{\rm{pct}}}}}_{ck}\cdot {\hat{w}}_{c}^{*}={{{{\bf{S}}}}}_{k}^{{\prime} }/({\sum }_{j}^{{n}_{{{{\rm{genes}}}}}}{S}_{kj}^{{\prime} }),$$

where ${\sum }_{c\in {u}_{k}}{{{{\rm{pct}}}}}_{ck}=1$ ensures that the proportions sum up to unity and ${{{{\bf{S}}}}}_{k}^{{\prime} }$ represents the gene expression array for spot *k*. After obtaining the values of pct_*ck*_, we employ a binomial model to determine the precise count via random number generation. We then define ${X}^{{{{{\rm{final}}}}}_{1}}$ as ${\sum }_{k\in {{{{\rm{cell}}}}}_{i}\bigcap ({\mbox{i is only cell of c in k}})\bigcap (c\in {u}_{k})}{{{{\rm{pct}}}}}_{ck}*{{{{\bf{S}}}}}_{k}^{{\prime} }$.

Here, each specific cell *i* contains spots with predetermined area percentages.

However, there remains a subset of spots *v*_*k*_ that are shared by at least two cells within the same cluster. We refine the objective function (2) to incorporate both the already assigned spots and those yet to be assigned, as follows:

2 $${\Phi }_{2}(X)={\sum }_{c}^{{n}_{c}^{*}}{\sum }_{i\in {C}_{c}^{*}}{cos}_{{{{\rm{sim}}}}}\left({X}_{i}^{{{{{\rm{final}}}}}_{0}}+{X}_{i}^{{{{{\rm{final}}}}}_{1}}+{\sum }_{k\in {v}_{k}}{{{{\rm{pct}}}}}_{ik}\cdot {S}_{k}^{{\prime} },{\hat{w}}_{c}^{*}\right)$$

pct_*ik*_ satisfies the condition:

$${\sum}_{i\in (cellspartiallyinspotk)}{{{{\rm{pct}}}}}_{ik}=1.$$

The maximization of Equation (2) is achieved using a custom deep-learning module implemented in PyTorch. For each spot, we define trainable weights over the candidate cells that partially occupy that spot, while all other cells are constrained to have zero weight. These trainable weights are normalized with a softmax function so that they form valid proportions within each shared spot. The model is optimized using Adam with a cosine-similarity-based objective, in which the predicted cell-type expression matrix is encouraged to match the target cell-type expression matrix. Unless otherwise stated, optimization is run for up to 1000 epochs with a learning rate of 0.1 and early stopping when the improvement in loss falls below 1 × 10^−3^. After optimization, a multinomial model is used to convert the learned proportions into integer transcript counts for shared spots. Because each cell is represented by a set of spot-level contributions and their inferred proportions, we then return to the unfiltered matrix *S* to recover the gene-expression count matrix for all genes. For this step, the lite version of SMURF uses a spatially distance-weighted multinomial model to assign counts by random sampling.

### Unrolling analysis

Many tissues possess intricate biological structures that are challenging to study, especially since some tissues can become deformed on slides, obscuring their native organization. SMURF’s soft segmentation algorithm identifies distinct clusters of cells that include spatial information. Using these clusters to define a manifold, we propose a framework to unroll and elucidate the underlying biological architecture of tissue substructures.

We first select a specific cluster or the boundary of the cluster/tissue from the soft-segmentation output to guide the unrolling process. Because the relevant cluster may not perfectly correspond to the boundary of a tissue substructure, the set of cell coordinates can be augmented by user-defined points and by inserting the midpoints between existing cells. Cells that are isolated or located at the periphery are filtered out if their average distance to the nearest neighboring cells exceeds a specified threshold. Next, we apply Locally Linear Embedding (LLE) to preserve local neighborhood structures, yielding a new *x*-axis represented by the matrix *L* (of dimensions *n*_*l*_ × 2), where *n*_*l*_ denotes the number of selected cells. The LLE algorithm comprises several key steps: neighborhood selection, weight matrix construction, and low-dimensional embedding calculation. For each data point *l*_*i*_ in *L*, its local neighborhood is identified using the k-nearest neighbors method:

$$N(i)=\{{l}_{{j}_{1}},{l}_{{j}_{2}},\ldots,{l}_{{j}_{k}}\}$$

where *N*(*i*) denotes the neighborhood of the data point *l*_*i*_. For each data point *l*_*i*_, we compute weights *p*_*ij*_ that best approximate *l*_*i*_ using its neighbors *N*(*i*), with the objective to minimize the reconstruction error:

$$E(P)=\mathop{\sum }_{i=1}^{n}{\left\Vert {l}_{i}-{\sum}_{j\in N(i)}{p}_{ij}{l}_{j}\right\Vert }^{2}$$

subject to the constraint ∑_*j*∈*N*(*i*)_*p*_*ij*_ = 1, which guarantees that the weights sum to one and maintains local linearity. Once the weights *p*_*ij*_ are determined, the algorithm seeks a low-dimensional embedding *q*_*i*_ for each *l*_*i*_ that preserves the local geometry defined by these weights. The embedding is then optimized by minimizing the following cost function:

$${\Phi }^{{\prime} }(Q)={\sum }_{i=1}^{n}{\left\Vert {q}_{i}-{\sum}_{j\in N(i)}{p}_{ij}{q}_{j}\right\Vert }^{2}$$

After obtaining *Q*, we reorder *L* according to the magnitude of *q*_*i*_ for each element in *L*. To present a smooth result with consistent distances between units on the *x*-axis, we first smooth the points $L{\prime}$ ((*n*_*l*_ − *n*_avg_ + 1) × 2) by averaging the closest *n*_avg_ points:

$${l}_{i}^{{\prime} }=\frac{1}{{n}_{{{{\rm{avg}}}}}}{\sum }_{j=i}^{i+{n}_{{{{\rm{avg}}}}}-1}{l}_{j},\quad i=1,\ldots,({n}_{l}-{n}_{{{{\rm{avg}}}}}+1)$$

Next, we use interpolation functions to create an *x*-axis such that the actual distance between each unit point is consistent. We first calculate the Euclidean distances *d*_*i*_ between two nearby units in $L{\prime}$. The cumulative distance *D*_*i*_ is the total distance from the first point to the *i*-th point. Specifically, *D*_0_ = 0, and for *i* = 1, 2, …, (*n*_*l*_ − *n*_avg_), it is given by:

$${D}_{i}={D}_{i-1}+{d}_{i-1}$$

The total distance ${D}_{{n}_{l}-{n}_{{{{\rm{avg}}}}}}$ represents the distance from the starting point to the last point. A cubic interpolation function *f* is established for the coordinates of $L{\prime}$. We define a new sequence of distances containing *n*_*l*_ − *n*_avg_ points evenly distributed points ${D}_{j}^{{\prime} }$, where:

$${D}_{j}^{{\prime} }=j\cdot \frac{{D}_{{n}_{l}-{n}_{{{{\rm{avg}}}}}}}{{n}_{l}-{n}_{{{{\rm{avg}}}}}},\quad \,{\mbox{for}}\,\quad j=0,1,2,\ldots,{n}_{l}-{n}_{{{{\rm{avg}}}}}$$

The new coordinates $f({D}_{j}^{{\prime} })$ are computed using the interpolation functions for each ${D}_{j}^{{\prime} }$, forming new points *ℓ″*_*j*_. Thus, we create evenly and smoothly distributed points for the *x*-axis.

Depending on the tissue type, the *y*-axis is constructed either based on absolute distances or on cell connectivity. In the first case, for each cell, we identify its nearest unit on the *x*-axis and calculate the Euclidean distance from the cell to that unit, assigning this distance as the y-coordinate. In the second case, cells that are in proximity are considered connected. For each cell, we determine the shortest connected path to the *x*-axis, define the y-layer as the number of cells along that path, and compute the y-coordinate by summing the distances along this path.

However, given the limited computational resources and the vast number of cells, calculating the shortest path for each cell individually is time-consuming. Moreover, the available paths are normally restricted to a limited set of directions. To overcome these challenges, we progressively generate y-layers by iteration. We begin by constructing the first layer using an iterative approach. For each unit on the *x*-axis, we identify the 18 closest cells within a specified distance and label them as Layer 1. Then, for each cell in Layer 1, we find the nearest neighboring cells among the remaining cells within a limited distance. To ensure that the cells are captured in the correct direction, we use a central reference point and require that the direction from the center to the original cell is consistent with the direction from the center to the Layer 1 cell. If a cell is selected multiple times by different Layer 1 cells, we retain the path with the shortest total length and record that path as the shortest one, assigning the corresponding *x*-axis coordinate to that cell. This process continues by iterations: in each iteration, the next layer is generated following the same procedure until no qualified cells are left.

### Mouse brain data analysis from 10x genomics^26^

#### Nuclei segmentation

SMURF requires nuclei segmentation results as input. In this study, we utilized Squidpy (v1.4.1)^89^ with Stardist (v0.8.5)^18^ to perform nuclei segmentation on mouse brain H&E data. We first used SMURF to get the exact spot-occupied area of the H&E plot. Since a small portion of the H&E staining in this data was damaged and exhibited abnormally dark red regions, we converted these areas to a pink hue similar to that of the blank regions. Subsequently, we divided the entire image into 20 blocks with a small overlapping area between adjacent sections. For each block, the 2D_versatile_he model from StarDist was applied, and the segmentation results were then combined. To account for split cells at the block boundaries, we ensured that the overlapping regions were preserved and only retained cells that were completely contained within a block. These refined nuclei segmentation results were used as input for the SMURF analysis of the mouse brain data.

#### SMURF analysis

We input the nuclei segmentation data along with the original output from 10x Genomics and followed the SMURF protocol to generate the final gene expression matrix along with primary clustering results. For the unrolling of the mouse cortex, we first selected the cluster corresponding to the basement membrane of the cortex, then filtered out isolated cells within this cluster and used the filtered cells to build the *x*-axis. We used the Euclidean distance to define the *y*-axis and selected a center point to determine the orientation. Next, we constructed both the *x*-axis and *y*-axis based on the spatial coordinates of the cells. The *y*-axis was evenly divided into six sections, and we subsequently plotted a heatmap of gene expression markers that delineated the distinct layers of the cortex^34,90^.

#### Cell type annotation

We uploaded the complete gene expression dataset directly from the SMURF output to MapMyCells (Allen Institute web tool^35,36^, RRID: SCR_024672) to align cells with the 10X Whole Mouse Brain (CCN20230722) reference^36^. We then selected layer-specific neurons with subclass bootstrapping probabilities greater than 0.6 and plotted these cells along the new axes, with colors indicating layer information. Similarly, we filtered for specific non-neuronal cell types with subclass bootstrapping probabilities exceeding 0.6 and visualized them on the same coordinate system.

### Mouse small intestine data analysis from 10x genomics^27^

#### Nuclei segmentation

SMURF requires nuclei segmentation results as input. In this study, we utilized Squidpy (v1.4.1)^89^ with StarDist (v0.8.5)^18^ to perform nuclei segmentation on mouse small intestine H&E data. We first used SMURF to determine the exact spot-occupied areas of the H&E image. The entire image was then divided into 36 blocks with a small overlapping area between adjacent sections. For each block, the 2D_versatile_he model from StarDist was applied, and the segmentation results were subsequently combined. To account for split cells at block boundaries, we preserved the overlapping regions and retained only those cells that were completely contained within a block. These refined nuclei segmentation results were used as input for the SMURF analysis of the small intestine data.

#### SMURF analysis

We followed the SMURF protocol to obtain the final gene expression matrix along with primary clustering results. For the unrolling analysis, we manually removed regions where the small intestine was inadvertently cut or damaged at both the beginning and the end. The intestine is surrounded by a muscular layer composed of smooth muscle cells and nerves (myenteric plexus). We used this layer to define the *x*-axis position representing the longitudinal axis of the Swiss roll preparation. We identified the clusters representing the muscularis externa and myenteric plexus by their spatial location at the base of the Swiss roll and confirmed their identity by marker-gene expression (e.g., *Acta2*, *Tagln*, and *Myh11* for muscularis externa; *Snap25*, *Uchl1*, and *Prph* for myenteric plexus). We filtered out isolated cells within these clusters. Finally, we adhered to the SMURF pipeline to define the *y*-axis layer by layer. The *y*-axis was assigned using all cells in the tissue except those used to define the *x*-axis, rather than being computed separately for individual villi. The corrected *y*-axis is first obtained in physical units (*μ*m), and then we calculate a normalized corrected *y*-axis by setting the tip of each villus to a fixed value of 20 (Supplementary Fig. 10c; Supplementary Fig. 11). This facilitates comparison across villi of different lengths and shapes.

#### Single-cell downstream analysis

We followed the Scanpy (v. 1.10.0)^19^ pipeline to analyze the raw counts generated by SMURF. For Peyer’s patch-related clusters, the minimum total-count threshold was set to the smaller of 50 and the 5th percentile of per-cell total counts within that cluster, whereas for all other clusters, the minimum total-count threshold was set to the smaller of 50 and the 10th percentile of per-cell total counts within that cluster. Cells with total counts less than or equal to the corresponding cluster-specific threshold were removed. We then applied global quality-control filters, excluding cells with 2000 or more detected genes, 2000 or more total counts, a mitochondrial gene fraction of 25% or higher, or a cell size of 45 or greater. These quality-control thresholds were used to remove low-quality or abnormal cells before downstream analysis. After quality control filtering, 375,999 of 404,382 reconstructed cells were retained for downstream analysis and annotation, whereas 28,383 cells (7.0%) were removed because of low-count and/or poor-quality metrics.

We then normalized the cells by scaling each cell’s total counts to 1000 and performed log-normalization to stabilize variance across cells. Next, we retained the top 4000 variable genes and two key genes (*Pyy* and *Sct*). The data were scaled such that the maximum expression value for each gene was set to 10. We performed PCA to reduce the dimensionality to 40 components, followed by the construction of a nearest-neighbor graph using 25 neighbors. For visualization, we applied UMAP, and cell clustering was conducted using the Leiden algorithm with a resolution of 3^21,91^. Differential expression analysis was carried out using the Scanpy (scanpy.tl.rank_genes_groups, wilcoxon)^19^. We annotated cell types using marker genes according to reference data and knowledge with the help of location information^31,92,93^.

To detect the crypt-villus boundary, we initially split each crypt-villus unit into a lower and upper section at normalized *y*-axis values of 9. Cells with normalized *y*-axis values less than 9 were assigned to the crypt group, and those with higher values were assigned to the villus group. We then used a classifier and mixture-based refinement applied to each crypt-villus unit individually to refine the exact transition point at that site. Briefly, we conducted differential gene expression analysis between these two groups, selecting genes with an adjusted *P* value below 1 × 10^−5^. Using these genes, we built a tree-based classifier using XGBoost (v2.1.1)^94^ to distinguish between crypt and villus cells based on their expression profiles. Recognizing that the crypt-villus continuum is a continuous structure, we further examined the local neighborhood of each cell. We assumed that the distribution of normalized *y*-axis positions for the crypt and villus groups follows a mixed Gaussian distribution and used the resulting boundary to precisely define the crypt-villus interface at each location (Supplementary Fig. 26). The dashed line in Fig. 3l is calculated as the average of crypt-villus boundary.

#### Pseudotime analysis

We used Scanpy (v1.10.0)^19^ to perform pseudotime analysis on a subset of epithelial cells spanning the crypt-base to villus axis, including the combined Paneth/stem group, TA cells, and enterocytes. This subset contained 139,637 cells in total. First, we computed diffusion maps to capture the major continuum of epithelial state variation. A putative starting cell was selected from the crypt-base Paneth/stem group, and pseudotime was computed using the diffusion pseudotime function (sc.tl.dpt)^53^. To better visualize the structure of this cell subset, we employed PAGA (sc.tl.paga)^54^ and force-directed graph drawing (sc.tl.draw_graph)^55^ to generate a layout on which the pseudotime values were mapped.

We then normalized both the pseudotime values and the normalized corrected *y*-axis to a scale from 0 to 1. Using pseudotime (0-1) as the independent variable and the normalized corrected *y*-axis (0-1) as the dependent variable, we performed linear regression analysis and computed the Pearson correlation coefficient to assess the strength and significance of their relationship. The regression analysis was performed on the same 139,637 cells used for pseudotime analysis.

#### Zonation analysis

We selected only complete villi for zonation analysis. We used the presence of tip enterocytes as a marker of villus completeness. To select intact villi in an unbiased manner, the intestine was divided into small sections along the *x*-axis. We applied a Kolmogorov–Smirnov test to evaluate the distribution of tip enterocytes in each section against a uniform distribution. To ensure comprehensive coverage, multiple candidate sections from each area that passed the test were retained. We refined the final list of villi by excluding a small number of sections with obvious artifacts on manual review of the corresponding H&E image. This procedure retained over 100 intact villus structures for downstream zonation analysis. We then calculated an *x*-axis anchor position for each of the intact discrete villi. Epithelial cells nearest those anchors were assigned to that villus, and the previously calculated *y*-axis coordinates were used to assign each cell a position on that crypt-villus axis. We retained Paneth and stem cells, TA cells, and enterocytes. Cells were then subdivided into six distinct zones according to their normalized corrected *y*-axis, with approximately equal numbers of cells per zone. Only genes with counts greater than 10 were retained (12,332 genes in total). For visualization, the average expression of each gene across zones was normalized to a maximum of 1. To identify the genes that were significantly zone-associated, we used pairwise differential expression analysis of each zone against all other zones using Scanpy (scanpy.tl.rank_genes_groups, wilcoxon)^19^. We considered a gene as significantly zone-associated if its absolute ${\log }_{2}$ fold-change (LFC) was greater than 1.5 and the adjusted *P* value was below 1 × 10^−8^ compared to all other zones.

#### KEGG analysis

We used gprofiler-official (v1.0.0)^95^ to analyze KEGG pathway enrichment for each zone. KEGG pathways were retained if any zone exhibited an adjusted *P* value below 1 × 10^−8^ and an LFC above 1.5. We generated bubble plots of significant KEGG pathways across all zones, where the bubble size indicates the fraction of genes from that pathway that were significantly enriched in the zone, and the bubble color represents the corresponding *P* value.

#### Gene expression by zone

We used the *y*-axis boundaries identified from intact villi in the zonation analysis for all plots of cell-type-specific gene expression by zone. Cells of the designated cell type were assigned to zones according to their normalized corrected *y*-axis positions relative to these predefined boundaries. For each cell type and zone, expression was summarized as the mean log-normalized expression across cells in that zone. Zone-specific values were displayed only when more than 50 cells of that lineage were present in the corresponding analysis set. For visualization, 95% bootstrap confidence intervals for the mean were calculated by resampling cells within each zone with replacement (500 iterations). These intervals reflect within-section cell-level variability from one mouse intestinal section and should not be interpreted as estimates of between-villus or between-mouse variation.

We then sought to capture proximal-distal differential genes across different zones. Cells were first divided along the *x* − axis, with the first half designated as the proximal group and the second half as the distal group. Only Paneth and stem cells, TA cells, and enterocytes were included. Cells were then assigned to crypt-villus zones as described above, with approximately equal numbers of cells per zone. Within each zone, mitochondrial genes were excluded, and only the genes expressed in at least 100 cells were retained. Differential-expression analysis was then performed by comparing proximal and distal cells within each zone. Genes were retained if the absolute ${\log }_{2}$ fold change exceeded 1.5 and the adjusted *P* value was below 0.001. The number of differential genes in each zone was normalized by the number of genes remaining after filtration. Finally, we generated plots of the differential gene count and the fraction of differential genes versus zones. KEGG pathways of the proximal and distal zone 6 genes were calculated if they exhibited an adjusted *P* value below 0.001 and an LFC above 1.5. Line-plot trajectories were drawn for important marker genes across the zonation axis. Because stem, Paneth, and TA cells are predominantly confined to Zones 1-2, we displayed these three populations together with enterocytes for direct comparison. For every other lineage, the plot was restricted to cells of that specific type.

#### Transcription factor analysis

We selected Paneth cells, stem cells, TA cells, and enterocytes - each annotated with villus-zone information — from the previous section, and then followed the pySCENIC protocol (https://github.com/aertslab/SCENICprotocol) by applying pyscenic (v0.12.1+6.g31d51a1) to infer TF–target gene regulatory networks and to identify and score regulons significantly enriched in our dataset by comparison with established reference databases^64^.

A total of 241 regulons have been called by the results. We selected zone-specific regulons (LFC  > 0.3 and adjusted *p*-value  < 1  × 10^−10^) and plotted the TF-target network for all six zones. For Zone 2, we built a TF-target network for the seven regulons whose activities differed significantly across zones by differential t-test from Scanpy. In the graph, large blue nodes denote the TF genes, small green nodes mark non-zonated targets, red nodes mark zonated targets, and nodes in additional colors highlight zonated targets enriched for specific functional categories. To probe zonation enrichment, we applied Fisher’s exact tests to compare genes targeted by at least two TFs with all remaining targets and to compare each TF with its unique targets with the pool of all other target genes separately. We completed the same analysis for Zone 6 with five differential regulons.

#### Single-cell analysis of Seiler et al. ^77^

We downloaded the dataset from GEO (GSE130113), which contains raw counts from three sham and two small bowel resection replicates. We first filtered cells by requiring a minimum of 200 genes per cell and filtered genes by retaining only those expressed in at least 3 cells. The data were then normalized to a total of 10,000 counts per cell and log-normalized. We selected highly variable genes, scaled the data, performed PCA, and applied Harmony for batch correction. UMAP was used for dimensionality reduction, and clustering was performed using the Leiden algorithm^21,91^. Differential expression analysis was carried out using a t-test. Although the differential results were not identical to those reported in the original paper, they were extremely similar—the top five genes were the same, and all the top 10 genes reported in the paper were within the top 11 genes in our analysis. Finally, we selected differential genes using a threshold of adjusted *P* value less than 1 × 10^−50^ and an absolute LFC larger than 1, resulting in a total of 77 genes significantly differentially expressed in the small bowel resected (SBR) group.

We then mapped the differential genes to the spatial dataset, 72 of which were detected in the spatial data. Next, we assigned zone-associated genes by defining a gene as associated with a given zone if its ${\log }_{2}$ fold change in that zone (compared to the average expression in all other zones) was greater than 1.5 and its adjusted *P* value was less than 1 × 10^−8^. To test whether these SBR-associated genes were preferentially distributed across spatial zones, we used a permutation-based enrichment test. Specifically, we compared the observed overlap between the SBR-associated gene set and the zone-associated gene set in each spatial zone with the overlap expected under random sampling from the background gene universe. Statistical significance was evaluated empirically by repeatedly sampling random gene sets of the same size as the SBR-associated gene set from the background universe without replacement and recalculating the enrichment statistic for each random sample (100,000 permutations). Finally, we plotted the spatial zones to which the SBR-associated genes were assigned in the spatial dataset.

### Projecting cells across different tissues and platforms

#### Mouse olfactory bulb—Stereo-seq Dataset^2^

We cropped one hemibulb and selected the boundary part of the hemibulb as the unfolding reference for the *x*-axis. The y-axis was computed as Euclidean distances to the ONL in two segments and then merged as above. We selected a contiguous region bounded by isocontours and visualized spatial ordering with violin plots of *y*-axis distributions across major cell types.

#### Human frontal cortex—NanoString CosMx Dataset^49^

We directly used the cell-level segmentation, quality control, and annotation results as input. We treated the astro.1 boundary cluster as the reference trajectory for the *x*-axis. For each cell, *y*-axis was defined as the Euclidean distance to the nearest point on this axis. To accommodate local curvature, the tissue was partitioned into two segments; distances were computed within each segment and then concatenated to preserve a consistent origin and units. We used MapMyCells (Allen Institute web tool^35,36^, RRID: SCR_024672) to annotate the cells. We then selected a contiguous region bounded by density isocontours and summarized layer-specific organization using violin plots of the *y*-axis distributions for cortical cell types.

#### Mouse retina—MERFISH Dataset^48^

We analyzed one of the retinas from sample VZG105a_WT2. Retinal ganglion cells were used to define the *x*-axis for tissue unrolling. For all other cells, *y*-axis positions were computed as the Euclidean distance to the RGC axis, and cells at the lateral margins were removed. Because retinal cell types are arranged in layers, we summarized *y*-axis distributions by cell type and marker genes using violin plots.

#### Human skin melanoma—10x Xenium Prime Dataset^50^

We used the clustering results from the 10x Genomics website. Cluster 28 defined the *x*-axis reference, and *y*-axis was computed as Euclidean distances to this axis. We generated violin plots for the four principal epidermal clusters. In a complementary unrolling, the basal layer (cluster 20) was set as the origin (*y* = 0), with epidermal layers mapped to *y*≤0 and extra-epidermal areas to *y* > 0.

### Comparison analysis for results from different methods

We compared the results of 8 μm spot data, Bin2cell results, nuclei segmentation results, Space Ranger results, and SMURF results. For each dataset, we used the direct output of 4 × 4 spots (corresponding to an 8 × 8 μm spot size) as the 8 μm spot data. For Bin2cell, we used Bin2cell (v0.2.0) following the instructions provided in the Bin2cell GitHub repository^96^. Nuclei segmentation results were obtained using Squidpy (v1.4.1)^89^ with StarDist (v0.8.5)^18^. For Space Ranger, we used 10x Genomics Space Ranger (v4.0.1) to generate cell segmentation and transcript assignment results according to the manufacturer’s recommended Visium HD workflow^97,98^. For benchmarking against morphological expansion, we used Spateo (v1.1.1) to generate expanded regions from nuclei masks^2^.

In the analysis of the percentage of cells covered by nuclei, we encountered the issue that a single spot might encompass several nuclei, leading to multiple nuclei being assigned to a cell, an outcome that is not accurate. To address this, we defined a cell as covered by a nucleus only if at least 30% of the pixels in the cell region are occupied by that nucleus. For analyses performed on the entire image, we did not apply any additional filtering to spots or cells. However, as part of the standard 10x alignment pipeline, some 8 μm spots with zero counts were automatically excluded. For analyses restricted to image regions containing nuclei, we retained only those spots or cells with at least one pixel overlapping a nucleus segmentation mask.

For the definition of nuclei data in the Mouse Brain dataset, we considered a spot to belong to a nucleus if at least 90% of the spot’s counts were within that nucleus. We then uploaded the raw counts matrix of nuclei, SMURF, and Xenium results from 10x Genomics^28^ to MapMyCells^35,36^ by selecting the 10x Whole Mouse Brain (CCN20230722) as the reference dataset. Using the Hierarchical Mapping strategy available in MapMyCells, we obtained the cell type results for the three datasets. We calculated the KL divergence according to the class_name from the MapMyCells results. In the mouse small intestine dataset, we applied the same segmentation and clustering pipeline as provided by 10x Genomics^99^.

### Statistics and reproducibility

Exact sample sizes for each analysis are provided in the corresponding figure legends and refer to the number of cells, genes, or tissue structures analyzed, as specified. Unless otherwise stated, violin plots summarize single-cell distributions and are descriptive. Ileal zonation analyses were performed on one mouse intestinal section. This section contained over 1000 villi and 100 intact villi. For enterocyte zonation analyses, cells were assigned to crypt-villus zones according to the normalized corrected *y*-axis, and differential-expression statistics were computed by comparing cells from each zone with cells from all other zones. For lineage-specific zonation plots, expression was summarized at the cell level within each zone, and shaded bands indicate 95% bootstrap confidence intervals obtained by resampling cells within zones with replacement (500 iterations).

### Computational resources

All analyses were executed on a single Ubuntu 20.04.6 LTS workstation equipped with an Intel^®^ Core™ i9–10920X CPU (12 cores/24 threads), 251 GiB RAM, and an NVIDIA GeForce RTX 3090 GPU (24 GiB VRAM). Storage requirements depend on the dataset and analysis settings, but in our experience, SMURF can analyze a mouse brain dataset with 25 GB of available storage, whereas larger datasets or analyses that retain high-resolution visualizations and intermediate files may require on the order of 100 GB.

### Reporting summary

Further information on research design is available in the Nature Portfolio Reporting Summary linked to this article.

## Supplementary information

Supplementary Information

Description of Additional Supplementary Files

Supplementary Data 1

Reporting Summary

Transparent Peer Review file

## Data Availability

The public mouse brain Visium HD and mouse small intestine Visium HD datasets are available on the 10x Genomics website (mouse brain Visium HD, mouse intestine Visium HD). Public mouse brain 10x Xenium data can be accessed on the 10x Genomics website (fresh-frozen mouse brain replicates). Public Mouse retina MERFISH data (sample ID VZG105a_WT2) is from Choi et al. (2023)^48^ and is available via Zenodo (Zenodo record). The public adult mouse olfactory bulb Stereo-seq dataset is from Chen et al. (2022)^2^ and can be downloaded from the STOmics/MOSTA portal (MOSTA download(Mouse_olfa_S2.h5ad)). The public human frontal cortex NanoString CosMx SMI dataset can be accessed on the NanoString website (Human frontal cortex CosMx)^49^. The public human skin melanoma 10x Xenium Prime dataset is available on the 10x Genomics website (Human Skin Melanoma 10x Xenium Prime)^50^. The public mouse liver Seq-Scope dataset was originally generated by Cho et al. (2021)^100^ and was obtained from Chen et al. (2023)^8^. The public SBR experiment dataset from Seiler et al. (2019)^77^ is available from GEO under accession number GSE130113. The ISH staining for gene *Slc1a2* was obtained from the Allen Brain Atlas (https://mouse.brain-map.org/gene/show/20273). Additional public datasets used in this study included the 10x Genomics Visium HD mouse embryo and human tonsil fresh-frozen IF datasets for SMURF-lite benchmarking^101,102^, and a published mouse intestine CODEX dataset for exploratory analyses of SMURF unrolling on protein-based spatial data^78^. No new data were generated.

## Code availability

The SMURF package is available at https://github.com/The-Mitra-Lab/SMURF. The code to reproduce the results presented in this study can be accessed at https://github.com/The-Mitra-Lab/SMURF_figures and archived on Zenodo^103^. The documentation for SMURF is available at https://the-mitra-lab.github.io/SMURF/.
