# Supplementary material for: SMURF: soft-segmentation for single-cell reconstruction and topological analysis of spatial transcriptomic data
Source: Nat Commun. 2026 Jun 26;17:7990. doi: 10.1038/s41467-026-74464-4 (PMC13447825; doi:10.1038/s41467-026-74464-4)
Supplement: Supplementary file 1 [file 41467_2026_74464_MOESM1_ESM.pdf]

SMURF: soft-segmentation for single-cell reconstruction and topological analysis of spatial transcriptomic data - Supplementary Note

Contents

Supplementary Figures **2**

Supplementary Tables **29**

Supplementary Notes **36**

References **59**

Supplementary Figures

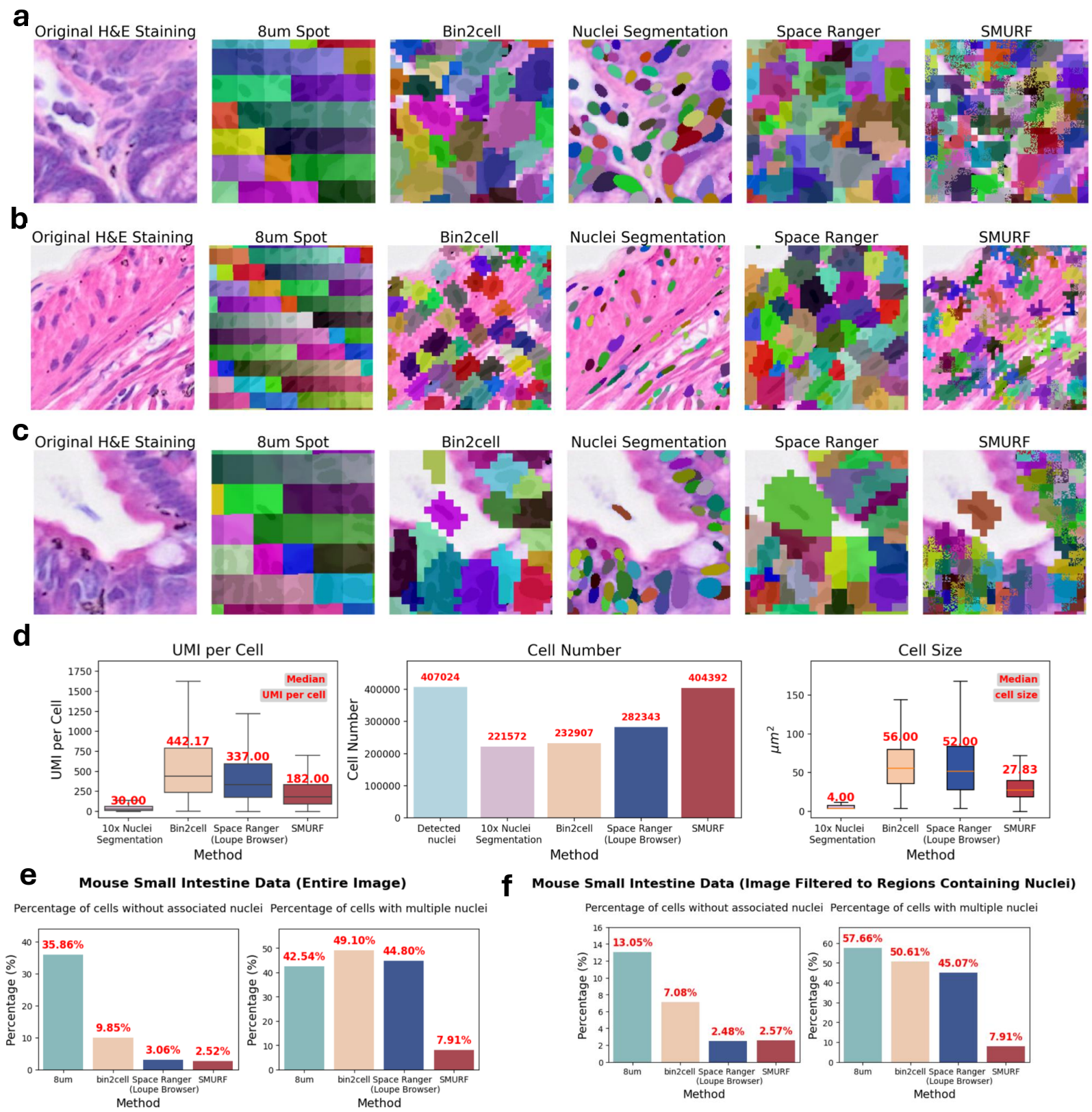

Supplementary Figure 1: **Extended comparison of Visium HD segmentation methods on the mouse small intestine dataset.** (a-c) Representative image patches from three mouse small intestine regions showing how different ST segmentation methods aggregate Visium HD capture spots to define cells. Columns show the original H&E image, 8 μm spots, and the corresponding cell assignments produced by Bin2cell, nuclei segmentation, Space Ranger, and SMURF. Capture spots are colored according to their assigned cell identity. (d) Number of UMIs assigned per cell, total cells detected, and cell size for each method. Numbers above box plots indicate median values, whereas numbers above bars indicate total numbers of detected cells. Box plots show the median, interquartile range and 1.5$\times$ interquartile range whiskers; outliers beyond whiskers are not shown. No biological replicate comparison was performed. (e) Percentage of cells without an associated nucleus and percentage of cells containing multiple nuclei, calculated over the entire image. (f) Percentage of cells without an associated nucleus and percentage of cells containing multiple nuclei, calculated after restricting the analysis to regions containing nuclei. Abbreviations: ST, spatial transcriptomics; UMI, Unique Molecular Identifier.

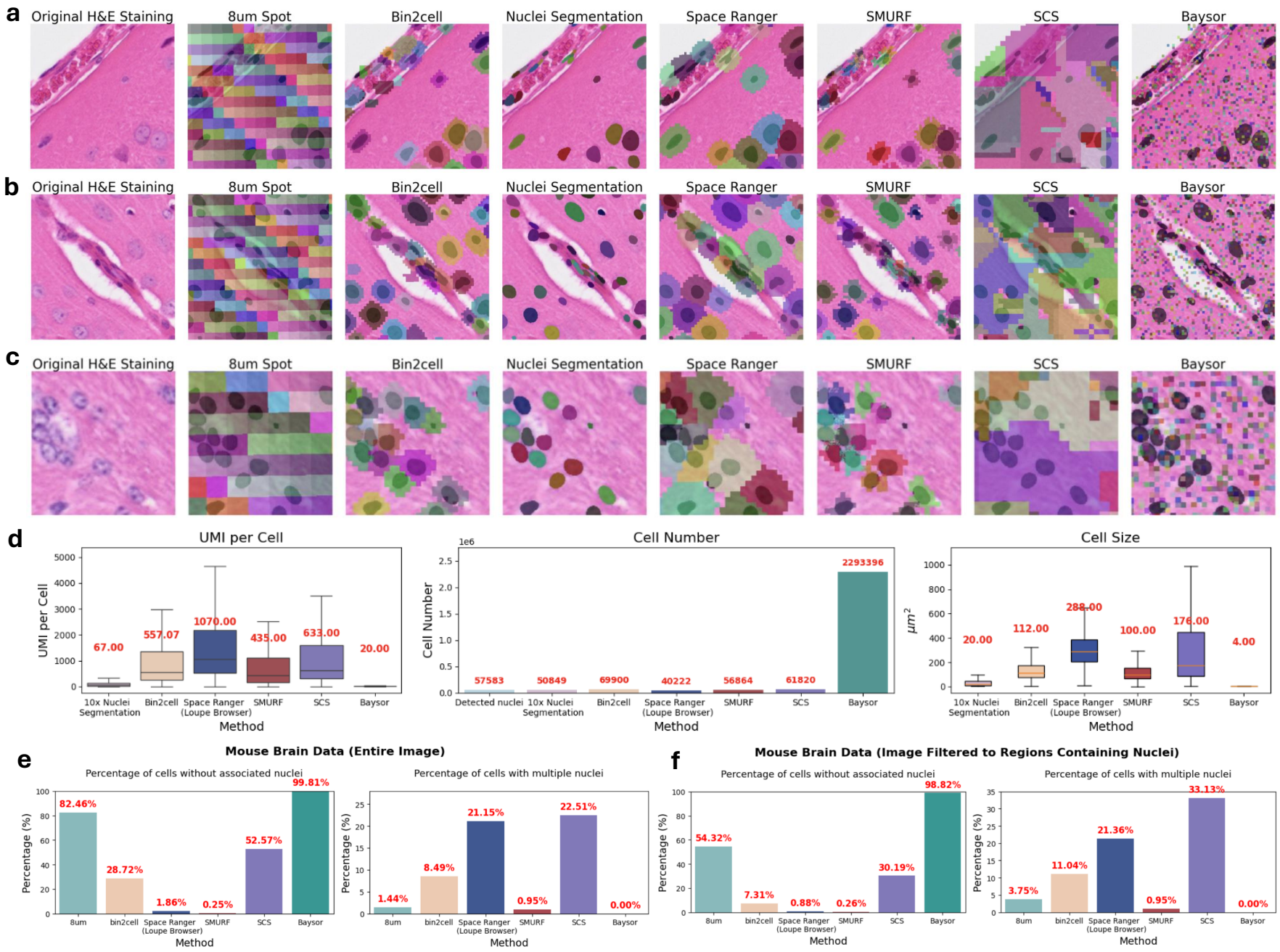

Supplementary Figure 2: **Extended comparison of ST segmentation methods on the mouse brain dataset.** (a-c) Representative images illustrating how different ST segmentation methods aggregate Visium HD capture spots to define cells in mouse brain tissue. Columns show the original H&E staining, 8 μm spots, and cell assignments from Bin2cell, nuclei segmentation, Space Ranger, SMURF, SCS, and Baysor. Capture spots are shaded according to their assigned cell. (d) Number of UMIs assigned per cell, total cells detected, and cell size for each method. Numbers above box plots indicate median values, whereas numbers above bars indicate total numbers of detected cells. Box plots show the median, interquartile range and 1.5× interquartile range whiskers; outliers beyond whiskers are not shown. No biological replicate comparison was performed. (e,f) Percentage of cells without an associated nucleus and percentage of cells containing multiple nuclei, calculated using the entire image (e) or only regions containing nuclei (f). Abbreviations: ST, spatial transcriptomics; UMI, Unique Molecular Identifier.

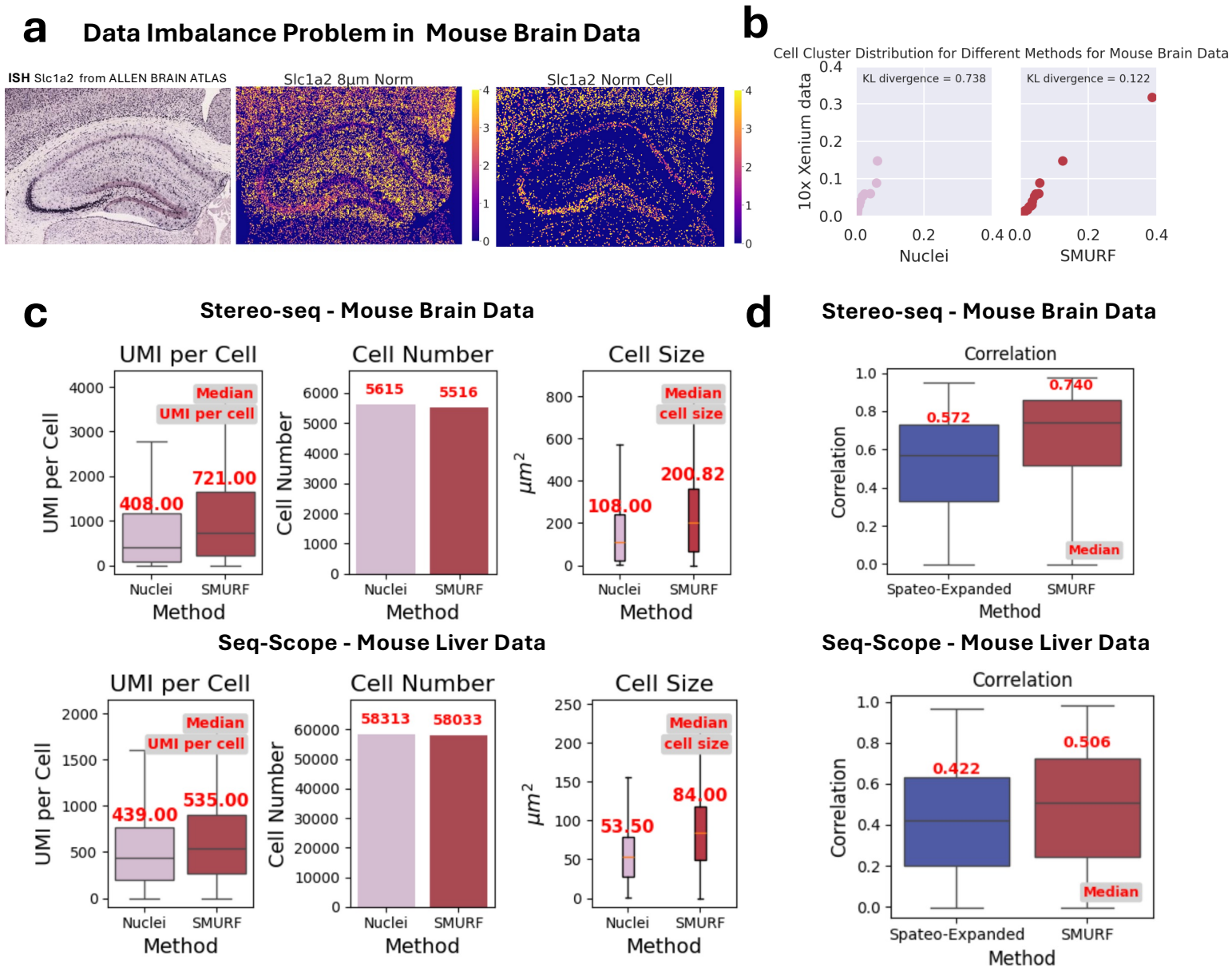

Supplementary Figure 3: **Soft segmentation with SMURF** (a) Illustration of the data imbalance issue when the standard normalized 8 μm Spot method is used. The spatial expression pattern of *Slc1a2* is discordant between Visium HD mouse brain data analyzed by the 8 μm Spot method and ISH data from the Allen Brain Atlas. In contrast, when analyzed by SMURF, there is concordance. (b) Cell type proportions annotated by MapMyCells in probe-based mouse brain data, comparing nuclei results and SMURF results. KL divergence values quantify differences in cell type distributions. Lower KL divergence indicates better concordance. (c) Median number of UMIs assigned to cells, total cells detected, and median cell size using SMURF or nuclei-based segmentation. Numbers above box plots indicate median values, whereas numbers above bars indicate total numbers of detected cells. Box plots show the median, interquartile range and 1.5× interquartile range whiskers; outliers beyond whiskers are not shown. No biological replicate comparison was performed. (d) Per-cell UMI correlation between nuclei segmentation and Spateo/S-MURF expansion.

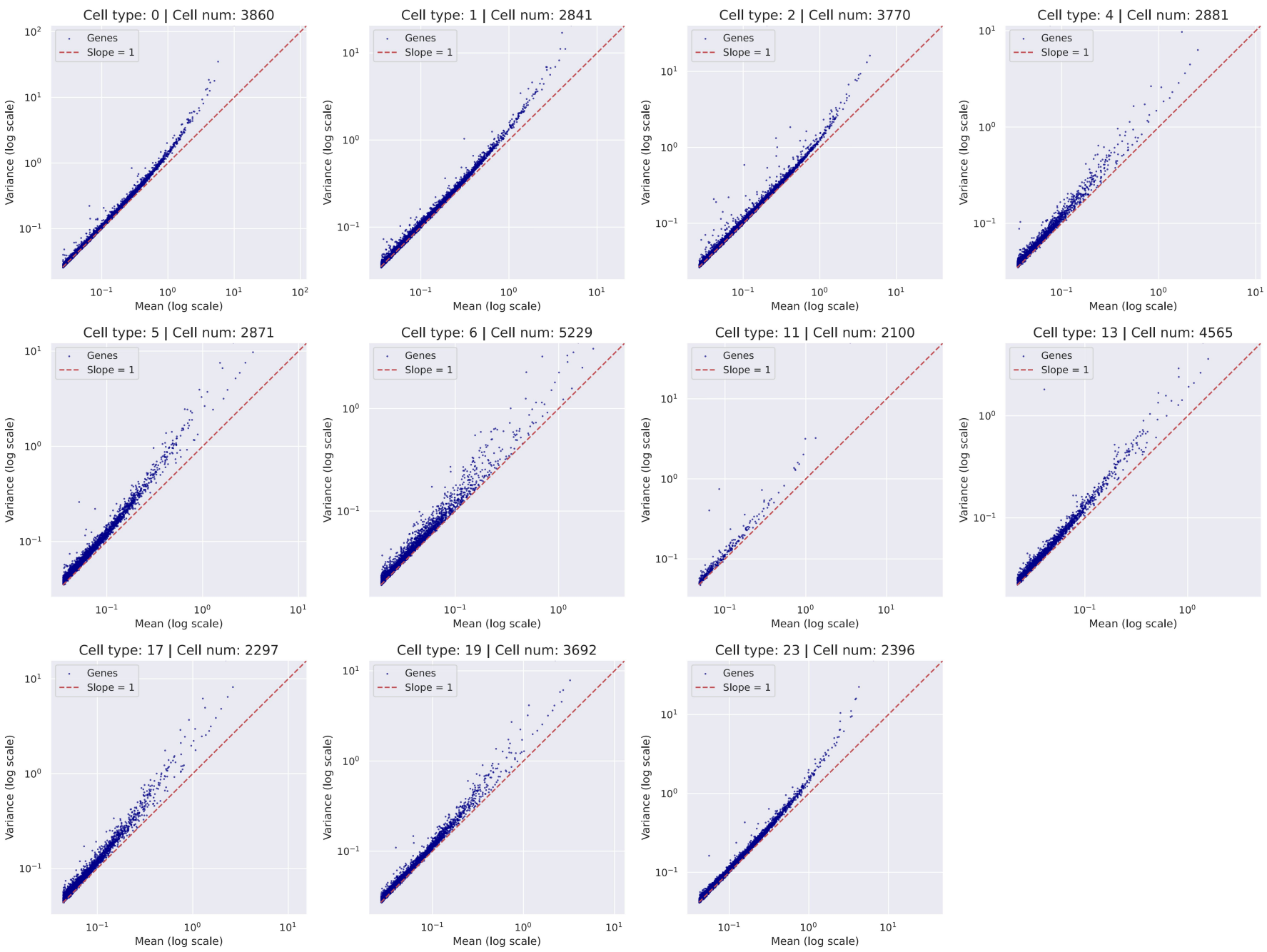

Supplementary Figure 4: **Mean–variance relationship across genes in SMURF-derived single-cell profiles from the mouse brain dataset.** Each panel corresponds to one cell cluster with more than 1,000 cells. Each dot represents one gene with more than 100 total counts across cells in that cell cluster. The x-axis shows the mean expression, and the y-axis shows the variance, both on a log scale. The red dashed line indicates the identity line (variance = mean), corresponding to the Poisson expectation. Across cell clusters, many genes lie above the identity line, indicating overdispersion relative to a Poisson model.

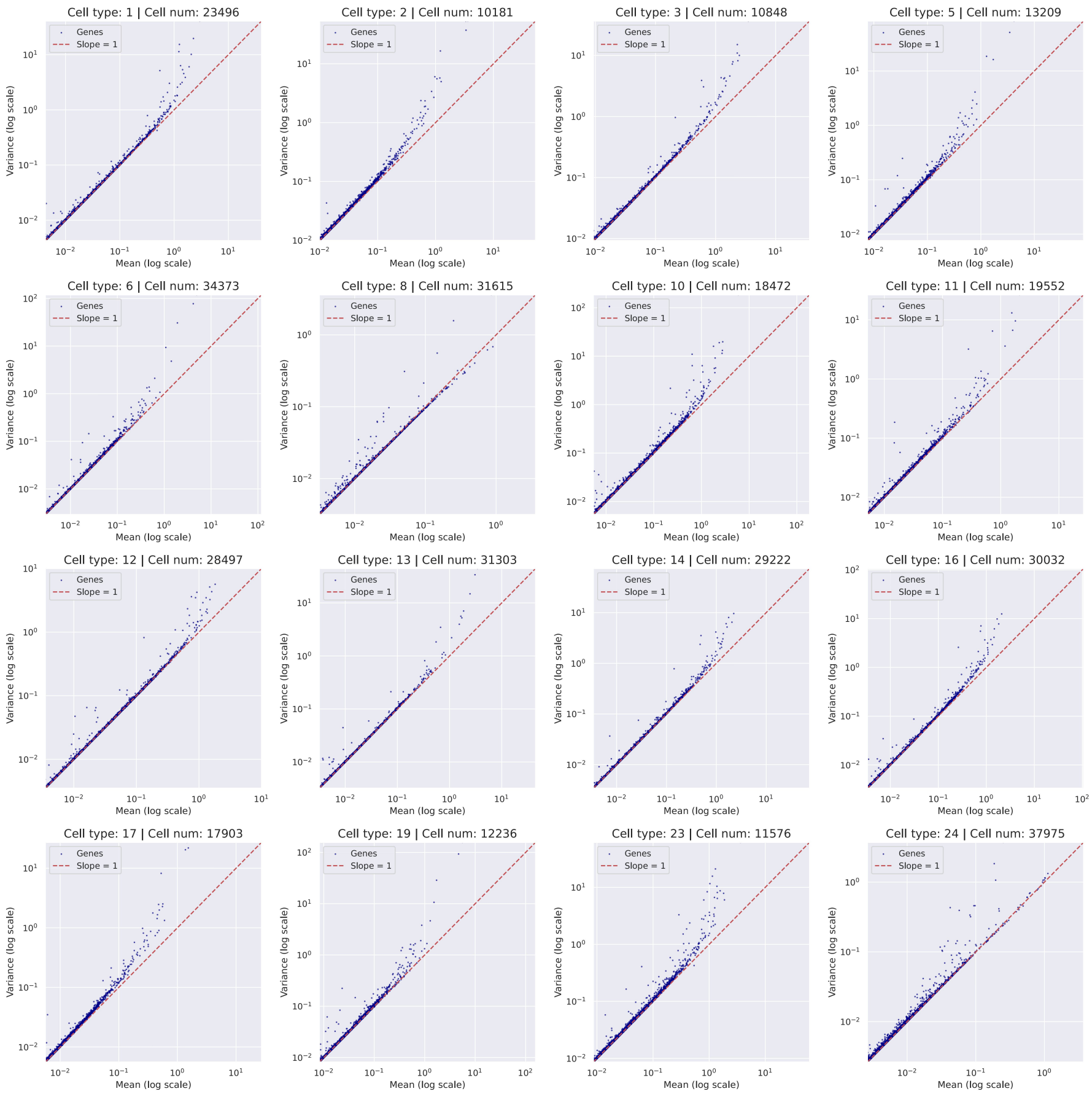

Supplementary Figure 5: **Mean–variance relationship across genes in SMURF-derived single-cell profiles from the mouse small intestine dataset.** Each panel corresponds to one cell cluster with more than 10,000 cells. Each dot represents one gene with more than 100 total counts across cells in that cell cluster. The x-axis shows the mean expression and the y-axis shows the variance, both on a log scale. The red dashed line indicates the identity line (variance = mean), corresponding to the Poisson expectation. Across cell clusters, the variance generally exceeds the mean, consistent with overdispersion in the reconstructed spatial single-cell data.

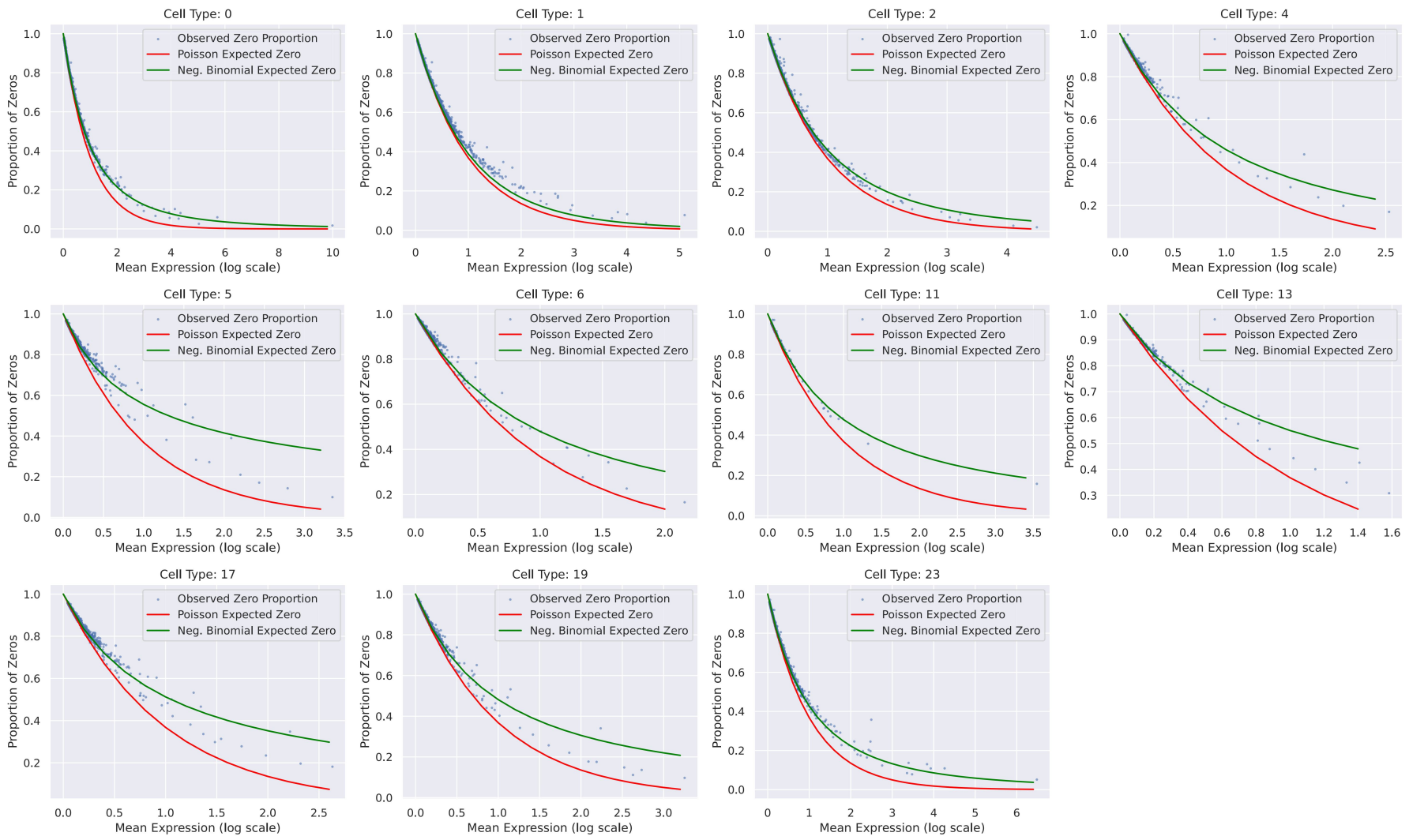

Supplementary Figure 6: **Observed and expected zero proportions across genes in single-cell profiles from the mouse brain dataset.** Each panel shows one cell cluster. Each blue dot represents one gene, plotted by its mean expression (x-axis, log scale) and observed proportion of zero counts across cells (y-axis). The red curve shows the expected zero proportion under a Poisson model, and the green curve shows the expected zero proportion under a negative binomial model with gene-wise dispersion. For most cell clusters, the observed zero proportions are substantially higher than Poisson expectations and are more closely matched by the negative binomial model.

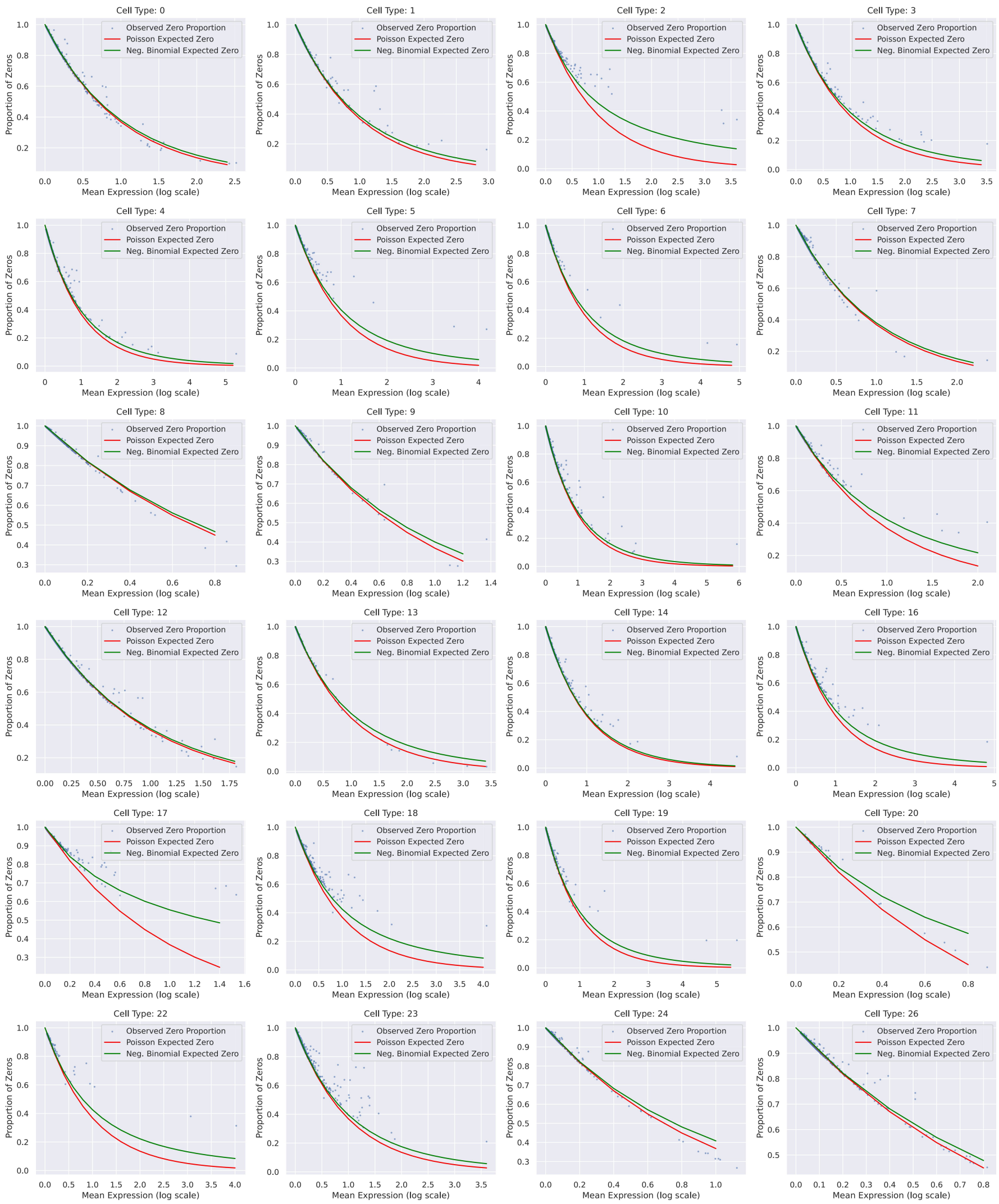

Supplementary Figure 7: **Observed and expected zero proportions across genes in single-cell profiles from the mouse small intestine dataset.** Each panel shows one cell cluster. Each blue dot represents one gene, plotted by its mean expression (x-axis, log scale) and observed proportion of zero counts across cells (y-axis). The red curve shows the expected zero proportion under a Poisson model, and the green curve shows the expected zero proportion under a negative binomial model with gene-wise dispersion. Across cell clusters, the negative binomial model generally provides a closer match to the observed zero proportions than the Poisson model.

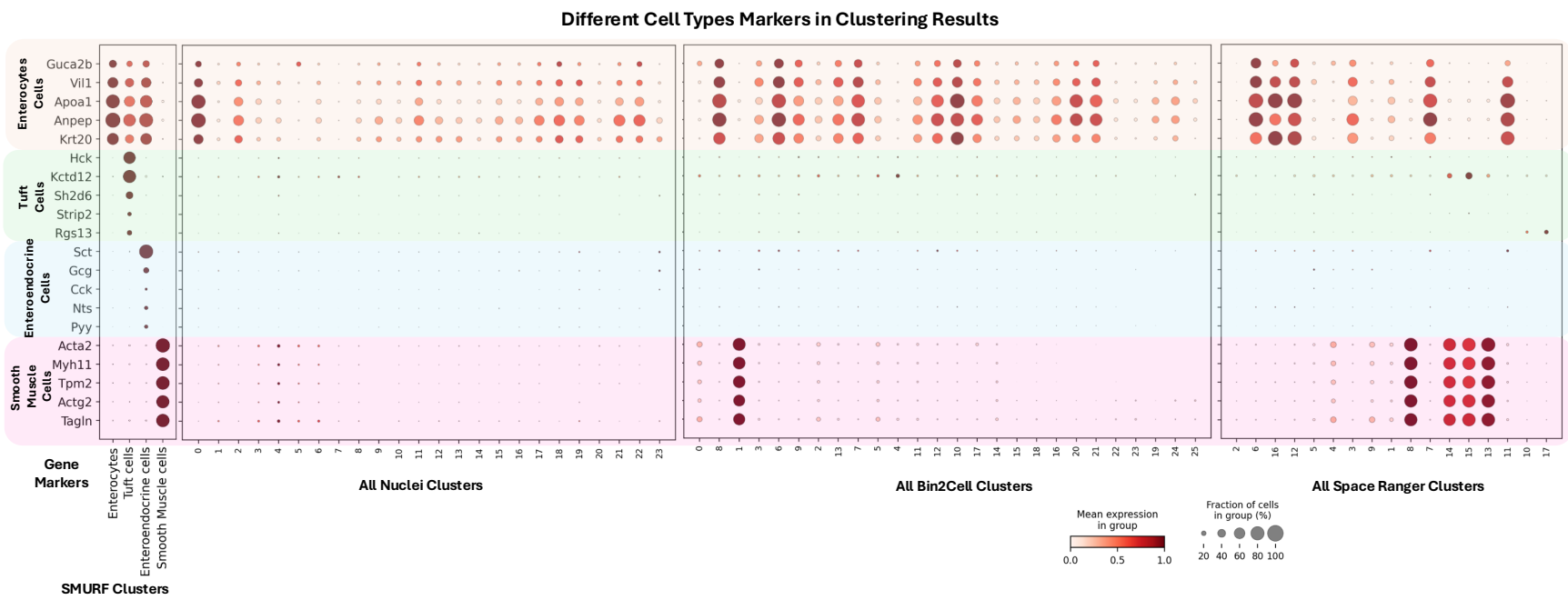

Supplementary Figure 8: **Marker-based comparison of clustering results across transcript-assignment methods in the mouse small intestine dataset.** Rows show canonical marker genes for enterocytes, tuft cells, enteroendocrine cells, and smooth muscle cells. Columns show clusters identified from SMURF, nuclei segmentation, Bin2cell, and Space Ranger outputs. Rare epithelial marker signatures, particularly for tuft cells and enteroendocrine cells, were weak or not cleanly resolved across these alternative methods.

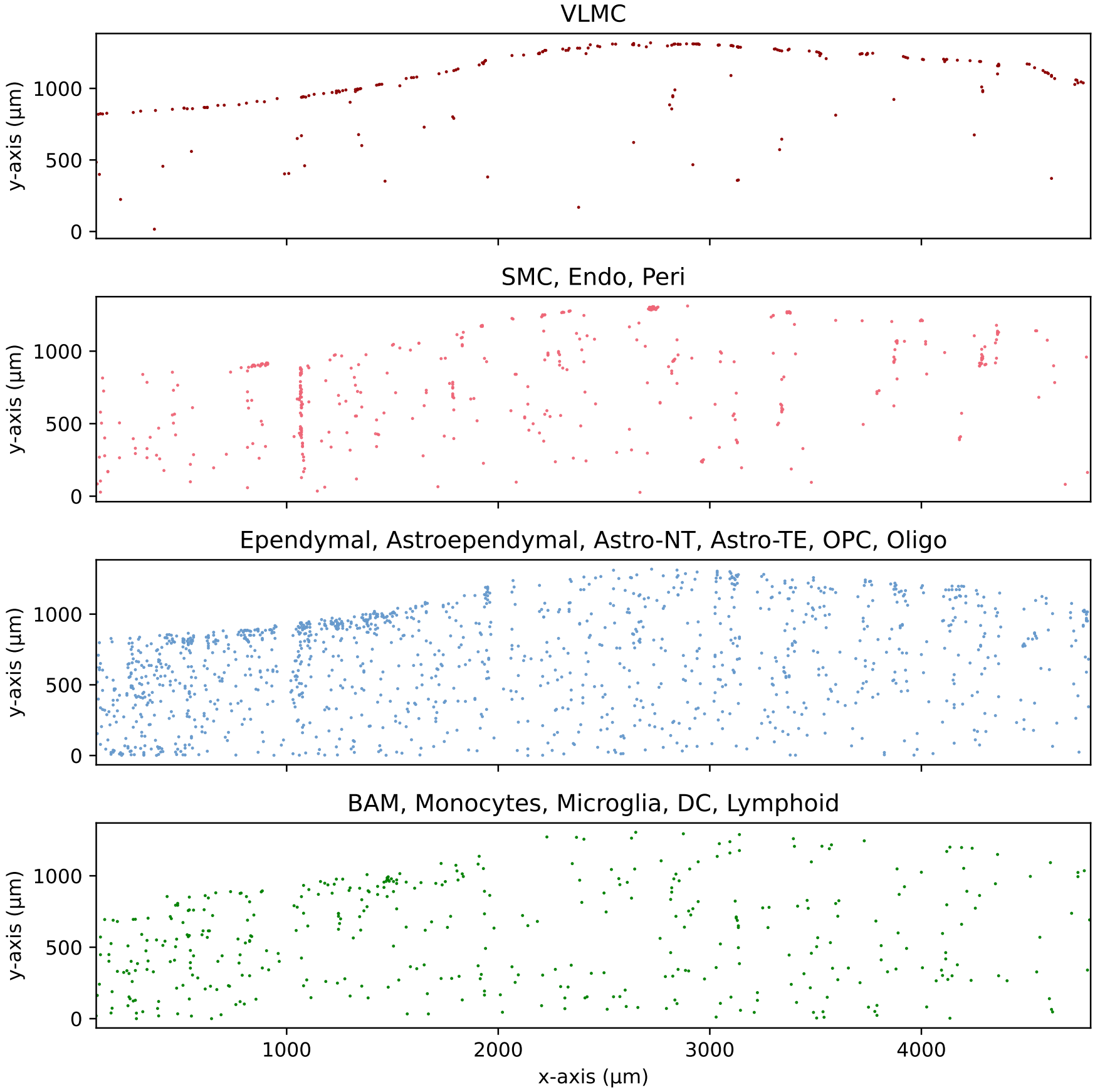

Supplementary Figure 9: **Distribution of cortical layer-specific non-neuronal cells in the unrolled rectilinear coordinate system** Cell segmentation and annotation of mouse cortical cells as in main text Figure 2.

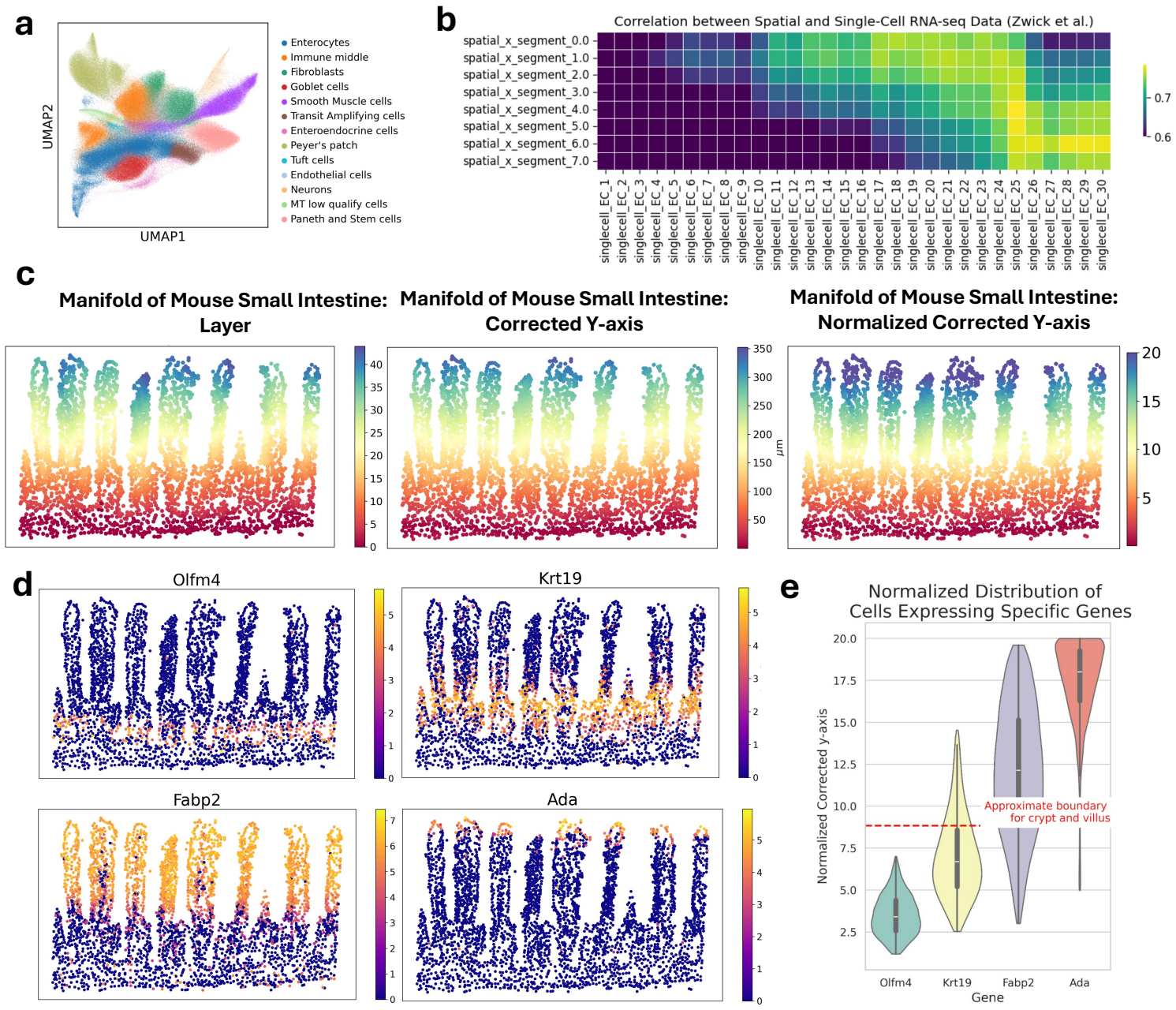

Supplementary Figure 10: **Mouse small intestine analysis.** (a) UMAP embedding of intestinal cells, colored by annotated cell types. (b) Correlation between single-cell transcriptomes from Zwick et al. [1] and spatial transcriptomic profiles across intestinal sections. (c) Overlay of selected mouse small intestine data with cells colored by coordinate value along the y-axis at different stages of "unrolling": raw layer positioning, corrected Y (μm), and normalized corrected Y. (d) Marker gene expression visualized in the selected unrolled intestinal tissue (*Olfm4*, *Krt19*, *Fabp2*, and *Ada*). (e) Normalized corrected y-axis distribution of cells expressing selected genes with red dashed lines indicating the approximate boundary between crypt and villus regions. Each violin shows the normalized corrected y-axis positions of individual cells expressing the indicated gene; widths indicate cell density. The overlaid box plot shows the median, first and third quartiles, and whiskers extending to the most extreme values within 1.5× the interquartile range; outliers beyond whiskers are not shown.

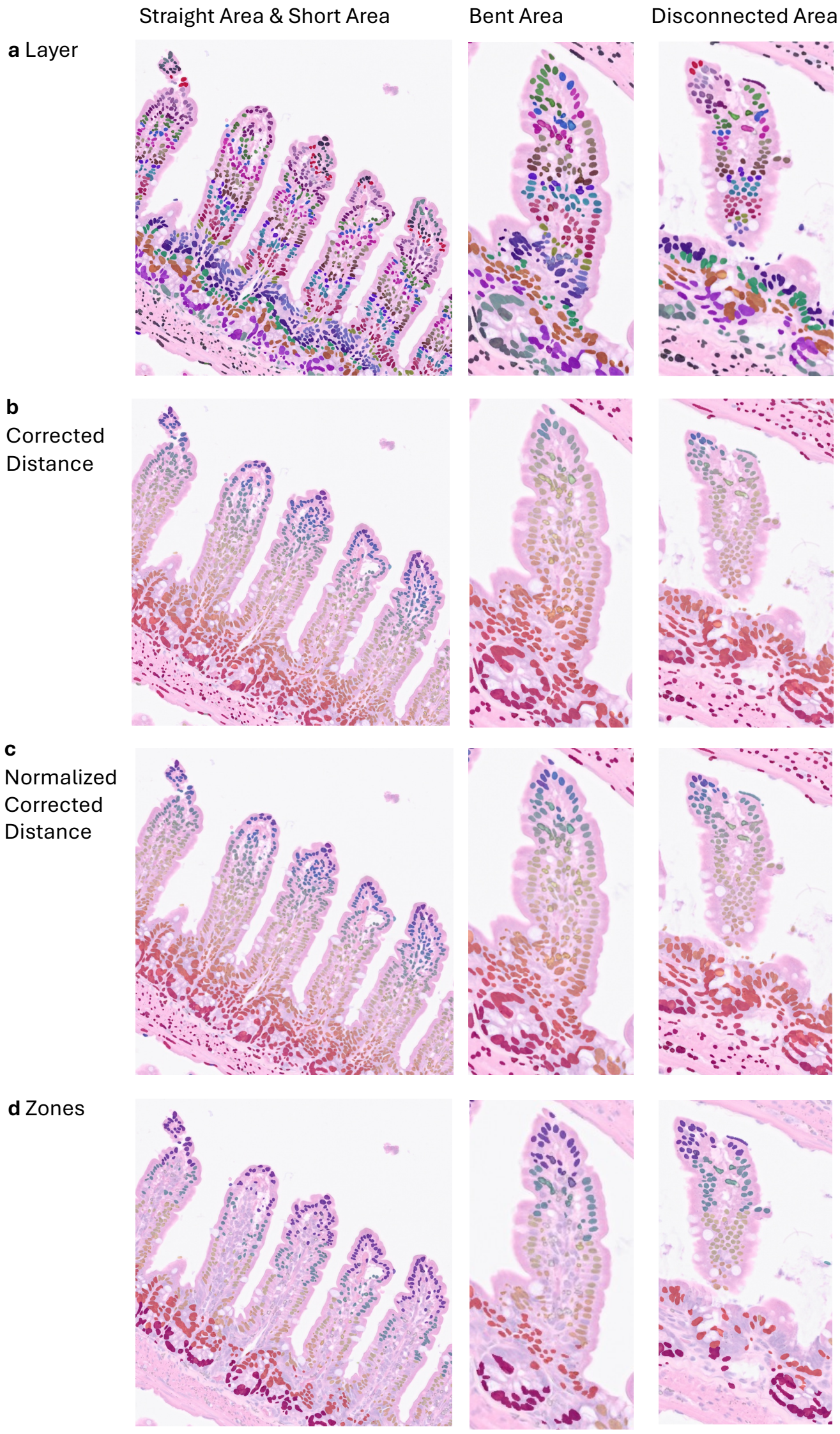

Supplementary Figure 11: **Different Distances Examples Mapped on Real H&E Plot.** (a) Layer. (b) Corrected Distance. (c) Normalized Corrected Distance. (d) Zone.

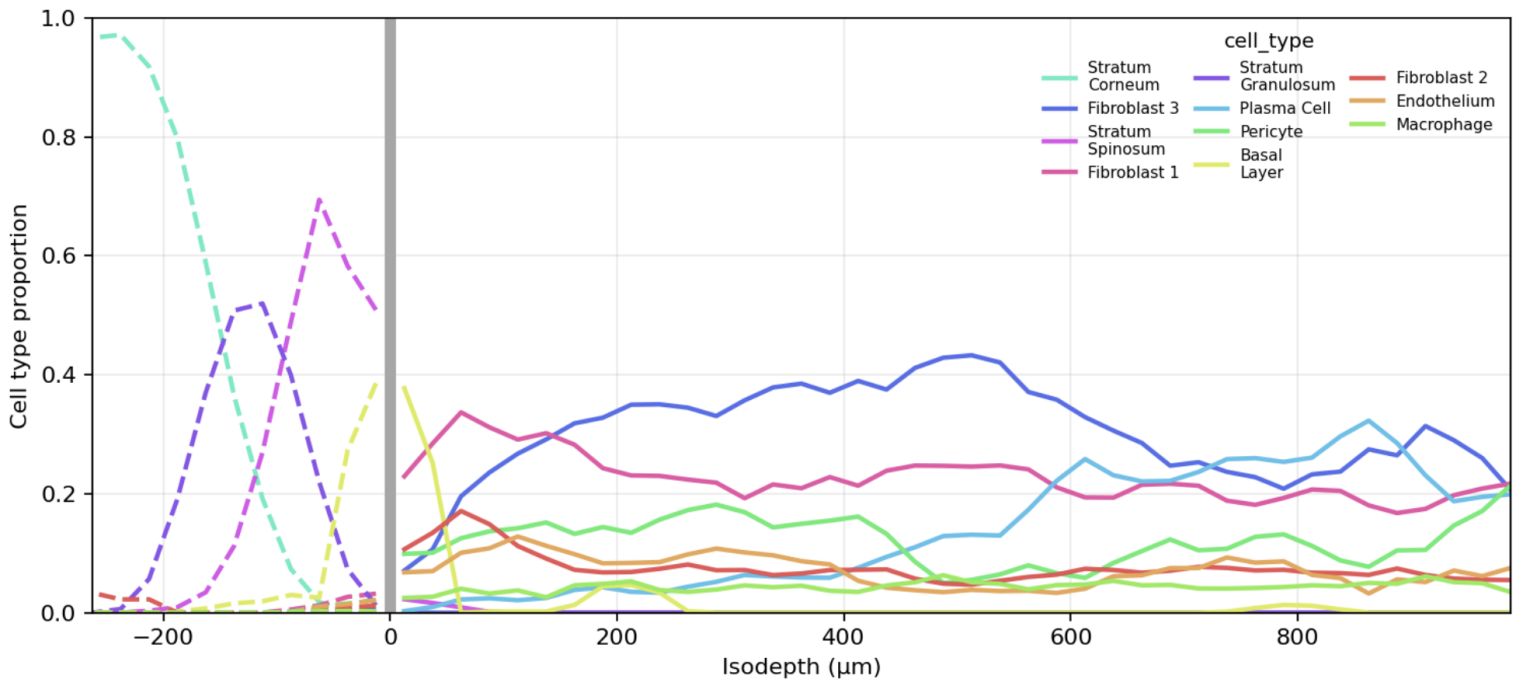

Supplementary Figure 12: **Epidermal zonation across isodepth (basal layer anchored).** Proportions of epidermal cell clusters are shown as a function of SMURF-derived isodepth, with the basal layer set as the zero axis. Isodepth values were generated using the SMURF unrolling framework rather than an external isodepth program: basal-layer cells (cluster 20 from the 10x Genomics clustering results) were used as the origin ($y = 0$), epidermal layers were mapped to $y \leq 0$, and extra-epidermal areas were mapped to $y > 0$. Cell proportions were calculated in binned isodepth intervals, rather than at every unique depth coordinate, by normalizing the cluster counts within each interval to sum to 1.

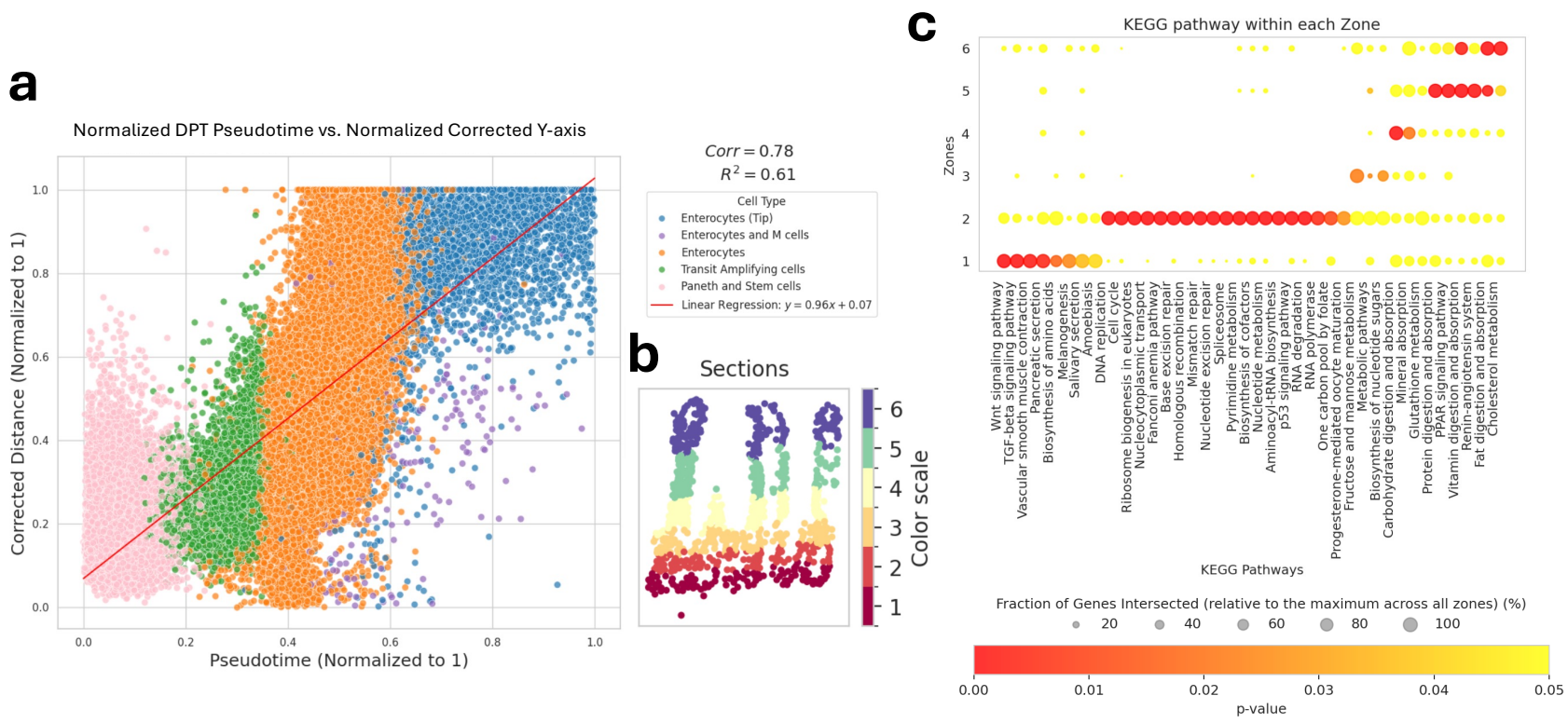

Supplementary Figure 13: **Spatial zoning of enterocytes: pseudotime–depth concordance and KEGG enrichment.** (a) Scatter plot comparing the normalized pseudotime progression score and the normalized corrected y-axis. Each point represents a single cell. The dashed red line indicates a linear fit (Pearson correlation = 0.78). (b) Example of the discrete binning of cells of the intestinal villus into 6 zones along the y-axis. (c) Bubble plot displaying KEGG pathway enrichment of zone-associated genes.

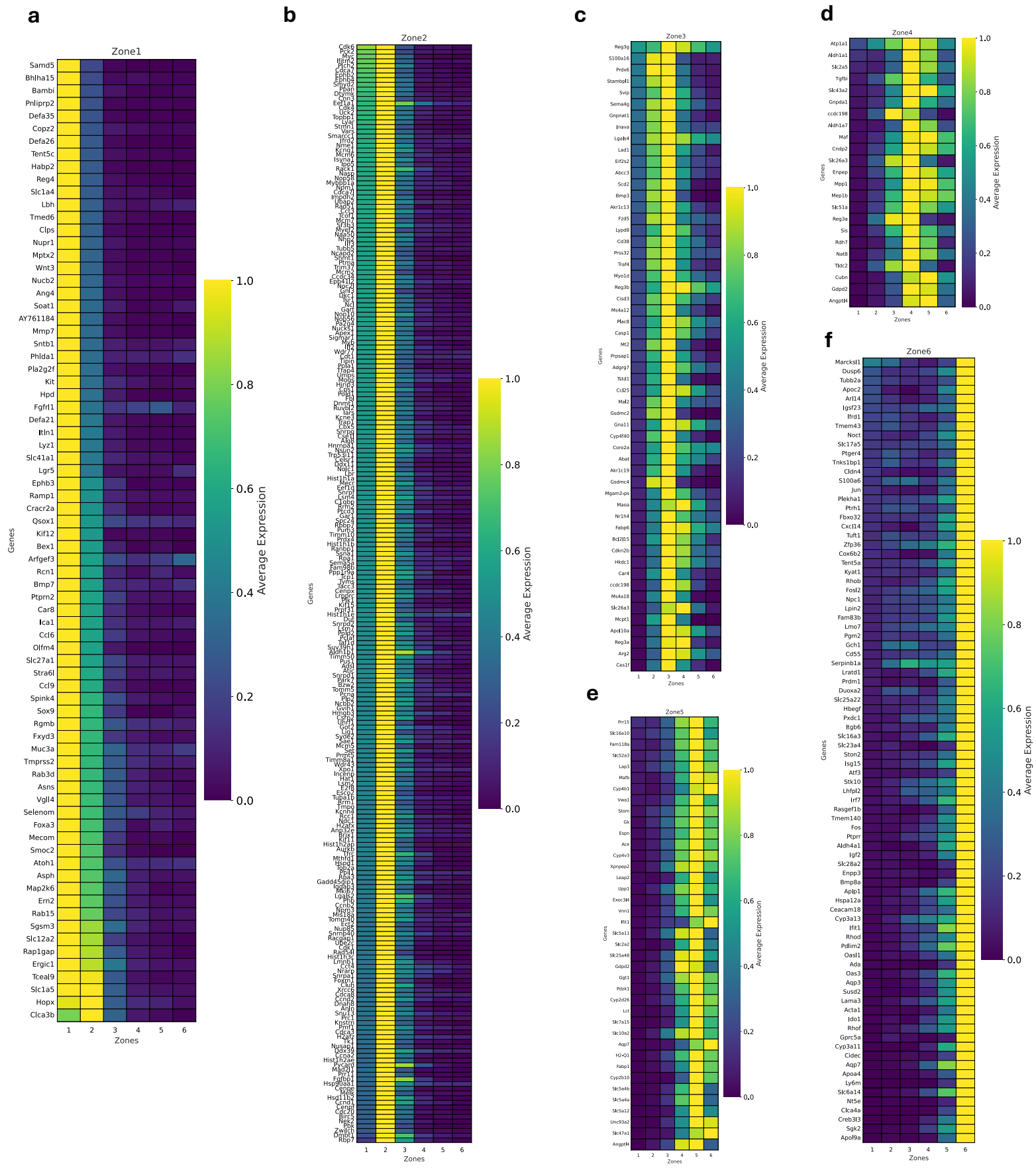

Supplementary Figure 14: **Highly zonated enterocyte genes.** Highly zonated genes were selected using the filtering and manual inspection procedure described in the Supplementary Note subsection "Identification of Highly Zonated Genes". Heatmaps depict normalized gene expression for Zone 1 (a), Zone 2 (b), Zone 3 (c), Zone 4 (d), Zone 5 (e), and Zone 6 (f).

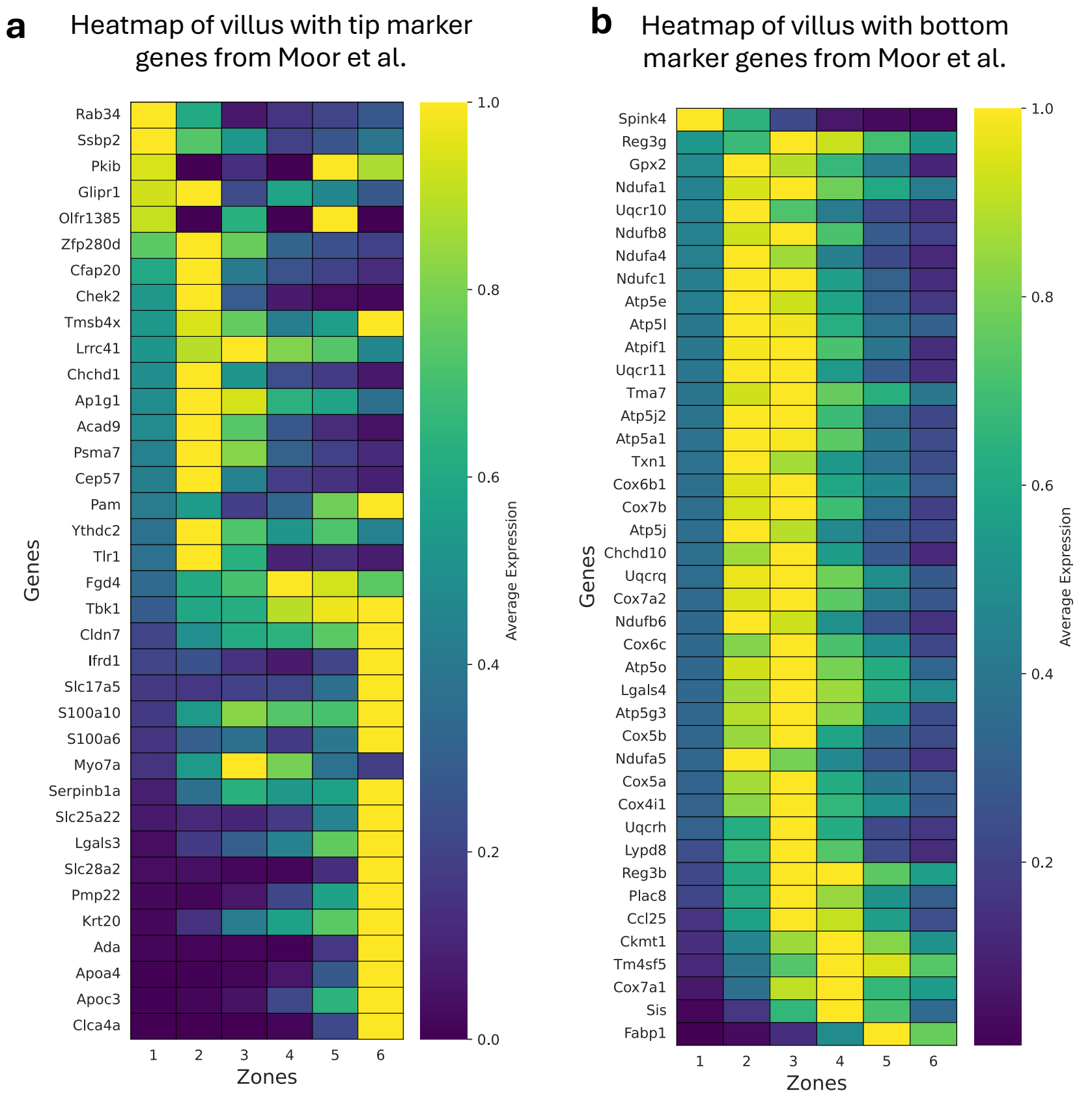

Supplementary Figure 15: **Heatmap of villus top and bottom marker genes from Moor et al. [2] in the ileal SMURF dataset.** (a) Heatmap of villus with top marker genes from Moor et al. (b) Heatmap of villus with bottom marker genes from Moor et al.

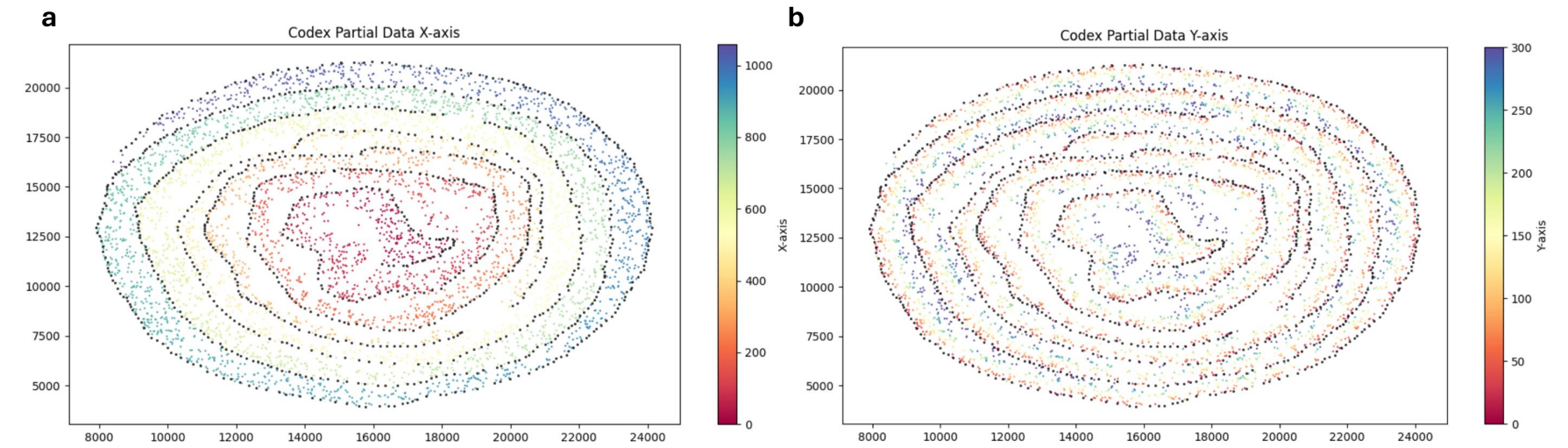

Supplementary Figure 16: **Exploratory application of the SMURF unrolling framework to a published mouse intestine CODEX dataset.** Using the author-reported intestine base trace as the pseudo-x-axis base, cells were projected into an unrolled coordinate system. The resulting layout was broadly consistent with expected proximal-distal and crypt-villus organization. This analysis is intended as a proof-of-concept visualization rather than a validated deployment of SMURF to spatial proteomics data.

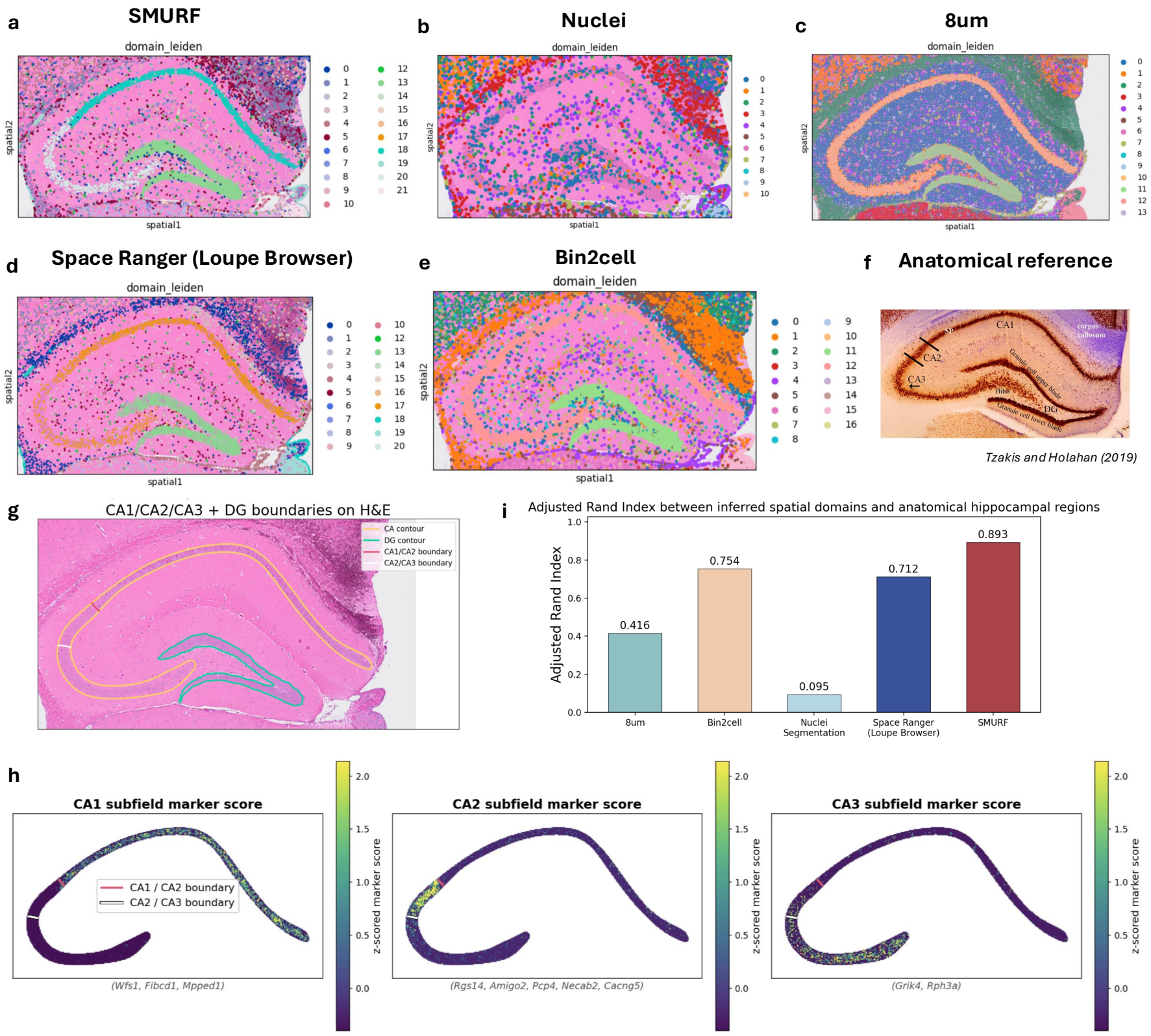

Supplementary Figure 17: **Comparison of downstream spatial domain identification across segmentation methods in the mouse brain.** Spatial Leiden domains in the hippocampal region are shown for (a) SMURF, (b) nuclei-only segmentation, (c) 8 μm binning, (d) Space Ranger (Loupe Browser), and (e) Bin2Cell, all analyzed using the same Squidpy-based downstream workflow. (f) Annotated anatomical reference image of the dorsal hippocampus, adapted from Tzakis and Holahan et al. [3]. (g) H&E image with CA and DG contours and inferred CA1/CA2 and CA2/CA3 boundaries overlaid. (h) CA1, CA2, and CA3 marker-score maps used to define the anatomy-informed hippocampal reference. (i) Adjusted Rand index (ARI) between inferred spatial domains and anatomical hippocampal regions, showing the highest agreement for SMURF.

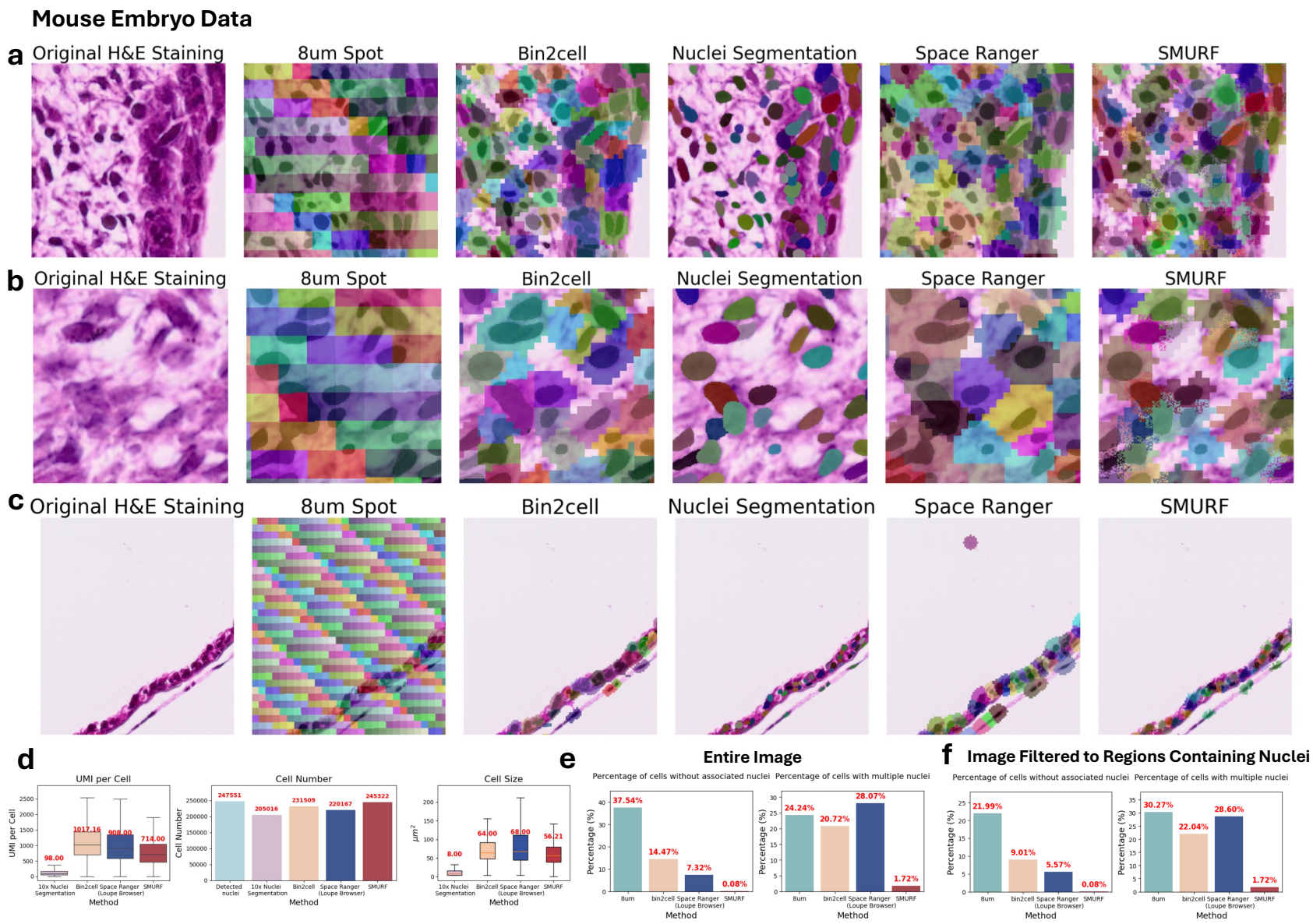

Supplementary Figure 18: **Benchmarking of SMURF-lite against other Visium-HD methods using mouse embryo data.** (a-c) Representative image patches from three regions of the mouse embryo dataset showing how different ST segmentation methods aggregate Visium HD capture spots to define cells. Columns show the original H&E image, 8 μm spots, and the corresponding cell assignments produced by Bin2cell, nuclei segmentation, Space Ranger, and SMURF (SMURF-lite). Capture spots are colored according to their assigned cell identity. (d) Number of UMIs assigned per cell, total cells detected, and cell size for each method. Number of UMIs assigned per cell, total cells detected, and cell size for each method. Numbers above box plots indicate median values, whereas numbers above bars indicate total numbers of detected cells. Box plots show the median, interquartile range and 1.5× interquartile range whiskers; outliers beyond whiskers are not shown. No biological replicate comparison was performed. (e) Percentage of cells without an associated nucleus and percentage of cells containing multiple nuclei, calculated over the entire image. (f) Percentage of cells without an associated nucleus and percentage of cells containing multiple nuclei, calculated after restricting the analysis to regions containing nuclei. Abbreviations: ST, spatial transcriptomics; UMI, Unique Molecular Identifier.

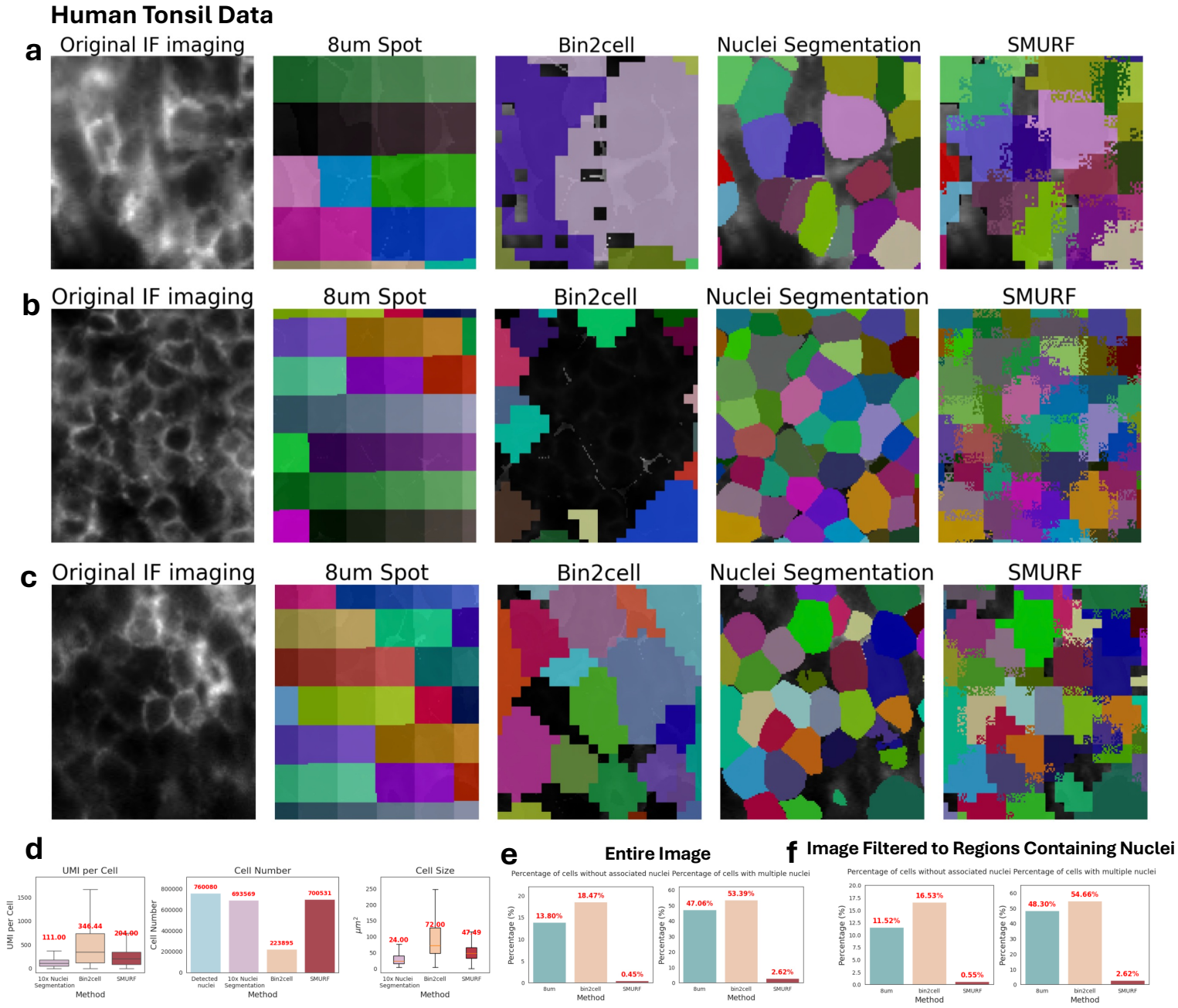

Supplementary Figure 19: **Benchmarking of SMURF-lite against other Visium-HD methods using human tonsil data.** (a-c) Representative image patches from three regions of the human tonsil dataset showing how different ST segmentation methods aggregate Visium HD capture spots to define cells. Columns show the original IF image, 8 μm spots, and the corresponding cell assignments produced by Bin2cell, nuclei segmentation, and SMURF (SMURF-lite). Capture spots are colored according to their assigned cell identity. (d) Number of UMIs assigned per cell, total cells detected, and cell size for each method. Number of UMIs assigned per cell, total cells detected, and cell size for each method. Numbers above box plots indicate median values, whereas numbers above bars indicate total numbers of detected cells. Box plots show the median, interquartile range and 1.5$\times$ interquartile range whiskers; outliers beyond whiskers are not shown. No biological replicate comparison was performed. (e) Percentage of cells without an associated nucleus and percentage of cells containing multiple nuclei, calculated over the entire image. (f) Percentage of cells without an associated nucleus and percentage of cells containing multiple nuclei, calculated after restricting the analysis to regions containing nuclei. Abbreviations: ST, spatial transcriptomics; UMI, Unique Molecular Identifier.

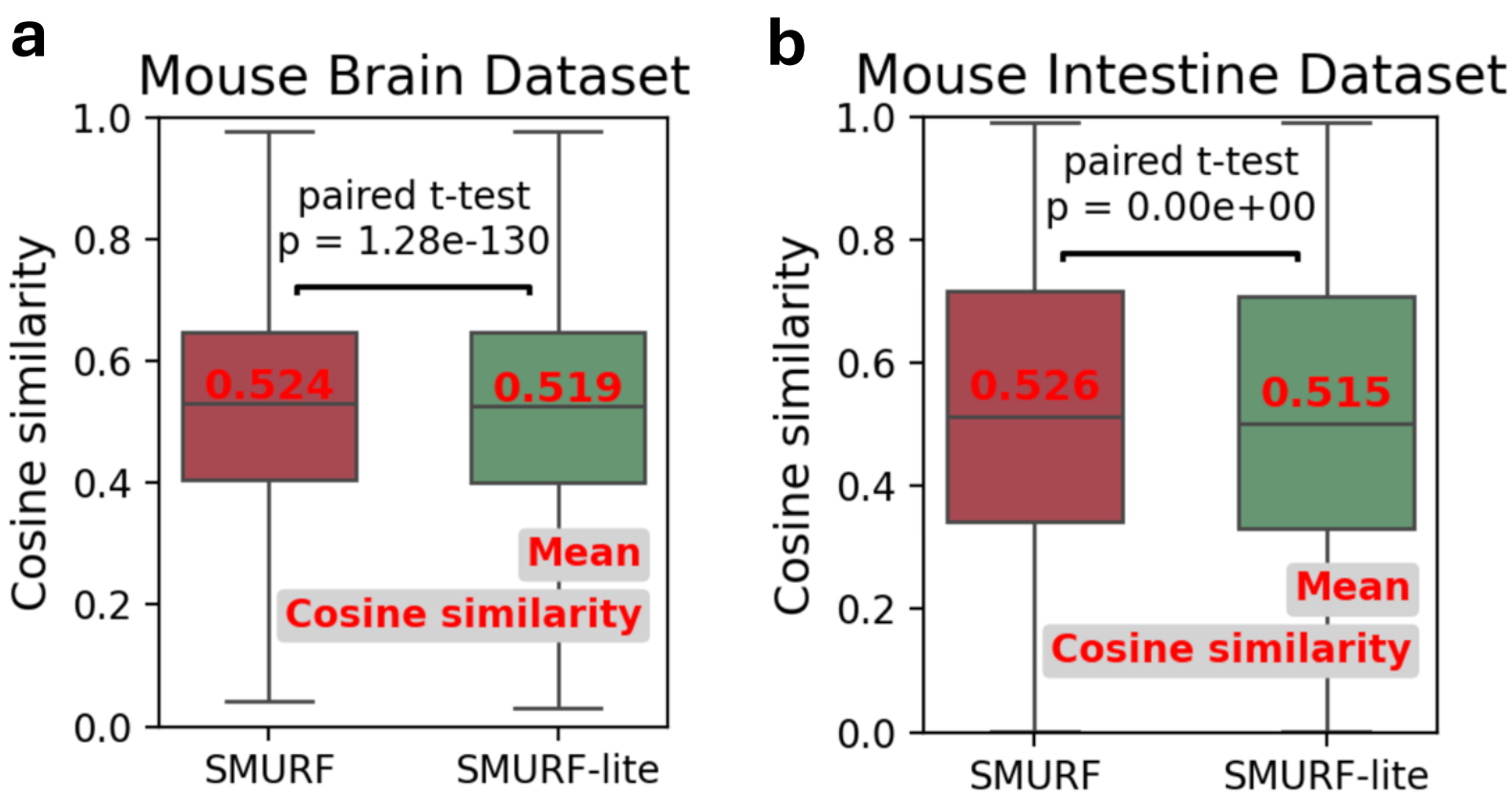

Supplementary Figure 20: **Comparison of cosine similarity for SMURF and SMURF-lite.** We compared cosine similarity between reconstructed cells and the mean expression profile of their assigned clusters for SMURF and SMURF-lite. (a) Mouse brain dataset. (b) Mouse small intestine dataset. Red numbers indicate mean cosine similarity. Box plots show the median, interquartile range and 1.5× interquartile range whiskers; outliers beyond whiskers are not shown. P values were calculated using paired two-sided t-tests comparing matched cells reconstructed by SMURF and SMURF-lite.

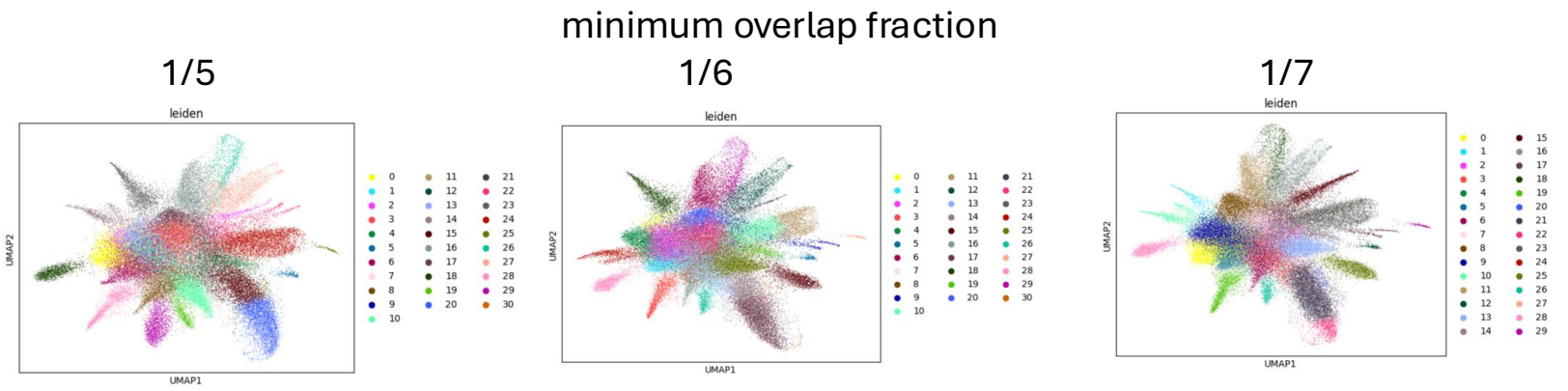

Supplementary Figure 21: **Sensitivity of SMURF to overlap fraction parameter.** We evaluated whether SMURF's transcript assignment was robust to changes in the minimum overlap fraction parameter used for initializing shared-spot assignment. UMAP visualizations and Leiden cluster assignments are shown for thresholds of 1/5, 1/6, and 1/7. Cell assignments were highly similar, demonstrating this parameter will typically not need to be tuned between experiments.

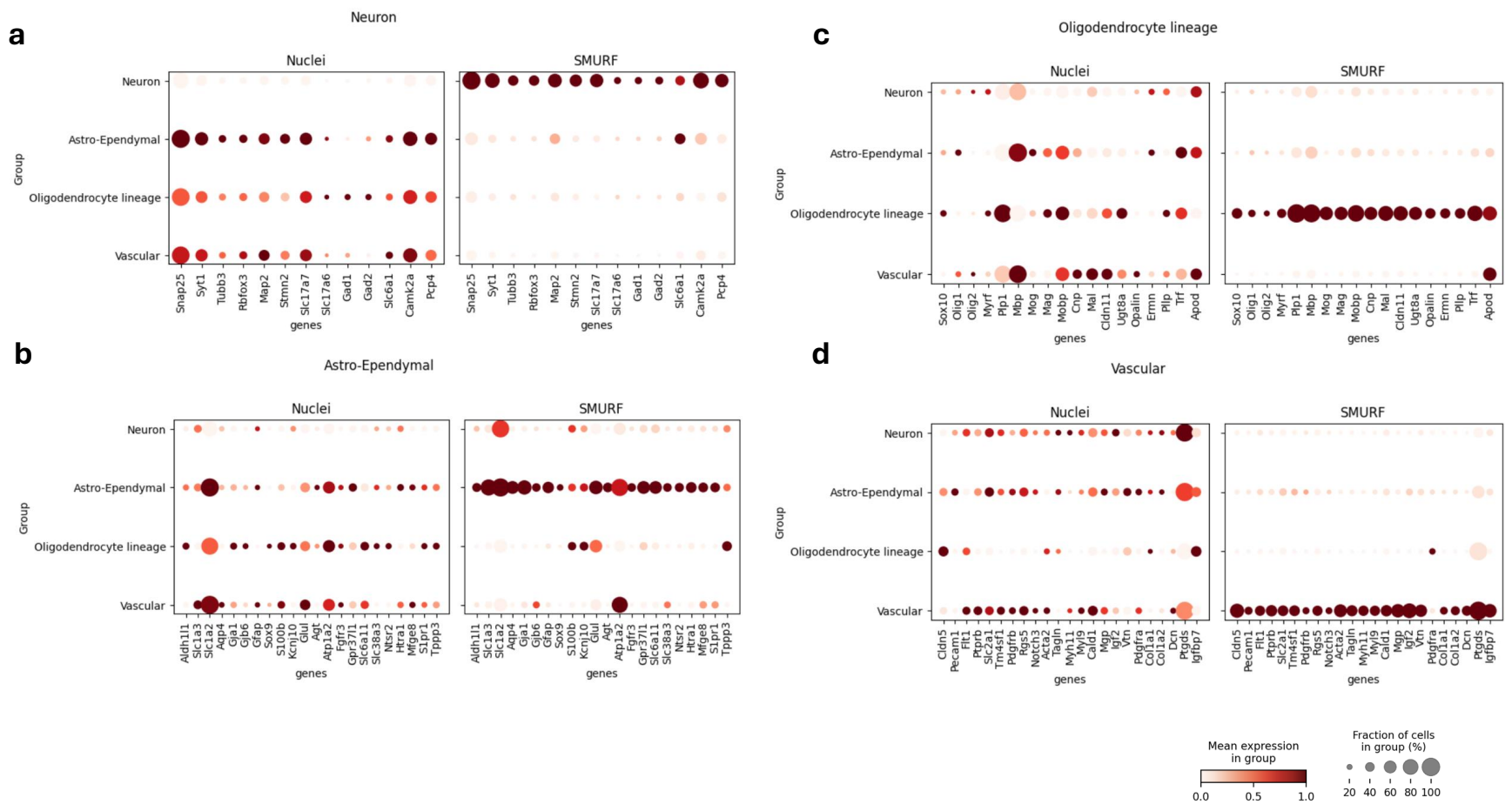

Supplementary Figure 22: **Assessment of cross-cell-type marker contamination in SMURF versus nuclei-only segmentation in the mouse brain dataset.** (a-d) Dot plots showing the expression of canonical marker genes for neurons (a), astro-ependymal cells (b), oligodendrocyte-lineage cells (c), and vascular cells (d), comparing cells defined by nuclei-only segmentation and SMURF segmentation. Dot size indicates the fraction of cells in the cluster expressing each gene, and color indicates mean expression level. Marker specificity was compared across major cell groups to assess whether SMURF expansion increased erroneous cross-cell-type transcript assignment.

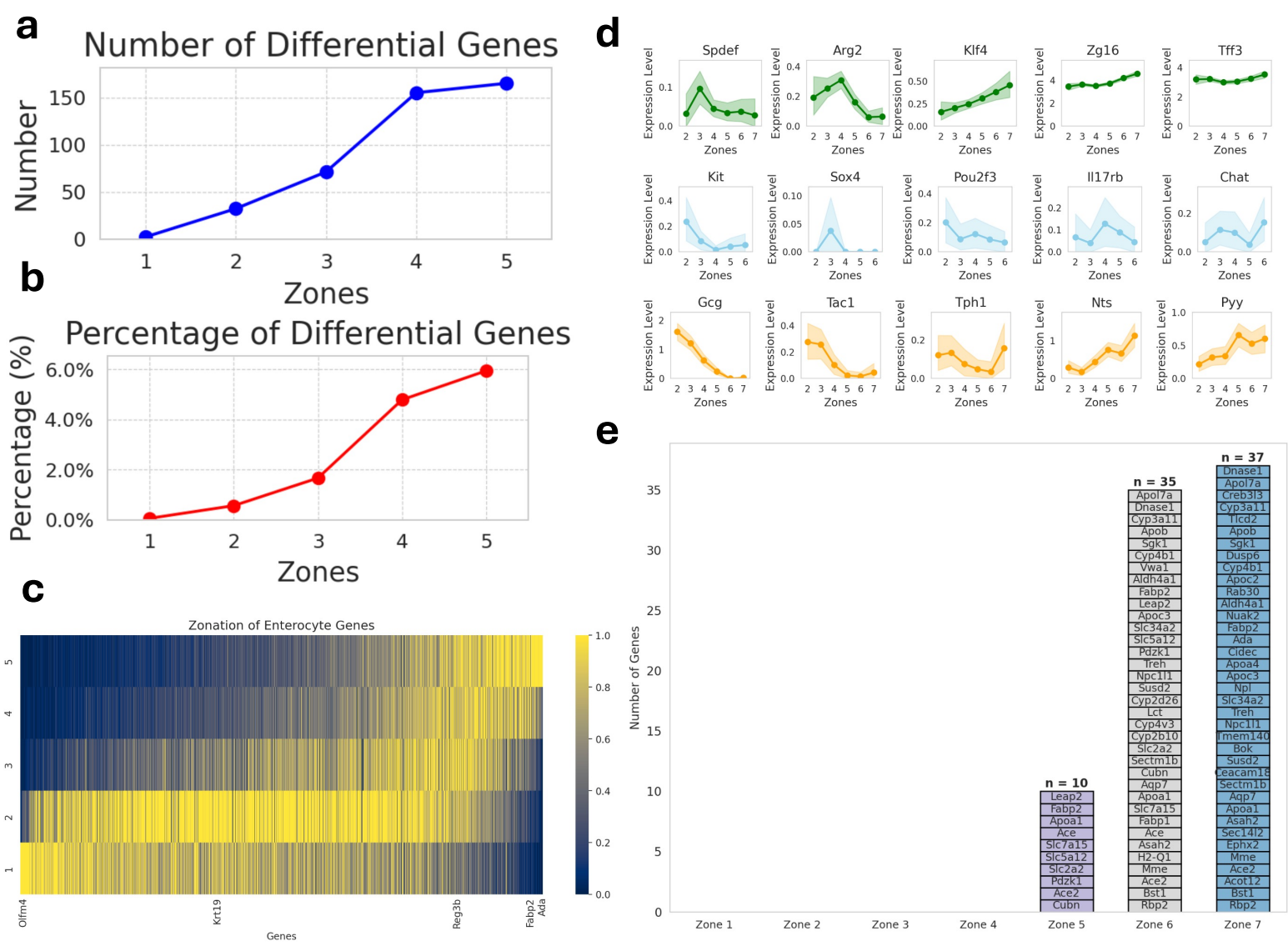

Supplementary Figure 23: **Sensitivity analysis of intestinal zonation to the number of zones chosen: Results for 5 Zones.** (a, b) Number (a) and fraction (b) of genes differentially expressed between proximal and distal regions of villi within each zone under 5-, 6-, and 7-zone discretizations of the crypt–villus axis. Within each zone, mitochondrial genes were excluded and only genes expressed in at least 100 cells were considered. Genes were retained when absolute $\log_2$ fold-change exceeded 1.5 and adjusted $P < 0.001$. Fractions were calculated relative to the number of genes retained after filtering in each zone. (c) Heatmap of normalized expression for enterocyte zonation genes across the 5-zone discretization of the crypt–villus axis. (d) Zonation profiles of representative marker genes across the 5 spatial zones. Expression values were summarized as the mean expression across cells within each zone. Shaded regions indicate 95% bootstrap confidence intervals for the mean, calculated by resampling cells within each zone with replacement (500 iterations). Zone-specific expression is displayed only when 50 or more cells of that lineage are present in the zone. (e) Number and identity of differentially expressed genes in the small bowel resection scRNA-seq dataset (Seiler et al. [4]) for each zone under the 5-zone discretization.

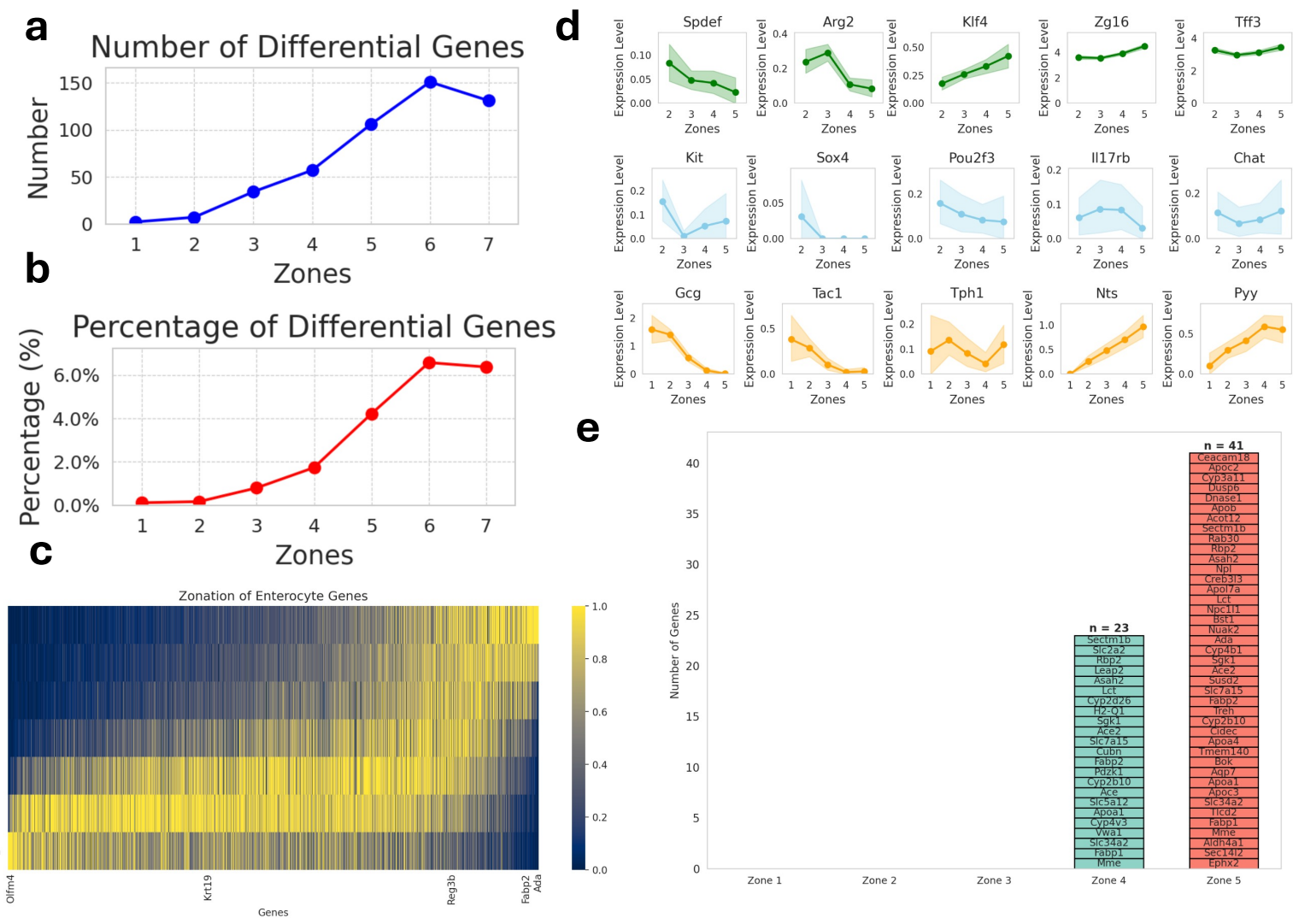

Supplementary Figure 24: **Sensitivity analysis of intestinal zonation to the number of zones chosen: Results for 7 Zones.** (a, b) Number (a) and fraction (b) of genes differentially expressed between proximal and distal regions of villi within each zone under 5-, 6-, and 7-zone discretizations of the crypt–villus axis. Within each zone, mitochondrial genes were excluded and only genes expressed in at least 100 cells were considered. Genes were retained when absolute $\log_2$ fold-change exceeded 1.5 and adjusted $P < 0.001$. Fractions were calculated relative to the number of genes retained after filtering in each zone. (c) Heatmap of normalized expression for enterocyte zonation genes across the 7-zone discretization of the crypt–villus axis. (d) Zonation profiles of representative marker genes across the 7 spatial zones. Expression values were summarized as the mean expression across cells within each zone. Shaded regions indicate 95% bootstrap confidence intervals for the mean, calculated by resampling cells within each zone with replacement (500 iterations). Zone-specific expression is displayed only when 50 or more cells of that lineage are present in the zone. (e) Number and identity of differentially expressed genes in the small bowel resection scRNA-seq dataset (Seiler et al. [4]) for each zone under the 7-zone discretization.

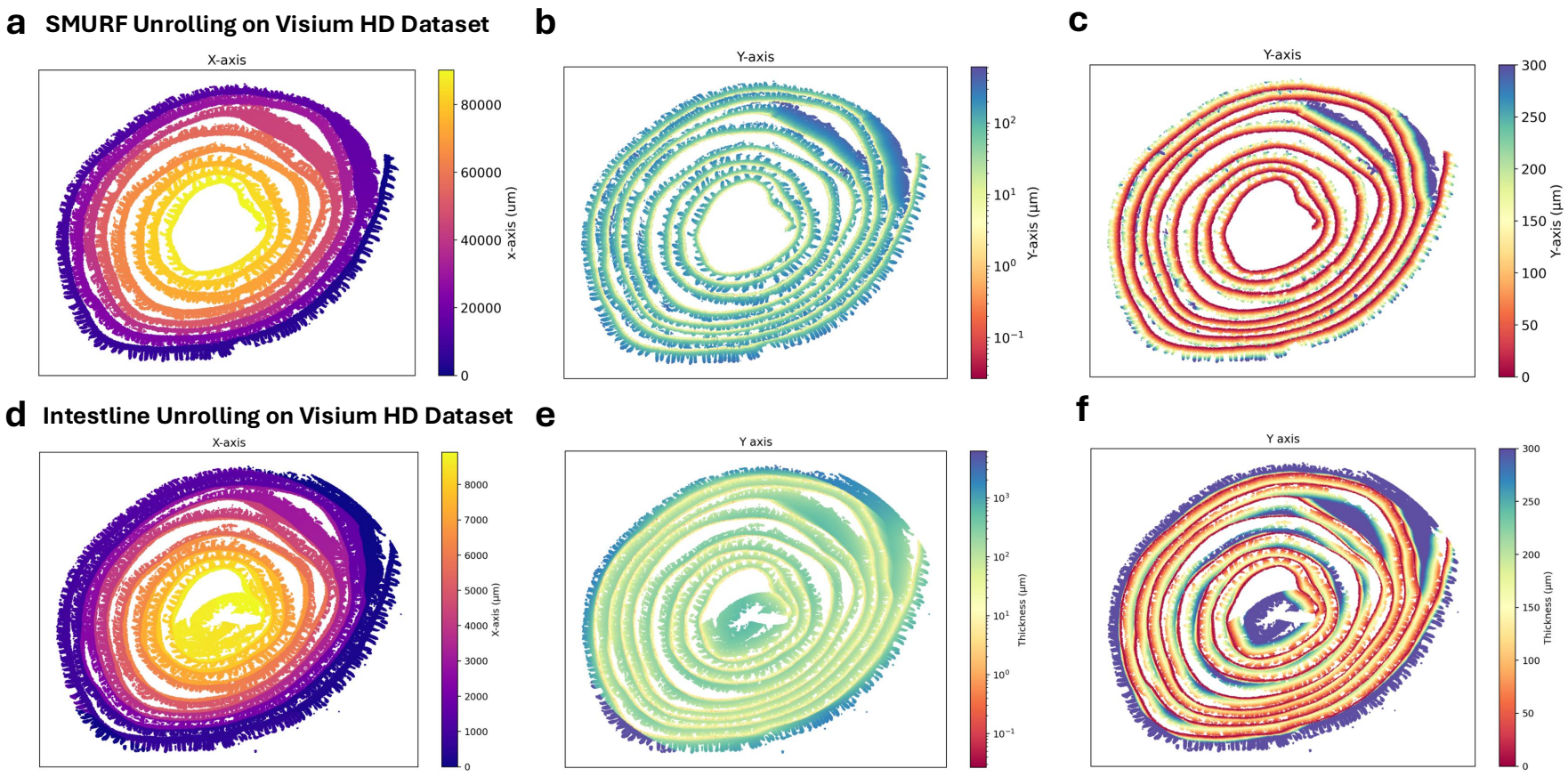

Supplementary Figure 25: **Comparison of Swiss-roll unrolling results obtained with SMURF and IntestLine on Visium HD mouse small intestine data.** (a-c) SMURF-derived unrolling coordinates, showing the x-axis coordinate in (a) and the y-axis coordinate on log and linear color scales in (b) and (c), respectively. (d-f) IntestLine-derived unrolling coordinates, obtained using the same SMURF-derived x-axis and center point as inputs, showing the x-axis coordinate in (d) and the thickness (y-axis) coordinate on log and linear color scales in (e) and (f), respectively. Overall, SMURF yielded unrolling coordinates that were more anatomically coherent than those produced by IntestLine on this Visium HD intestine section.

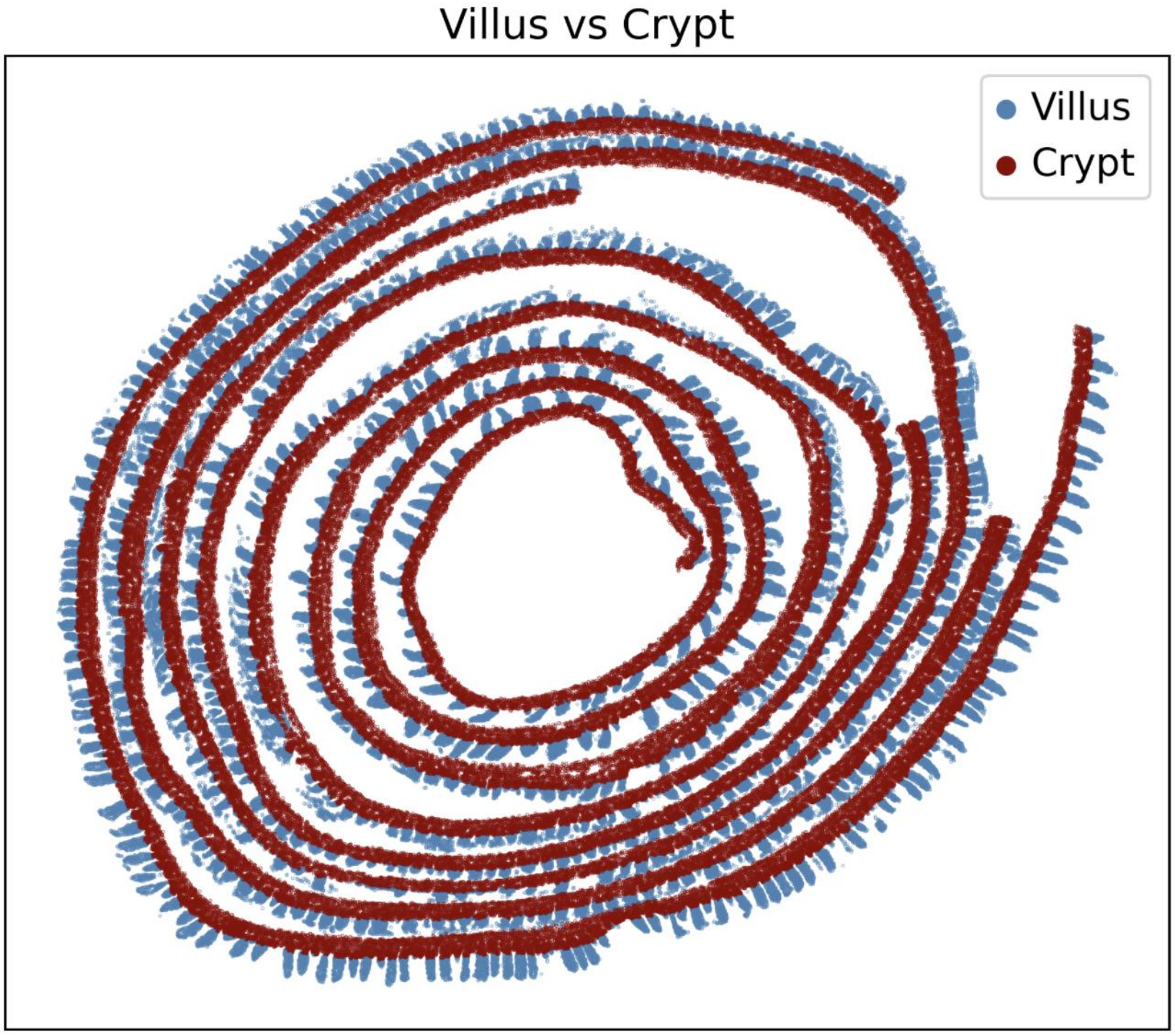

Supplementary Figure 26: **Validation of the Inferred Crypt–Villus Boundary Across the Entire Length of the Mouse Ileum Swiss Roll.**

Supplementary Tables

Supplementary Table 1: Summary of Extended Benchmarking.

Method	Works out of the box	Required codebase modifications	Runtime and computational resource usage (mouse brain dataset)	Successfully benchmarked
SMURF	Yes	None	2.92 h for SMURF and 1.69 h for SMURF-lite (3-run averages)	Yes
Space Ranger (Loupe Browser) [5, 6]	Yes	None	N/A (runs automatically with FASTQ alignment)	Yes (only works for H&E staining)
Bin2cell [7]	Yes	None	5.62 min (3-run average)	Yes
8 μm binning	Yes	None	N/A (runs automatically with FASTQ alignment)	Yes
Baysor [8]	No	None	26.48 h (single run; summed across 3 $\times$ 3 slides)	Yes
SCS [9]	No	Yes	100.51 h (4.12 days; single run summed across 4 $\times$ 5 slides, with GPU; substantially longer without GPU)	Yes
Sparcle [10]	No	Yes	Estimated to be hundreds of years	Failed due to excessive compute requirements
JSTA [11]	No	Yes	Estimated to require months	Failed due to excessive compute requirements

Supplementary Table 2: Comparison of Intestinal Cell-Type Proportions across Studies. Values Are Percentages. NR: Not Reported.

	Cell type proportion (%) by study			
Cell type	SMURF	Haber et al. [12]	Zwick et al. [1]	Xu et al. [13]
Enterocyte	51.6	44.6	63.5	61.0
Paneth and stem	17.5	21.2	8.0	27.9
Goblet	13.8	7.1	11.7	9.7
Transit-amplifying	10.8	20.6	7.1	NR
Enteroendocrine	3.6	4.3	4.1	0.3
Tuft	2.7	2.3	5.6	1.1
Platform	Visium HD	scRNA-seq	scRNA-seq	MERFISH
Tissue / source	Ileum	Crypt isolation, SI	Total epithelium, SI	Ileum

Supplementary Table 3: Cell-Type Composition in the Visium HD Dataset.

Cell type	n	percent
Enterocytes	90145	23.97
Immune cells (excluding Peyer's patches)	57489	15.29
Smooth Muscle cells	45290	12.05
Peyer's patch	42875	11.40
Fibroblasts	36940	9.82
Paneth and Stem cells	30589	8.14
Goblet cells	24061	6.40
Transit Amplifying cells	18903	5.03
Endothelial cells	9850	2.62
Enteroendocrine cells	6365	1.69
Tuft cells	4677	1.24
High-mito cells	4648	1.24
Neurons	4167	1.11

Supplementary Table 4: Average SCENIC Regulon Activity Scores Across Intestinal Zones.

TF	1	2	3	4	5	6	TF	1	2	3	4	5	6
Ahdc1(+)	0.024	0.056	0.076	0.077	0.071	0.061	Ahr(+)	0.050	0.069	0.070	0.058	0.052	0.049
Arid3a(+)	0.226	0.166	0.090	0.058	0.049	0.046	Arnt(+)	0.090	0.073	0.072	0.077	0.077	0.078
Arntl(+)	0.032	0.057	0.053	0.037	0.034	0.035	Atf2(+)	0.050	0.064	0.066	0.060	0.056	0.052
Atf3(+)	0.045	0.062	0.076	0.086	0.105	0.140	Atf4(+)	0.030	0.062	0.060	0.045	0.040	0.043
Bach1(+)	0.035	0.045	0.070	0.093	0.110	0.130	Bach2(+)	0.042	0.083	0.132	0.175	0.177	0.158
Barx2(+)	0.019	0.040	0.066	0.084	0.077	0.058	Bcl6(+)	0.054	0.075	0.089	0.092	0.109	0.164
Bclaf1(+)	0.044	0.058	0.049	0.037	0.033	0.031	Cdc5l(+)	0.042	0.054	0.042	0.033	0.031	0.031
Cdx2(+)	0.056	0.092	0.106	0.089	0.074	0.068	Cebpa(+)	0.052	0.074	0.074	0.061	0.052	0.048
Cebpd(+)	0.060	0.074	0.084	0.076	0.069	0.065	Cebpg(+)	0.051	0.070	0.075	0.072	0.073	0.076
Cnot3(+)	0.040	0.051	0.049	0.044	0.041	0.039	Creb1(+)	0.055	0.073	0.068	0.057	0.054	0.052
Creb3(+)	0.040	0.053	0.058	0.056	0.054	0.050	Creb3l1(+)	0.063	0.077	0.058	0.041	0.035	0.032
Creb3l4(+)	0.295	0.163	0.053	0.028	0.023	0.025	Dbp(+)	0.028	0.047	0.071	0.095	0.096	0.076
Deaf1(+)	0.058	0.070	0.066	0.057	0.053	0.051	Drap1(+)	0.046	0.073	0.065	0.047	0.041	0.039
E2f2(+)	0.046	0.056	0.052	0.043	0.039	0.039	E2f4(+)	0.040	0.061	0.053	0.039	0.034	0.032
E2f7(+)	0.028	0.051	0.044	0.028	0.023	0.021	E2f8(+)	0.053	0.073	0.062	0.046	0.040	0.037
E4f1(+)	0.029	0.053	0.043	0.024	0.017	0.014	Ehf(+)	0.047	0.062	0.057	0.047	0.042	0.040
Elf1(+)	0.031	0.056	0.062	0.057	0.058	0.061	Elf3(+)	0.039	0.055	0.051	0.041	0.037	0.035
Elf4(+)	0.031	0.046	0.050	0.048	0.049	0.054	Elk4(+)	0.036	0.049	0.054	0.052	0.051	0.050
Ep300(+)	0.026	0.053	0.056	0.043	0.035	0.029	Epas1(+)	0.022	0.036	0.048	0.050	0.053	0.057
Esrra(+)	0.138	0.264	0.428	0.476	0.406	0.308	Ets1(+)	0.038	0.044	0.046	0.053	0.069	0.083
Ets2(+)	0.056	0.069	0.057	0.043	0.040	0.040	Etv3(+)	0.022	0.049	0.059	0.049	0.038	0.029
Fos(+)	0.037	0.054	0.074	0.094	0.111	0.125	Fosl2(+)	0.037	0.055	0.079	0.103	0.129	0.161
Foxa1(+)	0.038	0.058	0.046	0.034	0.030	0.029	Foxa3(+)	0.115	0.094	0.067	0.058	0.057	0.055
Foxf1(+)	0.052	0.078	0.097	0.096	0.105	0.130	Foxj2(+)	0.024	0.042	0.057	0.072	0.085	0.082
Foxm1(+)	0.030	0.054	0.036	0.021	0.016	0.011	Foxn3(+)	0.033	0.061	0.065	0.058	0.056	0.059
Foxo1(+)	0.041	0.054	0.056	0.054	0.052	0.048	Foxo4(+)	0.069	0.081	0.079	0.068	0.064	0.063
Gabpa(+)	0.057	0.069	0.067	0.059	0.056	0.055	Gabpb1(+)	0.037	0.054	0.055	0.045	0.040	0.036
Gata4(+)	0.069	0.096	0.117	0.107	0.095	0.087	Gata5(+)	0.036	0.054	0.060	0.059	0.057	0.054
Gata6(+)	0.044	0.058	0.061	0.060	0.062	0.067	Gmeb1(+)	0.079	0.081	0.072	0.063	0.060	0.057
Gtf2ird1(+)	0.056	0.078	0.076	0.060	0.053	0.047	Gtf3c2(+)	0.063	0.055	0.055	0.057	0.058	0.059
Hdac2(+)	0.042	0.070	0.059	0.036	0.025	0.021	Hes1(+)	0.054	0.075	0.063	0.046	0.039	0.036
Hes2(+)	0.023	0.044	0.073	0.097	0.099	0.089	Hes6(+)	0.054	0.067	0.057	0.044	0.039	0.036
Hinfp(+)	0.031	0.058	0.061	0.047	0.038	0.029	Hmbox1(+)	0.080	0.081	0.078	0.071	0.068	0.068
Hmg20b(+)	0.025	0.054	0.079	0.102	0.118	0.117	Hnf1a(+)	0.034	0.051	0.048	0.036	0.031	0.028
Hnf4a(+)	0.125	0.221	0.252	0.220	0.215	0.238	Hnf4g(+)	0.040	0.059	0.081	0.092	0.092	0.087
Hoxb4(+)	0.082	0.084	0.103	0.109	0.102	0.094	Hoxb8(+)	0.069	0.068	0.087	0.102	0.105	0.098
Ing4(+)	0.143	0.110	0.108	0.119	0.124	0.129	Irf1(+)	0.047	0.065	0.078	0.079	0.083	0.094
Irf2(+)	0.038	0.049	0.055	0.055	0.056	0.058	Irf3(+)	0.058	0.062	0.061	0.055	0.052	0.047
Irf6(+)	0.064	0.078	0.101	0.107	0.099	0.091	Irf7(+)	0.040	0.055	0.074	0.090	0.105	0.117
Irf8(+)	0.056	0.072	0.082	0.074	0.064	0.057	Irf9(+)	0.040	0.059	0.074	0.083	0.085	0.079
Isl1(+)	0.090	0.088	0.078	0.071	0.067	0.067	Isx(+)	0.085	0.093	0.105	0.104	0.099	0.097
Jun(+)	0.041	0.057	0.074	0.087	0.105	0.132	Junb(+)	0.033	0.059	0.111	0.168	0.208	0.238
Jund(+)	0.044	0.057	0.066	0.064	0.063	0.064	Klf16(+)	0.025	0.042	0.075	0.115	0.135	0.131
Klf3(+)	0.039	0.051	0.061	0.066	0.071	0.078	Klf4(+)	0.039	0.051	0.075	0.105	0.132	0.157
Klf5(+)	0.040	0.055	0.054	0.046	0.042	0.039	Klf6(+)	0.071	0.135	0.188	0.189	0.201	0.263
Kmt2b(+)	0.035	0.054	0.054	0.045	0.040	0.039	Maf(+)	0.034	0.051	0.065	0.072	0.074	0.071
Mafb(+)	0.048	0.071	0.160	0.299	0.363	0.353	Maff(+)	0.030	0.053	0.067	0.113	0.147	0.179
Mafk(+)	0.037	0.054	0.055	0.049	0.047	0.051	Max(+)	0.012	0.031	0.071	0.131	0.222	0.296
Maz(+)	0.047	0.060	0.051	0.040	0.037	0.036	Mbd2(+)	0.041	0.057	0.052	0.042	0.038	0.037
Mecom(+)	0.123	0.096	0.088	0.094	0.094	0.093	Mga(+)	0.060	0.096	0.121	0.110	0.100	0.090
Mlx(+)	0.029	0.059	0.085	0.087	0.088	0.077	Mnx1(+)	0.126	0.123	0.063	0.041	0.036	0.032
Mxd1(+)	0.050	0.067	0.089	0.108	0.124	0.141	Mxd4(+)	0.051	0.069	0.079	0.071	0.069	0.074
Mxi1(+)	0.033	0.047	0.091	0.159	0.187	0.165	Myb(+)	0.038	0.065	0.040	0.019	0.012	0.009
Myc(+)	0.042	0.059	0.047	0.033	0.029	0.027	Myrf(+)	0.030	0.054	0.049	0.033	0.024	0.016
Nelfb(+)	0.077	0.076	0.072	0.071	0.072	0.072	Neurod1(+)	0.109	0.092	0.099	0.113	0.135	0.182
Nfe2l1(+)	0.032	0.055	0.058	0.045	0.036	0.033	Nfe2l2(+)	0.048	0.046	0.048	0.050	0.054	0.056
Nfya(+)	0.040	0.064	0.056	0.041	0.036	0.033	Npas2(+)	0.060	0.053	0.064	0.081	0.093	0.099
Nr1h4(+)	0.056	0.093	0.122	0.113	0.099	0.093	Nr1i2(+)	0.048	0.060	0.064	0.064	0.060	0.055
Nr1i3(+)	0.032	0.055	0.074	0.096	0.099	0.078	Nr2c2(+)	0.072	0.077	0.098	0.116	0.120	0.118
Nr2f2(+)	0.027	0.060	0.099	0.101	0.087	0.081	Nr3c1(+)	0.043	0.062	0.065	0.057	0.052	0.051
Ovol2(+)	0.031	0.054	0.055	0.043	0.035	0.030	Patz1(+)	0.069	0.077	0.071	0.058	0.054	0.053
Pax4(+)	0.052	0.092	0.084	0.061	0.052	0.055	Pbx1(+)	0.054	0.072	0.074	0.064	0.055	0.051
Pitx1(+)	0.158	0.144	0.139	0.139	0.137	0.134	Pou2f1(+)	0.062	0.076	0.071	0.067	0.067	0.064
Pou2f3(+)	0.033	0.056	0.042	0.032	0.031	0.034	Ppara(+)	0.074	0.120	0.150	0.138	0.116	0.100
Ppard(+)	0.109	0.181	0.232	0.208	0.164	0.138	Prdm1(+)	0.026	0.062	0.102	0.120	0.144	0.182
Pura(+)	0.050	0.063	0.059	0.050	0.046	0.045	Rara(+)	0.057	0.081	0.119	0.130	0.122	0.110
Rel(+)	0.081	0.089	0.111	0.122	0.115	0.103	Rela(+)	0.029	0.056	0.050	0.032	0.024	0.021
Relb(+)	0.032	0.070	0.114	0.137	0.156	0.183	Rfx2(+)	0.073	0.091	0.099	0.099	0.109	0.122
Rfx3(+)	0.029	0.054	0.040	0.024	0.020	0.018	Rreb1(+)	0.035	0.089	0.087	0.041	0.021	0.012
Rxra(+)	0.045	0.078	0.098	0.093	0.086	0.085	Sirt6(+)	0.053	0.054	0.053	0.052	0.052	0.052
Smad2(+)	0.050	0.051	0.052	0.054	0.057	0.062	Smarcc2(+)	0.045	0.070	0.073	0.061	0.052	0.044
Smc3(+)	0.165	0.142	0.142	0.146	0.147	0.149	Sox6(+)	0.023	0.047	0.064	0.067	0.075	0.071
Sox9(+)	0.057	0.056	0.036	0.025	0.023	0.024	Sp1(+)	0.035	0.052	0.047	0.036	0.032	0.029
Sp3(+)	0.035	0.049	0.053	0.051	0.050	0.046	Srebf2(+)	0.046	0.065	0.065	0.055	0.049	0.046
Srf(+)	0.055	0.059	0.059	0.055	0.053	0.056	Stat1(+)	0.038	0.055	0.068	0.074	0.074	0.066
Stat2(+)	0.036	0.059	0.091	0.127	0.148	0.148	Taf1(+)	0.075	0.084	0.086	0.087	0.084	0.076
Taf6(+)	0.043	0.058	0.054	0.044	0.039	0.035	Tbp(+)	0.050	0.067	0.067	0.067	0.065	0.054
Tbx3(+)	0.038	0.050	0.095	0.156	0.186	0.177	Tcf3(+)	0.045	0.053	0.044	0.037	0.035	0.034
Tcf4(+)	0.137	0.124	0.135	0.143	0.154	0.167	Tcf7l1(+)	0.026	0.061	0.092	0.121	0.147	0.155
Tfap4(+)	0.071	0.065	0.042	0.029	0.026	0.025	Tfdp1(+)	0.046	0.062	0.053	0.040	0.035	0.033
Tfeb(+)	0.022	0.037	0.048	0.052	0.069	0.092	Thra(+)	0.032	0.047	0.050	0.046	0.042	0.037
Usf2(+)	0.062	0.065	0.069	0.069	0.067	0.061	Vdr(+)	0.044	0.064	0.063	0.052	0.047	0.044
Vezf1(+)	0.051	0.071	0.067	0.053	0.047	0.044	Ybx1(+)	0.037	0.057	0.052	0.039	0.033	0.030
Yy1(+)	0.042	0.054	0.052	0.045	0.043	0.044	Zbtb14(+)	0.036	0.052	0.046	0.034	0.028	0.024
Zbtb33(+)	0.039	0.060	0.058	0.046	0.040	0.037	Zbtb5(+)	0.046	0.078	0.077	0.064	0.059	0.060
Zbtb7a(+)	0.042	0.061	0.062	0.052	0.047	0.044	Zbtb7b(+)	0.037	0.049	0.057	0.058	0.059	0.060
Zeb2(+)	0.471	0.192	0.031	0.011	0.008	0.007	Zfp148(+)	0.030	0.054	0.056	0.050	0.046	0.042
Zfp182(+)	0.097	0.090	0.101	0.111	0.134	0.206	Zfp217(+)	0.073	0.112	0.113	0.087	0.070	0.055
Zfp280d(+)	0.113	0.088	0.088	0.094	0.096	0.097	Zfp3(+)	0.031	0.064	0.083	0.087	0.081	0.075
Zfp384(+)	0.039	0.056	0.053	0.045	0.042	0.042	Zfp398(+)	0.052	0.081	0.081	0.065	0.055	0.045
Zfp574(+)	0.089	0.088	0.086	0.081	0.080	0.080	Zfp579(+)	0.278	0.232	0.239	0.256	0.261	0.265
Zfp59(+)	0.032	0.057	0.051	0.039	0.030	0.025	Zfp607a(+)	0.085	0.086	0.075	0.069	0.068	0.068
Zfp637(+)	0.031	0.067	0.077	0.062	0.047	0.038	Zfp639(+)	0.016	0.029	0.028	0.044	0.065	0.064
Zfx(+)	0.052	0.065	0.069	0.068	0.072	0.077							

Supplementary Table 5: Comparison of Empirical Distribution of Cellular Transcript Counts versus Statistical Model Expectation in Mouse Brain Dataset.

Cell type	Cell number	Gene number	Model	Number and percentage of genes over x percentage points away from expected fraction							
				1 pp count	1 pp percentage	5 pp count	5 pp percentage	10 pp count	10 pp percentage	20 pp count	20 pp percentage
0	3993	1183	1. Poisson	1165.00	98.48	449.00	37.95	242.00	20.46	131.00	11.07
0	3993	1183	2. NB common	358.00	30.26	60.00	5.07	28.00	2.37	15.00	1.27
0	3993	1183	3. NB gene-wise	76.00	6.42	11.00	0.93	4.00	0.34	2.00	0.17
0	3993	1183	4. ZINB	3.00	0.25	0.00	0.00	0.00	0.00	0.00	0.00
5	2497	481	1. Poisson	481.00	100.00	309.00	64.24	163.00	33.89	93.00	19.33
5	2497	481	2. NB common	261.00	54.26	53.00	11.02	25.00	5.20	10.00	2.08
5	2497	481	3. NB gene-wise	93.00	19.33	22.00	4.57	8.00	1.66	2.00	0.42
5	2497	481	4. ZINB	5.00	1.04	1.00	0.21	0.00	0.00	0.00	0.00
7	4217	81	1. Poisson	81.00	100.00	50.00	61.73	22.00	27.16	10.00	12.35
7	4217	81	2. NB common	56.00	69.14	12.00	14.81	6.00	7.41	2.00	2.47
7	4217	81	3. NB gene-wise	12.00	14.81	0.00	0.00	0.00	0.00	0.00	0.00
7	4217	81	4. ZINB	12.00	14.81	0.00	0.00	0.00	0.00	0.00	0.00
8	2885	45	1. Poisson	44.00	97.78	37.00	82.22	27.00	60.00	14.00	31.11
8	2885	45	2. NB common	41.00	91.11	18.00	40.00	10.00	22.22	4.00	8.89
8	2885	45	3. NB gene-wise	14.00	31.11	2.00	4.44	0.00	0.00	0.00	0.00
8	2885	45	4. ZINB	14.00	31.11	2.00	4.44	0.00	0.00	0.00	0.00
10	2621	324	1. Poisson	324.00	100.00	272.00	83.95	149.00	45.99	73.00	22.53
10	2621	324	2. NB common	186.00	57.41	42.00	12.96	19.00	5.86	7.00	2.16
10	2621	324	3. NB gene-wise	44.00	13.58	3.00	0.93	2.00	0.62	0.00	0.00
10	2621	324	4. ZINB	11.00	3.40	1.00	0.31	0.00	0.00	0.00	0.00
12	2058	74	1. Poisson	74.00	100.00	73.00	98.65	73.00	98.65	48.00	64.86
12	2058	74	2. NB common	71.00	95.95	58.00	78.38	30.00	40.54	14.00	18.92
12	2058	74	3. NB gene-wise	16.00	21.62	5.00	6.76	2.00	2.70	0.00	0.00
12	2058	74	4. ZINB	10.00	13.51	5.00	6.76	2.00	2.70	0.00	0.00
14	3216	100	1. Poisson	96.00	96.00	57.00	57.00	39.00	39.00	21.00	21.00
14	3216	100	2. NB common	77.00	77.00	37.00	37.00	17.00	17.00	4.00	4.00
14	3216	100	3. NB gene-wise	23.00	23.00	4.00	4.00	2.00	2.00	0.00	0.00
14	3216	100	4. ZINB	23.00	23.00	4.00	4.00	2.00	2.00	0.00	0.00
15	3697	37	1. Poisson	35.00	94.59	17.00	45.95	14.00	37.84	7.00	18.92
15	3697	37	2. NB common	31.00	83.78	14.00	37.84	8.00	21.62	0.00	0.00
15	3697	37	3. NB gene-wise	11.00	29.73	0.00	0.00	0.00	0.00	0.00	0.00
15	3697	37	4. ZINB	10.00	27.03	0.00	0.00	0.00	0.00	0.00	0.00
18	2142	54	1. Poisson	54.00	100.00	53.00	98.15	50.00	92.59	30.00	55.56
18	2142	54	2. NB common	53.00	98.15	40.00	74.07	19.00	35.19	8.00	14.81
18	2142	54	3. NB gene-wise	16.00	29.63	4.00	7.41	0.00	0.00	0.00	0.00
18	2142	54	4. ZINB	17.00	31.48	4.00	7.41	0.00	0.00	0.00	0.00
19	3939	896	1. Poisson	877.00	97.88	282.00	31.47	165.00	18.42	81.00	9.04
19	3939	896	2. NB common	304.00	33.93	75.00	8.37	28.00	3.12	13.00	1.45
19	3939	896	3. NB gene-wise	49.00	5.47	12.00	1.34	8.00	0.89	4.00	0.45
19	3939	896	4. ZINB	41.00	4.58	11.00	1.23	7.00	0.78	3.00	0.33
23	3034	118	1. Poisson	118.00	100.00	93.00	78.81	53.00	44.92	25.00	21.19
23	3034	118	2. NB common	107.00	90.68	53.00	44.92	27.00	22.88	12.00	10.17
23	3034	118	3. NB gene-wise	20.00	16.95	4.00	3.39	1.00	0.85	0.00	0.00
23	3034	118	4. ZINB	21.00	17.80	4.00	3.39	1.00	0.85	0.00	0.00

Note: pp = percentage points.

Supplementary Table 6: Comparison of Empirical Distribution of Cellular Transcript Counts versus Statistical Model Expectation in Mouse Small Intestine Dataset.

Cell type	Cell number	Gene number	Model	Number and percentage of genes over x percentage points away from expected fraction							
				1 pp count	1 pp percentage	5 pp count	5 pp percentage	10 pp count	10 pp percentage	20 pp count	20 pp percentage
1	18915	7457	1. Poisson	240.00	3.22	83.00	1.11	56.00	0.75	36.00	0.48
1	18915	7457	2. NB common	160.00	2.15	58.00	0.78	31.00	0.42	21.00	0.28
1	18915	7457	3. NB gene-wise	16.00	0.21	3.00	0.04	1.00	0.01	0.00	0.00
1	18915	7457	4. ZINB	12.00	0.16	2.00	0.03	1.00	0.01	0.00	0.00
3	10589	5016	1. Poisson	198.00	3.95	82.00	1.63	59.00	1.18	36.00	0.72
3	10589	5016	2. NB common	147.00	2.93	59.00	1.18	35.00	0.70	23.00	0.46
3	10589	5016	3. NB gene-wise	22.00	0.44	7.00	0.14	5.00	0.10	3.00	0.06
3	10589	5016	4. ZINB	13.00	0.26	2.00	0.04	1.00	0.02	0.00	0.00
4	15524	5305	1. Poisson	79.00	1.49	32.00	0.60	18.00	0.34	12.00	0.23
4	15524	5305	2. NB common	69.00	1.30	23.00	0.43	14.00	0.26	7.00	0.13
4	15524	5305	3. NB gene-wise	4.00	0.08	0.00	0.00	0.00	0.00	0.00	0.00
4	15524	5305	4. ZINB	4.00	0.08	0.00	0.00	0.00	0.00	0.00	0.00
5	26731	7680	1. Poisson	88.00	1.15	28.00	0.36	16.00	0.21	10.00	0.13
5	26731	7680	2. NB common	65.00	0.85	19.00	0.25	11.00	0.14	8.00	0.10
5	26731	7680	3. NB gene-wise	5.00	0.07	2.00	0.03	2.00	0.03	2.00	0.03
5	26731	7680	4. ZINB	2.00	0.03	0.00	0.00	0.00	0.00	0.00	0.00
7	13578	7285	1. Poisson	146.00	2.00	52.00	0.71	34.00	0.47	22.00	0.30
7	13578	7285	2. NB common	111.00	1.52	41.00	0.56	28.00	0.38	16.00	0.22
7	13578	7285	3. NB gene-wise	11.00	0.15	1.00	0.01	0.00	0.00	0.00	0.00
7	13578	7285	4. ZINB	14.00	0.19	1.00	0.01	0.00	0.00	0.00	0.00
8	26374	9519	1. Poisson	106.00	1.11	37.00	0.39	27.00	0.28	15.00	0.16
8	26374	9519	2. NB common	72.00	0.76	25.00	0.26	17.00	0.18	10.00	0.11
8	26374	9519	3. NB gene-wise	8.00	0.08	1.00	0.01	0.00	0.00	0.00	0.00
8	26374	9519	4. ZINB	8.00	0.08	1.00	0.01	0.00	0.00	0.00	0.00
9	35860	7966	1. Poisson	149.00	1.87	58.00	0.73	39.00	0.49	28.00	0.35
9	35860	7966	2. NB common	77.00	0.97	29.00	0.36	20.00	0.25	14.00	0.18
9	35860	7966	3. NB gene-wise	14.00	0.18	3.00	0.04	2.00	0.03	0.00	0.00
9	35860	7966	4. ZINB	11.00	0.14	3.00	0.04	2.00	0.03	0.00	0.00
10	33047	10164	1. Poisson	280.00	2.75	82.00	0.81	49.00	0.48	35.00	0.34
10	33047	10164	2. NB common	142.00	1.40	47.00	0.46	27.00	0.27	21.00	0.21
10	33047	10164	3. NB gene-wise	23.00	0.23	1.00	0.01	0.00	0.00	0.00	0.00
10	33047	10164	4. ZINB	23.00	0.23	1.00	0.01	0.00	0.00	0.00	0.00
11	30751	8183	1. Poisson	228.00	2.79	70.00	0.86	49.00	0.60	28.00	0.34
11	30751	8183	2. NB common	111.00	1.36	38.00	0.46	22.00	0.27	14.00	0.17
11	30751	8183	3. NB gene-wise	15.00	0.18	5.00	0.06	1.00	0.01	1.00	0.01
11	30751	8183	4. ZINB	2.00	0.02	0.00	0.00	0.00	0.00	0.00	0.00
12	17357	3586	1. Poisson	85.00	2.37	33.00	0.92	23.00	0.64	15.00	0.42
12	17357	3586	2. NB common	61.00	1.70	19.00	0.53	13.00	0.36	9.00	0.25
12	17357	3586	3. NB gene-wise	5.00	0.14	0.00	0.00	0.00	0.00	0.00	0.00
12	17357	3586	4. ZINB	5.00	0.14	0.00	0.00	0.00	0.00	0.00	0.00
13	18891	6897	1. Poisson	322.00	4.67	119.00	1.73	75.00	1.09	51.00	0.74
13	18891	6897	2. NB common	153.00	2.22	54.00	0.78	25.00	0.36	17.00	0.25
13	18891	6897	3. NB gene-wise	24.00	0.35	6.00	0.09	5.00	0.07	1.00	0.01
13	18891	6897	4. ZINB	20.00	0.29	4.00	0.06	2.00	0.03	0.00	0.00
21	30385	7491	1. Poisson	25.00	0.33	9.00	0.12	5.00	0.07	4.00	0.05
21	30385	7491	2. NB common	13.00	0.17	4.00	0.05	2.00	0.03	1.00	0.01
21	30385	7491	3. NB gene-wise	4.00	0.05	1.00	0.01	0.00	0.00	0.00	0.00
21	30385	7491	4. ZINB	3.00	0.04	1.00	0.01	0.00	0.00	0.00	0.00
23	32848	6293	1. Poisson	54.00	0.86	22.00	0.35	8.00	0.13	2.00	0.03
23	32848	6293	2. NB common	52.00	0.83	15.00	0.24	11.00	0.17	1.00	0.02
23	32848	6293	3. NB gene-wise	1.00	0.02	0.00	0.00	0.00	0.00	0.00	0.00
23	32848	6293	4. ZINB	0.00	0.00	0.00	0.00	0.00	0.00	0.00	0.00
25	34382	9252	1. Poisson	69.00	0.75	31.00	0.34	15.00	0.16	14.00	0.15
25	34382	9252	2. NB common	51.00	0.55	18.00	0.19	12.00	0.13	9.00	0.10
25	34382	9252	3. NB gene-wise	12.00	0.13	4.00	0.04	2.00	0.02	0.00	0.00
25	34382	9252	4. ZINB	10.00	0.11	2.00	0.02	2.00	0.02	0.00	0.00

Note: pp=percentage points.

Supplementary Table 7: Percentage of Epithelial Cell Types Assigned to the Crypt and Villus Compartments Based on Intact Villi.

**Cell type**	**crypt (%)**	**villus (%)**
Enterocytes	39.62	60.38
Transit Amplifying cells	99.43	0.57
Paneth and stem cells	99.74	0.26

Supplementary Notes

Extended benchmarking of additional cell segmentation methods

We benchmarked the performance of SMURF soft-segmentation against multiple packages that have been used for spatial transcriptomic segmentation. We used the mouse brain dataset from 10x Genomics for our detailed benchmarking. This supplementary note extends the discussion of benchmarking related to Figure 2, where we benchmarked SMURF against the four algorithms specifically designed for Visium HD data using mouse brain and small intestine data. Here we describe additional benchmarking of these four methods in the mouse small intestine (Supplementary Figure 1). We also extend our benchmarking by comparing SMURF against four additional algorithms not originally designed to run on Visium HD data, but which could be refactored to do so, as shown in Supplementary Table 1 and Supplementary Figure 2.

Because the methods we benchmark here were originally developed for imaging-based spatial transcriptomics or alternative high-resolution platforms other than Visium HD, their application required varying degrees of input reformatting, code adaptation, or additional engineering. the The "Implementation and Interpretation of Benchmarking Experiments" section below specifically details the required computational adaptations. We therefore report not only segmentation performance, but also whether each method could be applied directly to Visium HD inputs, the extent of codebase modification required, computational runtime, and whether the full benchmarking could be completed with reasonable computational effort (Supplementary Table 1).

Space Ranger (Loupe Browser) [5, 6].

Space Ranger performs the primary analysis for Visium HD, whereas Loupe Browser is used for visualization; here, we refer to this workflow as Space Ranger (Loupe Browser) for consistency with Supplementary Table 1. This workflow could be applied directly to H&E-based Visium HD data without code modification and therefore served as an important practical comparator. In the mouse brain dataset, Space Ranger merged neighboring cells more often than SMURF: when the denominator was restricted to the nuclei-containing region, 21.36% of Space Ranger segmented cells contained multiple nuclei, compared with 0.95% for SMURF. Using the entire imaged area as the denominator, the corresponding fractions were 21.51% for Space Ranger and 0.95% for SMURF. Although Space Ranger assigned more UMIs per cell in some regions, this was accompanied by dramatically increased cell merging and reduced single-cell resolution (Main Text Figure 2a-c; Supplementary Figure 2). A directly comparable standalone runtime for the segmentation component was not available because the output is generated within the standard 10x Genomics pipeline. This workflow is also currently restricted to H&E input.

Baysor [8].

Baysor could not be applied natively to Visium HD data. To enable benchmarking, we reformatted the Visium HD mouse brain dataset into the molecule- and nucleus-based input representation expected by Baysor and divided the full section into 3×3 tiles to reduce memory usage. Even after these adaptations, Baysor produced biologically implausible segmentations, identifying 2,293,396 cells across the dataset, far

exceeding the 57,583 nuclei detected by standard nuclei segmentation in the same tissue section. Most inferred cells lacked an associated nucleus (98.81%), and the mean cell area was only 4.0 μm^2, indicating that the inferred units largely corresponded to individual capture locations or very small local groups rather than whole cells. A single full run required 26.48 h in total across the nine tiles. These results are consistent with the original design of Baysor for molecule-resolved imaging-based spatial transcriptomics, in which inference relies on continuous molecule-level coordinates rather than the discrete capture locations used in Visium HD. Additional discussion of the likely reasons for this behavior is provided in subsection Implementation and Interpretation of Benchmarking Experiments .

SCS [9].

SCS also did not support Visium HD inputs directly and required substantial refactoring. To adapt SCS for H&E-based Visium HD data, we converted the histology image to grayscale, modified preprocessing to operate on pixel-based rather than spot-based distances, expanded the neighborhood range, removed the original stain-intensity cutoff used for background filtering, and redesigned patch generation to ensure full-canvas coverage of large tissue sections. Additional custom code was required to merge patch-wise outputs, reconstruct whole-section coordinates, export a standard cell-by-gene count matrix, and perform image-based quality control. Despite these efforts, SCS remained computationally demanding, requiring 100.51 h (4.12 days) to process the mouse brain dataset with GPU acceleration, and substantially longer without GPU support. The resulting segmentation was less concordant with nuclear morphology than SMURF, with 52.57% of detected segments lacking an associated nucleus and frequent multinuclear assignments (Supplementary Figure 2). Further discussion of these is provided in the "Implementation and Interpretation of Benchmarking Experiments" section.

Sparcle [10] and JSTA [11].

Sparcle and JSTA likewise did not natively support Visium HD and required extensive refactoring and method-specific adaptations for compatibility and scalability. These adaptations were substantial but were designed to preserve the original analytical logic of each method. Despite these efforts, both methods remained computationally impractical at this scale. For Sparcle, after 24.9 h of runtime, only 34 of the required 3,159,652 iterations had been completed, indicating an intractable runtime at Visium HD resolution. For JSTA, runtime on three representative 6×6 tiles ranged from 21.4 to 36.2 h per tile, corresponding to an estimated runtime of approximately 40 days 19 h for the full dataset. Owing to these implementation and compute constraints, neither Sparcle nor JSTA was included in our completed head-to-head benchmark. Additional details are provided in the "Implementation and Interpretation of Benchmarking Experiments" section below.

Nuclei segmentation.

Nuclei segmentation methods based on Stardist [14] and Cellpose [15, 16] are represented in the benchmarking described in main text (Figure 2) and in Supplementary Figures 1 and 2. The H&E-based "nuclei segmentation" benchmark on the mouse brain used Stardist-derived nuclei masks, whereas Cellpose-based nuclei segmentation was used for the DAPI-based benchmarking in Supplementary Figure 19. These methods provide relevant "nuclei segmentation" baselines for our benchmarking.

Summary

Taken together, our experiments show that, although methods developed for other ST platforms can in principle be adapted for use with Visium HD data, in practice, this is usually not feasible. Methods designed for molecule-resolved imaging platforms or alternative sequencing-based technologies required extensive data restructuring or code refactoring, and in several cases remained computationally impractical or produced biologically implausible segmentation when applied to Visium HD. By contrast, SMURF operates directly on Visium HD inputs without codebase modification and provides a favorable balance between computational tractability and segmentation quality. Across three runs, the mean runtime was 2.92 h for SMURF and 1.69 h for SMURF-lite, compared with 5.62 min for Bin2cell, 26.48 h for Baysor, and 100.51 h for SCS, whereas Sparcle and JSTA were not practically runnable at full Visium HD scale (Supplementary Table 1). Together with the extended benchmarks in mouse brain and mouse small intestine (Supplementary Figure 1 and 2), these results indicate that SMURF provides a robust and practically deployable framework for cell segmentation and transcript assignment in Visium HD datasets.

Implementation and Interpretation of Benchmarking Experiments

A key consideration in benchmarking on Visium HD is that many existing methods were originally developed for other spatial transcriptomics platforms and therefore operate under different assumptions. This note summarizes the implementation-level adaptations used to benchmark these methods on Visium HD data and outlines key considerations for interpreting the resulting comparisons. Because several comparator methods were developed for different input modalities, spatial resolutions, or segmentation assumptions, their performance on Visium HD should be interpreted in light of these platform-specific constraints.

Baysor [8].

Baysor was originally developed for cell segmentation in image-based spatial transcriptomics. The authors have discussed that Baysor is designed for single-molecule data rather than spot-based spatial transcriptomics on GitHub (https://github.com/kharchenkolab/Baysor/issues/5). That discussion centered on Visium data. Visium HD has a higher spatial resolution than Visium, but it is still a sequencing-based platform that captures UMI counts within discrete capture locations, so the same issues that led to the authors' response likely apply. We therefore evaluated Baysor on the Visium HD mouse brain dataset as a cross-platform benchmark.

Baysor requires single-molecule coordinates together with nuclei segmentation results as input. To make the analysis feasible on the large Visium HD dataset in terms of runtime and memory usage, we divided the whole slide into 3 × 3 patches and ran Baysor separately on each patch. We otherwise followed the default Baysor settings, while fixing `-s 3` to match the approximate size of a cell in 2 μm bins and using stricter filtering parameters. These stricter filters were introduced to handle sparse bin-level data, reduce noise-driven pseudo-cells, and improve segmentation stability and interpretability. We recorded the total runtime as the sum across the 9 patches. Because the full run was computationally expensive, we performed this computation once and did not repeat it three times to obtain an average runtime.

As quantified in the extended benchmarking results, Baysor did not produce biologically plausible segmentation on Visium HD data. The output effectively assigned most capture locations to unique inferred units. Across the 9 tiles, Baysor detected 2,293,396 cells, far exceeding the number of nuclei detected by standard nuclei segmentation in the same tissue section (57,583). This indicates that the inferred segmentation is not biologically plausible.

This behavior is consistent with Baysor's modeling assumptions. Baysor was developed for imaging-based spatial transcriptomics, in which each detected transcript is treated as an individual molecule with its own spatial coordinate. Its segmentation framework relies on molecule-level spatial organization, combining a Bayesian mixture model with a Markov Random Field (MRF) prior that encourages nearby molecules to share the same cell label. In addition, each cell is modeled as a smooth spatial distribution with a characteristic spatial scale.

In contrast, in Visium HD the basic observation unit is a 2 μm capture location rather than an individual molecule with a continuously varying coordinate. As a result, multiple transcripts can be aggregated at exactly the same discrete location, and the data no longer preserve the same molecule-level within-cell spatial variation that Baysor expects. Under this setting, local neighborhood structure becomes much less informative, weakening both the neighborhood-based representation and the MRF prior that Baysor uses to separate adjacent cells. Baysor also assumes

a relatively homogeneous cell body and a Gaussian-like cell shape approximation, assumptions that become less informative when the input is discretized into capture locations rather than resolved as molecule-level spatial positions. Consequently, the inference tends to assign very small local units, often corresponding to individual capture locations or only a few adjacent locations, rather than whole-cell boundaries.

Sparcle [10].

Sparcle is a probabilistic transcript-to-cell reassignment method that refines spot-level assignments by combining local neighborhood information with cluster-level expression models. It was originally developed for early image-based spatial transcriptomics platforms, where the number of detected genes and transcripts is relatively limited. Because Visium HD has a much denser sequencing-based output, we evaluated whether Sparcle could be applied to this type of data.

To make Sparcle runnable on Visium HD, we adapted the code accordingly. However, the original Sparcle package was not directly compatible with Visium HD data. Importantly, we did not modify the core logic of Sparcle itself. Instead, we only introduced implementation-level changes to improve robustness and compatibility for Visium HD-scale data. These changes included numerical stabilization for single-sample clusters, allowing singular covariance matrices during likelihood evaluation, improving coordinate and data-type safety, automatically creating output directories, and replacing deprecated `np.int` usage with the built-in `int`.

Even with these implementation-level adaptations, the direct application of Sparcle to full Visium HD data remained computationally infeasible. According to the run log, after approximately 24.9 hours, Sparcle had completed only 34 out of 3,159,652 iterations (approximately 0.0011% of the total), corresponding to roughly 2614 seconds per iteration. Extrapolating from this partial run, processing the full dataset would require on the order of hundreds of years. These results indicate that, even after implementation-level adaptation, Sparcle is not computationally practical for full-scale Visium HD analysis.

JSTA [11].

JSTA is a spatial transcriptomics analysis framework originally developed for image-based spatial methods with the aid of a single-cell reference. It segments pixels into cells by integrating nuclei coordinates, local spatial structure, and gene expression signals, and then uses a single-cell reference to iteratively refine cell boundaries and assign cell types.

For this evaluation, we selected a single-cell reference dataset from Ma et al. [17]. To make JSTA runnable on Visium HD data, we introduced several implementation-level fixes for scalability and robustness, without changing its overall analytical framework. In its original form, the JSTA package could not be run directly on Visium HD data. Specifically, we replaced the fixed marker-gene count with a dynamic value, modified `get_real_pixels(...)` to use the actually retained genes, added explicit sanity checks for gene/channel alignment, tightened the nuclei-assignment threshold, and added defensive logic when exporting AnnData, including automatic transpose fallback when cell/gene axes were swapped and a safe fallback for gene names. These changes were intended to prevent silent dimension mismatches and assignment errors on high-density data.

Because Visium HD is substantially larger than the image-based datasets for which JSTA was originally designed, we divided the dataset into 6 × 6 tiles and

paired each tile with the single-cell RNA-seq reference after reformatting the inputs to meet JSTA requirements. We randomly selected three tiles for these runs: tile07 required 130,228 seconds (36 h 10 min), tile25 required 86,463 seconds (24 h 01 min), and tile14 required 77,036 seconds (21 h 24 min). Extrapolating from this runtime, processing all 36 tiles in serial would require approximately 40 days 19 hours. These results indicate that, even after implementation-level adaptation, JSTA remains computationally impractical for full-scale Visium HD analysis.

SCS [9].

SCS is a segmentation package originally developed for subcellular sequencing-based methods such as Stereo-seq. It is a deep-learning-based pipeline that can merge multiple spots into a larger "spot profile" to reduce memory use and runtime. However, the package currently lacks a clear end-to-end tutorial and does not directly output a standard cell-by-gene count matrix. In a public GitHub discussion, users asked whether the authors would provide code to generate a cell-by-gene matrix, but no official workflow was provided (https://github.com/chenhcs/SCS/issues/10). In a separate public discussion, users also asked whether SCS could be applied to Visium HD, but no official workflow for this use case was available (https://github.com/chenhcs/SCS/issues/15).

We therefore performed an exploratory adapted implementation of SCS on Visium HD data, which required multiple implementation-level modifications. The SCS pipeline is heavily built on Spateo [18] and was originally designed for inputs that include DAPI and individual counts. Accordingly, the results reported here should be interpreted as reflecting an adapted exploratory implementation under H&E-based Visium HD constraints, rather than a native end-to-end SCS workflow on its intended input modality.

To enable this exploratory evaluation, we converted the H&E image into a grayscale representation and treated H&E pixels as the basic unit. During preprocessing, we expanded the neighborhood range because we were using pixel distance rather than spot distance, removed the stain-intensity cutoff originally used for background filtering, and retained the distance-to-nucleus criterion for H&E adaptation. During patch generation, we replaced the original max–min extent strategy with full-canvas coverage to avoid missing edge regions in large Visium HD sections.

During experiments, we encountered three major practical issues. First, we observed unstable and non-monotonic memory behavior. Using the authors' code with their patching strategy, memory usage increased with iteration count and eventually caused out-of-memory failures on a 256 GB machine. However, restarting from the failure point could sometimes proceed successfully, suggesting that the observed memory growth may reflect accumulation or caching effects rather than only the nominal data size. In total, the run required 100.51 h (4.12 days) with GPU acceleration.

Second, SCS performs alignment separately for each patch and does not provide an official procedure to merge patches into a globally consistent coordinate system. This makes the reconstructed full-tissue result potentially inconsistent across patches. We attempted to manually re-merge and re-align patches to mitigate this issue, but this required substantial additional engineering and remains a source of uncertainty.

Third, SCS does not provide code to export a standard cell-by-gene matrix and also lacks built-in visualization for qualitative comparison against DAPI or H&E. We

therefore implemented custom code to generate the required outputs and to perform image-based sanity checks.

Overall, while SCS is potentially relevant for high-resolution spatial transcriptomics, the current software interface and patch-level workflow make it difficult to apply reliably to Visium HD without extensive additional development and without clear guidance on producing standard downstream-ready outputs.

Comparison of downstream spatial domain identification across segmentation methods

To examine how segmentation strategy affects downstream spatial analysis, we compared spatial domain identification in the mouse brain dataset using a standard Squidpy workflow [19]. For each method, we used the corresponding reconstructed cell- or spot-level expression matrix together with spatial coordinates and applied neighborhood graph construction, dimensionality reduction, and Leiden-based domain identification within the same general analytical framework.

We focused on the hippocampal region because it contains anatomically adjacent yet transcriptionally distinct substructures, providing a useful test of whether biologically meaningful spatial organization is preserved after transcript assignment. In this region, SMURF produced spatial domains that more closely matched known hippocampal anatomy than the other methods (Supplementary Figure 17a-f). In particular, SMURF showed clearer subdivision of the hippocampal pyramidal layer and began to separate the CA1/2 region from CA3, whereas nuclei-only segmentation, 8 μm binning, Loupe Browser, and Bin2cell recovered broader domains and showed less fine-grained anatomical separation. Across these alternative approaches, neighboring hippocampal substructures were more frequently merged into larger regions.

We used the Adjusted Rand Index (ARI) to quantify the agreement between hippocampal domains determined based on histology and the ST cluster assignments [20, 21]. We manually annotated 4 hippocampal subfields (CA1, CA2, CA3, and DG) based on the histology and canonical marker-gene patterns (Supplementary Figure 17g,h). Using this reference, SMURF achieved the highest ARI among all five methods (Supplementary Figure 17i), consistent with the qualitative observation that its inferred domains more closely matched known hippocampal substructures.

These results suggest that SMURF better preserves biologically meaningful local spatial organization for downstream domain identification. More broadly, they indicate that improved transcript assignment at single-cell resolution can benefit not only cell-level reconstruction but also higher-order analyses that depend on spatial neighborhood structure.

Lite (Non-GPU) version of SMURF and example results

The deconvolution module of SMURF relies on a PyTorch-based deep learning implementation that demands substantial GPU resources. To offer a more accessible option for all users, we introduce a lite version which does not require access to GPUs. The lite version streamlines the algorithm by deconvolving shared spots only using information on the spot's distance from the center of its corresponding nucleus. We applied this lite version to a public mouse embryo dataset (H&E staining) [22] and human tonsil dataset (IF staining) [23] to demonstrate its effectiveness.

For the mouse embryo example (Supplementary Figure 18) and the human tonsil example (Supplementary Figure 19), we visualized how SMURF-lite assigned capture spots to nuclei in three distinct sections, and compared those results to assignments obtained using the 8 μm method, Bin2cell, and nuclei segmentation. While we evaluated Space Ranger results on the embryo datasets, we could not consider that method on the human tonsil dataset because Space Ranger does not accept DAPI images as input for this analysis. SMURF-lite reliably achieves single-cell resolution by assigning transcripts from overlapping and non-overlapping capture spots to cells, outperforming the 8 μm method, Bin2cell, nuclei segmentation, and Space Ranger, especially in densely populated regions.

Concluding that the 8 μm method, Bin2cell, and Space Ranger do not reliably achieve single-cell resolution with Visium HD data, we next compared SMURF-lite directly to the nuclei segmentation method, analogous to the comparison shown in Figure 2d of the main text. Since SMURF-lite includes capture spots that do not overlap nuclei, we hypothesized that it would yield larger median cell sizes and higher transcript counts per cell. Indeed, when analyzing both mouse embryo and human tonsil Visium HD data with SMURF-lite or nuclei segmentation (Supplementary Figure 18d and Supplementary Figure 19d), SMURF-lite produced a 7.29-fold increase in median UMIs per cell (714 vs. 98) and a 7.03-fold increase in median cell area (56.21 μm^2 vs. 8.00 μm^2) for the mouse embryo data. For the human tonsil data, SMURF-lite provided a 1.84-fold increase in median UMIs per cell (204 vs. 111) and a 1.98-fold increase in median cell area (47.49 μm^2 vs. 24.00 μm^2). Both methods identified comparable numbers of cells (Supplementary Figure 18d and Supplementary Figure 19d, middle), consistent with their shared reliance on segmented nuclei as the basis for defining cells. Collectively, these data indicate that SMURF-lite assigns substantially more transcripts to each cell than the nuclei segmentation method by incorporating capture spots beneath cell bodies, thus leading to larger overall cell sizes.

In total, SMURF-lite surpasses the 8 μm method, Bin2cell, nuclei segmentation, and Space Ranger by more accurately assigning transcripts to cells at single-cell resolution, thereby offering reliable spatial single-cell results through SMURF for researchers lacking GPU resources.

SMURF vs. SMURF-lite: evaluating the contribution of the deep learning module

SMURF provides two operating modes that share the same overall soft-segmentation framework but differ in how transcripts from shared spots are assigned. In the full SMURF workflow, transcripts from spots shared between neighboring cells of the same type are partitioned using the deep learning module, whereas in SMURF-lite they are assigned using a spatial distance-weighted multinomial distribution. For shared spots between cells of different types, the same canonical deconvolution strategy is used in both modes.

To quantify the effect of these different assignment strategies, we compared the full SMURF workflow with SMURF-lite using the cosine similarity between each reconstructed cell and the mean expression profile of its assigned cluster. Higher cosine similarity indicates that the assigned transcripts are more consistent with the expression profile of the inferred parent cell type. In both the mouse brain and mouse small intestine datasets, cells reconstructed by the full SMURF workflow showed slightly but statistically significantly higher cosine similarity than cells reconstructed by SMURF-lite (Supplementary Figure 20), indicating that the deep learning module improves transcript assignment accuracy beyond the soft-segmentation framework alone.

This comparison demonstrates that deep learning based deconvolution of shared spots between neighboring cells of similar type provides measurable benefit relative to a simpler spatial distance-weighted random assignment strategy. Although neighboring cells often have similar expression profiles, the deep learning module improved consistency with the cluster-level expression structure, supporting its use in the full SMURF pipeline. A relatively small fraction of total transcripts is impacted because these methods only differ in how they handle spots shared by cells of the same cell type, so it is not surprising that the absolute difference between SMURF and SMURF-lite is modest. We recommend full SMURF for users with access to GPUs to achieve the highest performance. For those users without access to GPUs, it is reassuring that the SMURF-lite approach is a low-compute option that still provides best-in-class segmentation (Supplementary Figures 18 and 19) along with advanced unrolling strategies to enhance biological interpretation.

Sensitivity analysis for the minimum overlap fraction used in shared-spot initialization

To initialize transcript assignment for shared spots, SMURF uses a minimum overlap fraction threshold to define candidate cell-spot relationships at the start of the optimization. Because this parameter affects only the initialization step, whereas final transcript assignment is refined by the downstream expectation-maximization procedure, we expected SMURF to be relatively insensitive to modest variation in this setting.

To assess the sensitivity of SMURF to the minimum overlap fraction used for initializing shared-spot assignment, we repeated the analysis using thresholds of 1/5, 1/6, and 1/7. Across these settings, the resulting low-dimensional structure and cluster assignments remained broadly consistent (Supplementary Figure 21). To quantify this, we compared Leiden cluster assignments among cells shared across runs ($n = 56{,}774$ common cells). Pairwise normalized mutual information values were 0.520 for 1/5 versus 1/6, 0.505 for 1/5 versus 1/7, and 0.550 for 1/6 versus 1/7, indicating similar overall clustering structure across nearby parameter choices. In addition, the overlap of retained cells across runs was very high, with the shared-cell set comprising 99.09% of cells in the 1/5 run, 99.84% in the 1/6 run, and 99.80% in the 1/7 run. These results indicate that SMURF is not highly sensitive to modest variation in this initialization parameter.

Assessment of cross-cell-type marker contamination after SMURF expansion

A general limitation of sequencing-based spatial transcriptomics is that transcripts may diffuse into nearby locations, producing local bleedover or “spot swapping” between neighboring measurement units [24, 25]. To determine whether SMURF-based cell expansion increases erroneous assignment of transcripts across cell types relative to rigid nuclei segmentation, we evaluated marker specificity in the mouse brain dataset. Cell types were assigned using the Allen Brain Atlas MapMyCells tool [26, 27], and we compared the expression of canonical marker genes across matched cell populations defined by nuclei-only segmentation or SMURF segmentation. As expected, SMURF-assigned cells showed higher overall marker-gene detection than nuclei-only segmentation, consistent with an increased sensitivity due to broader transcript recovery. However, SMURF segmentation did not show an increase in cross-cell-type marker contamination. Instead, across the four neuronal cell types examined, SMURF-assigned cells showed fewer instances of erroneous marker-gene detection than nuclei-only segmentation (Supplementary Figure 22).

These results suggest that the increased transcript recovery obtained by SMURF improves effective cell-level representation without increasing cross-cell-type contamination in this setting. One possible interpretation is that the higher sensitivity of SMURF leads to more accurate clustering and cell-type assignment, thereby improving both marker detection and marker specificity. Thus, in this empirical comparison, SMURF achieved a more favorable balance between sensitivity and specificity than rigid nuclei segmentation alone.

Spatial scRNA-seq Mirrors Single-Cell Statistics: Gene-Wise NB Fits SMURF Data

Bulk RNA-seq analysis aggregates gene expression measurements from large pools of cells, providing a population-level snapshot of transcriptional activity within a sample [28]. Many computational tools for analyzing bulk RNA-seq data are built on statistical distributions that model the discrete and often overdispersed count data generated in these experiments. The Poisson model assumes that the variance equals the mean; however, this assumption may prove inadequate when overdispersion is present. The Negative Binomial (NB) model overcomes this limitation by incorporating an additional dispersion parameter, which quantifies the extent to which the variance exceeds the mean [29, 30, 31].

In contrast to bulk RNA-seq, scRNA-seq data typically contain a high degree of observed zero counts (commonly referred to as "dropouts"). Zero counts arise from both biological and technical factors. When excessive zero counts occur, the zero-inflated negative binomial (ZINB) model can be employed. This model effectively separates the NB component from the processes generating zero counts, offering a more flexible fit to the heterogeneous count distributions observed in single-cell transcriptomic data [32, 33]. However, recent studies on UMI-based protocols have demonstrated that many zero counts can be adequately modeled using simpler Poisson or NB distributions after accounting for cell-type heterogeneity, thereby reducing the need for additional zero-inflation components [34, 35, 36].

These insights have spurred the development of a wide range of computational methods for both bulk and single-cell RNA-seq analyses. For instance, tools such as edgeR [30] and DESeq2 [31] employ NB models to account for biological variability. Additionally, scvi is a deep generative framework that allows users to assume that counts follow various distributions — including the NB, ZINB, Poisson, or even the normal distribution — to capture the inherent variability in single-cell transcriptomic data [37].

The observed counts in cells from spatial transcriptomics data are frequently even lower than what is observed in dissociation-based scRNA-seq. For example, SMURF identified a median of 435 UMIs per cell in the mouse brain, and only 182 UMIs per cell in the mouse small intestine. In contrast, current scRNA-seq protocols can capture more than 7,000 UMIs per cell when deeply sequenced [38]. Despite the exploding interest in spatial transcriptomics methods, we do not know the best model to fit these datasets that accounts for the high frequency of zero counts.

To evaluate the distribution of SMURF-based spatial single-cell data, we began by examining dispersion under the assumption that cells within the same cluster are homogeneous. We then analyzed different cell types separately, focusing on those with more than 1,000 cells (mouse brain) and those with more than 10,000 cells (mouse small intestine). Focusing on genes with more than 100 total counts across all cells, we plotted the mean against the variance (see Figures 4 and 5). The results show that the variance consistently exceeds the mean, indicating marked overdispersion, particularly for genes with higher mean expression.

Subsequently, we examined the underlying sources of low counts in spatial single-cell data to assess which modeling strategies best account for the high prevalence of zeros. We analyzed the data using both Poisson and NB (with gene-wise dispersion) distributions to estimate the expected probability of zeros and compared these estimates with the observed zero frequencies (see Figures 6 and 7). For most genes

and cell types, the observed percentage of zeros is higher than that predicted by the Poisson model yet closely aligns with the predictions of the NB model.

For a quantitative assessment, we applied the Poisson model, the NB model with common dispersion, the NB model with gene-wise dispersion, and the ZINB model. For each dataset, we computed the number and percentage of genes that deviate from the expected fraction of zero counts by more than 1, 5, 10, and 20 percentage points. Our findings reveal that both the Poisson model and the NB model with common dispersion deviate considerably from the empirical data, whereas the NB model with gene-wise dispersion aligns closely with the observed values. Moreover, the ZINB model performs nearly identically to the NB model with gene-specific dispersion, indicating that once gene-level dispersion is modeled, additional zero-inflation offers little further improvement.

In summary, our analysis demonstrates that the NB model, particularly when implemented with gene-wise dispersion, more accurately captures the variability and zero-count distributions observed in SMURF-based spatial single-cell datasets. As a result, algorithms and pipelines originally developed for single-cell RNA-seq can be effectively applied to these spatial single-cell data. These analyses further highlight a general limitation of high-resolution spatial transcriptomics: reconstructed single-cell profiles remain sparse because only a limited number of transcripts are captured per cell. As a result, sensitivity for lowly expressed genes is reduced, zero counts and dropout remain common, and subtle differences in cell state or gene expression may be harder to resolve robustly. Although SMURF improves effective cell-level representation by incorporating transcripts beyond the nucleus and leveraging spatial continuity, it cannot recover molecular signals that were not captured experimentally. Therefore, downstream biological interpretation should be made with appropriate caution, particularly for low-abundance genes and subtle intermediate states.

Normalization artifact in the 8 μm method

We noticed an artifact specific to the 8 μm method. Because of the way this method normalizes count data, if the data contains spatial regions where few transcripts are captured, there is a severe data imbalance that skews the expression values for some genes. For example, Slc1a2 is an important gene primarily expressed in the CA3 subregion of the mouse hippocampus [39, 40]. However, 8 μm normalization of count data for this gene is discordant with ISH data from the Allen Brain Atlas. In contrast, the single-cell data generated by SMURF is much more concordant with the ISH data (Supplementary Figure 3a). This improvement arises because SMURF reallocates transcripts to individual cells based on spatial continuity. This corrects the undersampling artifacts that plague sparsely populated regions.

Identification of Highly Zonated Genes

For each zone (1–6), we first restricted candidates to genes detected in more than 100 cells within the corresponding dataset. We then used Scanpy differential-expression results to call zone-enriched genes under zone-specific thresholds on $\log_2$ fold-change (LFC) and adjusted p-value. To ensure high specificity, we excluded genes significantly enriched in other epithelial zones and in non-epithelial compartments (smooth muscle, immune, fibroblasts, endothelial), as well as Goblet, Tuft, Peyer's patch, and M cell-related set, using the exact criteria listed below.

Zone 1:

Selection: LFC > 1.8 and adjusted $p < 10^{-40}$. Exclusions: genes enriched in Zone 2 (LFC > 3, $p < 10^{-50}$), Zone 3 (LFC > 0, $p < 10^{-50}$), or Zone 4 (LFC > 0, $p < 10^{-50}$); in smooth muscle (LFC > 0, $p < 10^{-30}$), immune (LFC > 0, $p < 10^{-30}$), Goblet (LFC > 0, $p < 10^{-30}$), Peyer's patch (LFC > 0, $p < 10^{-30}$), M cells-related (LFC > 0, $p < 10^{-30}$), Tuft (LFC > 0, $p < 10^{-30}$), Endothelial (LFC > 0, $p < 10^{-30}$), or Fibroblasts (LFC > 2.8, $p < 10^{-40}$).

Zone 2:

Selection: LFC > 2.0 and adjusted $p < 10^{-50}$. Exclusions: genes enriched in Zone 1 (LFC > 0, $p < 10^{-100}$), and in smooth muscle, immune, Goblet, Peyer's patch, Enterocyte + M cells, Tuft, Endothelial, or Fibroblasts (all with LFC > 0, $p < 10^{-100}$).

Zone 3:

Selection: LFC > 1.4 and adjusted $p < 10^{-30}$. Exclusions: genes enriched in Zone 2, Zone 6, or Zone 5 (all with LFC > 0, $p < 10^{-100}$).

Zone 4:

Selection: LFC > 1.5 and adjusted $p < 10^{-10}$. Exclusions: genes enriched in Zone 6 or Zone 3 (both with LFC > 0, $p < 10^{-100}$).

Zone 5:

Selection: LFC > 1.5 and adjusted $p < 10^{-10}$. Exclusions: genes enriched in Zone 6, Zone 3, or Zone 4 (all with LFC > 0, $p < 10^{-100}$), and in the immune dataset (LFC > 0, $p < 10^{-100}$).

Zone 6:

Selection: LFC > 2.0 and adjusted $p < 10^{-20}$. Exclusions: genes enriched in Zone 4, Zone 3, or Zone 5 (all with LFC > 0, $p < 10^{-50}$), and in the immune dataset (LFC > 0, $p < 10^{-50}$).

Sensitivity of intestinal zonation results to the number of spatial zones

To assess whether the intestinal zonation results depended strongly on our choice of six discrete zones, we repeated the analysis using alternative discretizations of the crypt-villus axis into five and seven zones. We selected six zones in the main analysis to facilitate comparison with established intestinal zonation studies, particularly the framework of Moor et al [2], which partitions the villus into five sub-zones in addition to the crypt. Across the 5-, 6-, and 7-zone analyses, the overall gene-expression trends remained highly similar, and the major biological conclusions were unchanged (Supplementary Figures 23 and 24). All analyses were performed using the same methods and parameters as in the main text, except for the number of zones. These results indicate that the zonation patterns identified by SMURF are robust to modest variation in the number of spatial bins used for pseudo-bulk analysis.

Comparison of SMURF unrolling with existing Swiss-roll unrolling methods

To further benchmark SMURF against existing Swiss-roll unrolling methods, we examined Semla [41] and IntestLine [42]. These methods were developed primarily for spot- or cell-based datasets and rely on user-guided geometric operations, such as manual graph editing or manual specification of a base layer. By contrast, SMURF is designed for high-resolution spatial data and infers unrolling coordinates from cell organization and clustering while preserving physical distance information. We attempted to apply Semla to our Visium HD dataset; however, the software's graphical interface was unresponsive to the manual inputs required to delineate the base layer, preventing us from successfully executing it in our local computing environment. We therefore performed a direct comparison between SMURF and IntestLine on the same mouse small intestine section. Because manual specification of the x-axis and center point is required in the original IntestLine workflow, these inputs were provided using the same tissue geometry as that used for SMURF. This ensured that the comparison reflected differences in method performance rather than differences in the initial geometric reference. No modifications were made to the IntestLine algorithm itself.

IntestLine was substantially slower on more than 400,000 cells (3 h 45 min versus 35 min) and produced x- and y-axis assignments that were less consistent with the expected tissue geometry (Supplementary Figure 25). For example, IntestLine often assigned villus cells to the wrong muscle layer, resulting in low y-axis values for villus cells that would be expected to have high values. In panel d-f, this behavior is visible in regions where red villus cells appear adjacent to red muscle cells despite being separated by luminal space. Together, these results suggest that SMURF is better suited for dense, high-resolution spatial datasets.

Crypt–villus boundary analysis in the mouse ileum

Supplementary Figure 26 shows the inferred crypt-villus boundary traced along the entire length of the Swiss roll. To assess the plausibility of this boundary, we quantified the overall distribution of cell types within each compartment from the intact villi. As expected, epithelial stem/Paneth cells and transit-amplifying (TA) cells were detected almost exclusively in the crypt compartment, consistent with prior biological expectations (Supplementary Table 7). This suggests that SMURF is accurately unrolling the intestine, even when considered at a fine tissue substructure level. One limitation of this analysis is that there is currently no gold-standard reference for crypt and villus cellular composition in the mouse ileum. More rigorous benchmarking of the inferred crypt–villus boundary will require future generation of such a reference dataset.

Comparison of cell-type annotation across transcript-assignment methods

To further assess how SMURF's transcript-assignment strategy affects downstream cell-type annotation, we compared clustering results across methods using the mouse small intestine dataset. This analysis extends the main-text comparison between SMURF and nuclei segmentation to now include Bin2cell and Space Ranger.

Because Bin2cell does not provide clustering information directly, we performed downstream clustering on the Bin2cell-derived cell-by-gene matrix using the standard workflow of log-normalization, principal component analysis, neighborhood graph construction, and unsupervised clustering. For Space Ranger, we used the clustering output provided directly by the 10x Genomics pipeline. We then compared the expression of canonical marker genes for enterocytes, tuft cells, enteroendocrine cells, and smooth muscle cells across clusters from each method (Supplementary Figure 8).

Enterocyte and smooth-muscle marker signatures could be detected with all three methods, although cluster separation was less distinct than in SMURF. In contrast, tuft-cell and enteroendocrine-cell markers were not cleanly detected in any cluster in the nuclei segmentation, Bin2cell, or Space Ranger analysis. In contrast, these rarer epithelial populations were identified by SMURF. These results demonstrate that these other methods have limited sensitivity for rare or morphologically irregular cell types. Overall, this analysis supports that improved transcript recovery by SMURF facilitates downstream cell-type identification, particularly for rarer epithelial populations.

Comparison of SMURF-derived cell-type abundance with reference atlases

To assess whether SMURF accurately recovers cell-type composition, we compared SMURF-derived cell-type abundances with available reference atlases.

For the mouse brain, a comprehensive reference atlas with abundance information is available. We therefore compared the abundance of major cell types detected by SMURF with the Mouse Brain Atlas using MapMyCells [26]. As shown in Supplementary Figure 3b, the relative abundance of major cell types was highly similar between SMURF and the reference scRNA-seq atlas.

For the mouse intestine, Supplementary Table 3 summarizes the overall abundance of major cell types detected in the SMURF mouse intestine analysis. To our knowledge, no single reference atlas currently provides abundance estimates across all major epithelial and non-epithelial cell types in the intestine. Published mouse intestine datasets differ in anatomical sampling, tissue processing, dissociation strategy, and cellular focus. In particular, epithelial, stromal, and immune compartments are often profiled using distinct dissociation and library-preparation protocols, limiting direct comparison of their relative abundances across studies.

We therefore focused validation on the epithelial compartment, which is the most abundant and best characterized compartment in our mouse intestine dataset. We collated epithelial cell-type abundance estimates from three published studies: Haber et al. [12], Zwick et al. [1], and Xu et al [13]. Haber et al. profiled the small intestine using a crypt-enriched isolation strategy, Zwick et al. performed 10x scRNA-seq using a total epithelial isolation strategy across the small intestine, and Xu et al. analyzed the mouse ileum using a custom MERFISH probe panel. We extracted the relative abundance of epithelial cell types from the data presented in the paper or from the author annotated scRNA-seq data objects. Supplementary Table 2 compares the relative abundance of major epithelial cell types detected by SMURF with the three reference studies. Despite differences in experimental design, anatomical coverage, and computational analysis, the epithelial cell-type composition inferred by SMURF was broadly concordant with these reference datasets.

Together, these results support that SMURF recovers expected cellular composition in the mouse brain and intestine.

Transcription Factor-Related Results

Here we show the results from SCENIC analysis [43]. For each regulon, we calculated the average activity score in each zone and retained only regulons whose mean activity score across all zones exceeded 0.05.

Demonstration of the SMURF unrolling framework on spatial proteomics data

We sought to evaluate whether SMURF's unrolling algorithm could be applied to non-transcriptomic spatial data. We identified a published mouse intestine spatial proteomics dataset generated using CODEX [44]. We used the publicly available data provided with that study [42], including cell locations and antibody-intensity measurements. The paper provided public data for 10,000 of the 150,793 total cells in the form of cell locations and antibody staining intensity. The authors manually traced the base of the intestine. Using the authors' reported trace as the pseudo-x axis base in SMURF, we were able to assign x-y coordinates to the other cells (Supplementary Figure 16). Although the available data was sparse, the cells in the image were placed on appropriate x and y coordinates reflecting their proximal-distal and crypt-villus location. These results suggest that the unrolling framework may be adaptable to protein-based spatial imaging data.

References

[1] Rachel K Zwick, Petr Kasparek, Brisa Palikuqi, Sara Viragova, Laura Weichselbaum, Christopher S McGinnis, Kara L McKinley, Asoka Rathnayake, Dedeepya Vaka, Vinh Nguyen, et al. Epithelial zonation along the mouse and human small intestine defines five discrete metabolic domains. *Nature Cell Biology*, 26(2):250–262, 2024.

[2] Andreas E Moor, Yotam Harnik, Shani Ben-Moshe, Efi E Massasa, Milena Rozenberg, Raya Eilam, Keren Bahar Halpern, and Shalev Itzkovitz. Spatial reconstruction of single enterocytes uncovers broad zonation along the intestinal villus axis. *Cell*, 175(4):1156–1167, 2018.

[3] Nikolaos Tzakis and Matthew R Holahan. Social memory and the role of the hippocampal ca2 region. *Frontiers in behavioral neuroscience*, 13:233, 2019.

[4] Kristen M Seiler, Sarah E Waye, Wenjun Kong, Kenji Kamimoto, Adam Bajinting, William H Goo, Emily J Onufer, Cathleen Courtney, Jun Guo, Brad W Warner, et al. Single-cell analysis reveals regional reprogramming during adaptation to massive small bowel resection in mice. *Cellular and molecular gastroenterology and hepatology*, 8(3):407–426, 2019.

[5] 10x Genomics. Release notes for space ranger. https://www.10xgenomics.com/support/software/space-ranger/latest/release-notes/release-notes-for-SR, 2025. Accessed March 12, 2026. Version 4.0.1; includes Visium HD support and nucleus/cell segmentation.

[6] 10x Genomics. Space ranger downloads. https://www.10xgenomics.com/support/software/space-ranger/downloads, 2025. Accessed March 12, 2026. Space Ranger v4.0.1.

[7] Teichlab. bin2cell. https://github.com/Teichlab/bin2cell, n.d. Accessed: September 14, 2024.

[8] Viktor Petukhov, Rosalind J Xu, Ruslan A Soldatov, Paolo Cadinu, Konstantin Khodosevich, Jeffrey R Moffitt, and Peter V Kharchenko. Cell segmentation in imaging-based spatial transcriptomics. *Nature biotechnology*, 40(3):345–354, 2022.

[9] Hao Chen, Dongshunyi Li, and Ziv Bar-Joseph. Scs: cell segmentation for high-resolution spatial transcriptomics. *Nature methods*, 20(8):1237–1243, 2023.

[10] Sandhya Prabhakaran. Sparcle: assigning transcripts to cells in multiplexed images. *Bioinformatics advances*, 2(1):vbac048, 2022.

[11] Russell Littman, Zachary Hemminger, Robert Foreman, Douglas Arneson, Guanglin Zhang, Fernando Gómez-Pinilla, Xia Yang, and Roy Wollman. Joint cell segmentation and cell type annotation for spatial transcriptomics. *Molecular systems biology*, 17(6):MSB202010108, 2021.

[12] Allison L. Haber, Merav Biton, Noa Rogel, Robert H. Herbst, Kedar Shekhar, Chris Smillie, Gordon Burgin, Terrence M. Delorey, Martin R. Howitt, Yaniv Katz, et al. A single-cell survey of the small intestinal epithelium. *Nature*, 551(7680):333–339, 2017.

[13] Rosalind J Xu, Hao Zhang, Paolo Cadinu, Phillip B Nicol, Uli S Herrmann, Tyrone Lee, Ludwig Geistlinger, Rafael A Irizarry, and Jeffrey R Moffitt. An image-based transcriptomics atlas reveals the regional and microbiota-dependent molecular, cellular, and spatial structure of the murine gut. *Cell host & microbe*, 2025.

[14] Martin Weigert and Uwe Schmidt. Nuclei instance segmentation and classification in histopathology images with stardist. In *The IEEE International*

Symposium on Biomedical Imaging Challenges (ISBIC), 2022.

[15] Carsen Stringer, Tim Wang, Michalis Michaelos, and Marius Pachitariu. Cellpose: a generalist algorithm for cellular segmentation. *Nature methods*, 18(1):100–106, 2021.

[16] Carsen Stringer and Marius Pachitariu. Cellpose3: one-click image restoration for improved cellular segmentation. *Nature Methods*, pages 1–8, 2025.

[17] Ying Ma and Xiang Zhou. Spatially informed cell-type deconvolution for spatial transcriptomics. *Nature biotechnology*, 40(9):1349–1359, 2022.

[18] Xiaojie Qiu, Daniel Y Zhu, Yifan Lu, Jiajun Yao, Zehua Jing, Kyung Hoi Min, Mengnan Cheng, Hailin Pan, Lulu Zuo, Samuel King, et al. Spatiotemporal modeling of molecular holograms. *Cell*, 187(26):7351–7373, 2024.

[19] Giovanni Palla, Hannah Spitzer, Michal Klein, David Fischer, Anna Christina Schaar, Louis Benedikt Kuemmerle, Sergei Rybakov, Ignacio L Ibarra, Olle Holmberg, Isaac Virshup, et al. Squidpy: a scalable framework for spatial omics analysis. *Nature methods*, 19(2):171–178, 2022.

[20] Lawrence Hubert and Phipps Arabie. Comparing partitions. *Journal of classification*, 2(1):193–218, 1985.

[21] Kangning Dong and Shihua Zhang. Deciphering spatial domains from spatially resolved transcriptomics with an adaptive graph attention auto-encoder. *Nature communications*, 13(1):1739, 2022.

[22] 10x Genomics. Visium hd cytassist gene expression libraries of mouse embryo. https://www.10xgenomics.com/datasets/visium-hd-cytassist-gene-expression-libraries-of-mouse-embryo. Accessed: 28 February 2025.

[23] 10x Genomics. Visium hd human tonsil fresh frozen if. https://cf.10xgenomics.com/samples/spatial-exp/3.1.1/Visium_HD_Human_Tonsil_Fresh_Frozen_IF/Visium_HD_Human_Tonsil_Fresh_Frozen_IF_web_summary.html. Dataset version 3.1.1. Accessed: 28 February 2025.

[24] Zijian Ni, Aman Prasad, Shuyang Chen, Richard B Halberg, Lisa M Arkin, Beth A Drolet, Michael A Newton, and Christina Kendziorski. Spotclean adjusts for spot swapping in spatial transcriptomics data. *Nature Communications*, 13(1):2971, 2022.

[25] 10x Genomics. Your introduction to visium hd: Spatial biology in high definition. https://www.10xgenomics.com/blog/your-introduction-to-visium-hd-spatial-biology-in-high-definition, April 2024. Accessed: 2026-03-17.

[26] Allen Institute for Brain Science. Mapmycells (rrid:scr_024672). https://portal.brain-map.org/atlases-and-data/bkp/mapmycells. Accessed: 16 February 2025.

[27] Zizhen Yao, Cindy TJ van Velthoven, Michael Kunst, Meng Zhang, Delissa McMillen, Changkyu Lee, Won Jung, Jeff Goldy, Aliya Abdelhak, Matthew Aitken, et al. A high-resolution transcriptomic and spatial atlas of cell types in the whole mouse brain. *Nature*, 624(7991):317–332, 2023.

[28] Zhong Wang, Mark Gerstein, and Michael Snyder. Rna-seq: a revolutionary tool for transcriptomics. *Nature reviews genetics*, 10(1):57–63, 2009.

[29] Simon Anders and Wolfgang Huber. Differential expression analysis for sequence count data. *Nature Precedings*, pages 1–1, 2010.

[30] Mark D Robinson, Davis J McCarthy, and Gordon K Smyth. edger: a bioconductor package for differential expression analysis of digital gene expression data. *bioinformatics*, 26(1):139–140, 2010.

[31] Michael I Love, Wolfgang Huber, and Simon Anders. Moderated estimation of fold change and dispersion for rna-seq data with deseq2. *Genome biology*, 15:1–21, 2014.

[32] Peter V Kharchenko, Lev Silberstein, and David T Scadden. Bayesian approach to single-cell differential expression analysis. *Nature methods*, 11(7):740–742, 2014.

[33] Catalina A Vallejos, Davide Risso, Antonio Scialdone, Sandrine Dudoit, and John C Marioni. Normalizing single-cell rna sequencing data: challenges and opportunities. *Nature methods*, 14(6):565–571, 2017.

[34] Valentine Svensson. Droplet scrna-seq is not zero-inflated. *Nature Biotechnology*, 38(2):147–150, 2020.

[35] Tae Hyun Kim, Xiang Zhou, and Mengjie Chen. Demystifying "drop-outs" in single-cell umi data. *Genome biology*, 21(1):196, 2020.

[36] Yue Pan, Justin T Landis, Razia Moorad, Di Wu, JS Marron, and Dirk P Dittmer. The poisson distribution model fits umi-based single-cell rna-sequencing data. *BMC bioinformatics*, 24(1):256, 2023.

[37] Romain Lopez, Jeffrey Regier, Michael B Cole, Michael I Jordan, and Nir Yosef. Deep generative modeling for single-cell transcriptomics. *Nature methods*, 15(12):1053–1058, 2018.

[38] 10x Genomics. 10x genomics dataset repository. https://www.10xgenomics.com/datasets, 2025. Collection of publicly available single-cell and spatial genomics datasets from 10x Genomics.

[39] Ke Cheng, Chong Chen, Qinji Zhou, Xiangyu Chen, and Peng Xie. Deficit of neuronal eaat2 impairs hippocampus ca3 neuron's activity and may induce depressive like behaviors. *Journal of Advanced Research*, 2025.

[40] Allen Institute for Brain Science. Allen mouse brain atlas [dataset]. http://mouse.brain-map.org, 2004. Accessed: 2025-03-24.

[41] Ludvig Larsson, Lovisa Franzén, Patrik L Ståhl, and Joakim Lundeberg. Semla: a versatile toolkit for spatially resolved transcriptomics analysis and visualization. *Bioinformatics*, 39(10):btad626, 2023.

[42] Altay Yuzeir, David Alejandro Bejarano, Stephan Grein, Jan Hasenauer, Andreas Schlitzer, and Jiangyan Yu. Intestline: a shiny-based application to map the rolled intestinal tissue onto a line. *Bioinformatics*, 39(4):btad140, 2023.

[43] Sara Aibar, Carlos B. González-Blas, Thomas Moerman, Vân A. Huynh-Thu, Hana Imrichova, Geert Hulselmans, Florian Rambow, José C. Marine, Pierre Geurts, and Stein Aerts. SCENIC: single-cell regulatory network inference and clustering. *Nature Methods*, 14(11):1083–1086, 2017.

[44] Yury Goltsev, Nikolay Samusik, Julia Kennedy-Darling, Salil Bhate, Matthew Hale, Gustavo Vazquez, Sarah Black, and Garry P Nolan. Deep profiling of mouse splenic architecture with codex multiplexed imaging. *Cell*, 174(4):968–981, 2018.
