# Supplementary material for: SMURF: soft-segmentation for single-cell reconstruction and topological analysis of spatial transcriptomic data
Source: Nat Commun. 2026 Jun 26;17:7990. doi: 10.1038/s41467-026-74464-4 (PMC13447825; doi:10.1038/s41467-026-74464-4)
Supplement: Supplementary file 2 [file 41467_2026_74464_MOESM2_ESM.pdf]

Description of Additional Supplementary Files

File Name: Supplementary Data 1

Description:

Significantly zonated genes along the crypt-villus axis of the mouse ileum

Paneth and stem cells, TA cells, and enterocytes were selected from intact villi and divided into six zones according to their normalized corrected y-axis position. Only genes with counts greater than 10 were retained (12,332 genes in total). Pairwise differential expression analysis of each zone against all other zones was calculated using Scanpy rank_genes_groups with a two-sided Wilcoxen rank-sum test (absolute LFC > 1.5; adjusted p-value < 1E-8 by Benjamini-Hochberg correction). The data file contains the significantly zonated genes (one sheet per zone). Scores = scanpy-reported test score. LFC = Log 2 fold change for the indicated zone versus all other zones. Pvals = raw P value. Pvals_adj = Benjamini-Hochberg-adjusted P value.
