# Supplementary material for: SMURF: soft-segmentation for single-cell reconstruction and topological analysis of spatial transcriptomic data
Source: Nat Commun. 2026 Jun 26;17:7990. doi: 10.1038/s41467-026-74464-4 (PMC13447825; doi:10.1038/s41467-026-74464-4)
Supplement: Supplementary file 3 [file 41467_2026_74464_MOESM4_ESM.pdf]

nature portfolio

Corresponding author(s): Joseph D. Dougherty, Robi D. Mitra, Brian D. Muegge

Last updated by author(s): 5/14/2026

Reporting Summary

Nature Portfolio wishes to improve the reproducibility of the work that we publish. This form provides structure for consistency and transparency in reporting. For further information on Nature Portfolio policies, see our Editorial Policies and the Editorial Policy Checklist.

Statistics

For all statistical analyses, confirm that the following items are present in the figure legend, table legend, main text, or Methods section.

n/a	Confirmed	
☐	☒	The exact sample size (*n*) for each experimental group/condition, given as a discrete number and unit of measurement
☐	☒	A statement on whether measurements were taken from distinct samples or whether the same sample was measured repeatedly
☐	☒	The statistical test(s) used AND whether they are one- or two-sided<br>*Only common tests should be described solely by name; describe more complex techniques in the Methods section.*
☐	☒	A description of all covariates tested
☐	☒	A description of any assumptions or corrections, such as tests of normality and adjustment for multiple comparisons
☐	☒	A full description of the statistical parameters including central tendency (e.g. means) or other basic estimates (e.g. regression coefficient) AND variation (e.g. standard deviation) or associated estimates of uncertainty (e.g. confidence intervals)
☐	☒	For null hypothesis testing, the test statistic (e.g. *F*, *t*, *r*) with confidence intervals, effect sizes, degrees of freedom and *P* value noted<br>*Give P values as exact values whenever suitable.*
☒	☐	For Bayesian analysis, information on the choice of priors and Markov chain Monte Carlo settings
☒	☐	For hierarchical and complex designs, identification of the appropriate level for tests and full reporting of outcomes
☐	☒	Estimates of effect sizes (e.g. Cohen's *d*, Pearson's *r*), indicating how they were calculated

Our web collection on statistics for biologists contains articles on many of the points above.

Software and code

Policy information about availability of computer code

Data collection	No new data were collected in this study. All analyzed data were obtained from previously published or publicly available datasets.
Data analysis	SMURF is implemented as an open-source Python package available at https://github.com/The-Mitra-Lab/SMURF; scripts to reproduce all figures are available at https://github.com/The-Mitra-Lab/SMURF_figures and archived on Zenodo (https://doi.org/10.5281/zenodo.20077907). Spatial and single-cell analyses were performed in Python using Scanpy v1.10.0 and AnnData, including PCA, neighborhood graph construction, UMAP, Leiden clustering, diffusion pseudotime, and PAGA. Nuclei segmentation and spatial image processing used Squidpy v1.4.1 together with StarDist v0.8.5. Optimization and deep-learning components of SMURF (count deconvolution) were implemented in PyTorch v2.2.1. Zonation and pathway analyses used gprofiler-official v1.0.0 for KEGG enrichment and XGBoost v2.1.1 for classification of crypt versus villus cells. Transcription-factor regulatory networks were inferred with pySCENIC v0.12.1+6.g31d51a1. For benchmarking, we additionally used 10x Genomics Space Ranger v4.0.1, the Visium HD 8-µm binning workflow, the Bin2cell v0.2.0 package, and Spateo v1.1.1 morphological expansion, as detailed in the Methods.

For manuscripts utilizing custom algorithms or software that are central to the research but not yet described in published literature, software must be made available to editors and reviewers. We strongly encourage code deposition in a community repository (e.g. GitHub). See the Nature Portfolio guidelines for submitting code & software for further information.

Data

Policy information about availability of data

All manuscripts must include a data availability statement. This statement should provide the following information, where applicable:

- Accession codes, unique identifiers, or web links for publicly available datasets
- A description of any restrictions on data availability
- For clinical datasets or third party data, please ensure that the statement adheres to our policy

We analyzed multiple public spatial transcriptomics datasets across sequencing-based and imaging-based platforms, together with one single-cell RNA-seq dataset, one in situ hybridization dataset, and one published spatial proteomics dataset used for exploratory unrolling. Public datasets were obtained from 10x Genomics (Visium HD mouse brain, mouse small intestine, mouse embryo, and human tonsil; Xenium mouse brain replicates and human skin melanoma), NanoString (human frontal cortex CosMx), Zenodo/Choi et al. 2023 (mouse retina MERFISH, sample VZG105a WT2), STOmics MOSTA/Chen et al. 2022 (mouse olfactory bulb Stereo-seq, dataset "Mouse olfa S2.h5ad"), published Seq-Scope mouse liver data, GEO GSE130113 (Seiler et al. small-bowel resection scRNA-seq), the Allen Brain Atlas (Slc1a2 ISH), and published mouse intestine CODEX data used for exploratory unrolling. All datasets analyzed in this study were previously published or publicly available; no new data were generated. Details are provided in the Data Availability statement.

Research involving human participants, their data, or biological material

Policy information about studies with human participants or human data. See also policy information about sex, gender (identity/presentation), and sexual orientation and race, ethnicity and racism.

Reporting on sex and gender	Sex and gender information was not consistently available for the publicly available human datasets analyzed in this study and was not incorporated into the analyses.
Reporting on race, ethnicity, or other socially relevant groupings	Race, ethnicity, and other socially relevant groupings were not available for the public datasets analyzed here and were not used in this study.
Population characteristics	Participant-level metadata were limited to those provided in the original public datasets and source publications.
Recruitment	No new participants were recruited for this study; all human data were obtained from previously published or publicly available datasets.
Ethics oversight	Ethics oversight for collection of the original human tissue samples is described in the corresponding source publications. No new human subjects research was conducted in this study.

Note that full information on the approval of the study protocol must also be provided in the manuscript.

Field-specific reporting

Please select the one below that is the best fit for your research. If you are not sure, read the appropriate sections before making your selection.

☒ Life sciences ☐ Behavioural & social sciences ☐ Ecological, evolutionary & environmental sciences

For a reference copy of the document with all sections, see nature.com/documents/nr-reporting-summary-flat.pdf

Life sciences study design

All studies must disclose on these points even when the disclosure is negative.

Sample size	No prospective sample-size calculation was performed; exact n values are reported in the figure legends and Statistics and Reproducibility section.
Data exclusions	Quality-control filtering was applied as described in the Methods. For the mouse small intestine Visium HD analysis, 28,383 of 404,382 reconstructed cells (7.0%) were excluded based on pre-established quality-control criteria. For zonation analyses, only complete villi were retained, and a small number of artifact-containing regions were excluded after manual review.
Replication	Analyses were performed across multiple independent previously published datasets spanning sequencing-based and imaging-based platforms (Visium HD, Stereo-seq, Seq-Scope, CosMx, MERFISH and Xenium Prime) and yielded consistent qualitative results. All code and analysis scripts are publicly available to enable independent replication.
Randomization	No prospective randomization was performed because this study reanalyzed previously generated public datasets and did not assign subjects or samples to experimental groups. Grouping was based on observed spatial position, dataset, or annotation.
Blinding	Blinding was not relevant because analyses were computational reanalyses of public datasets; any manual exclusions were based on obvious image artifacts.

Reporting for specific materials, systems and methods

We require information from authors about some types of materials, experimental systems and methods used in many studies. Here, indicate whether each material, system or method listed is relevant to your study. If you are not sure if a list item applies to your research, read the appropriate section before selecting a response.

Materials & experimental systems

n/a	Involved in the study
☒	☐ Antibodies
☒	☐ Eukaryotic cell lines
☒	☐ Palaeontology and archaeology
☒	☐ Animals and other organisms
☒	☐ Clinical data
☒	☐ Dual use research of concern
☒	☐ Plants

Methods

n/a	Involved in the study
☒	☐ ChIP-seq
☒	☐ Flow cytometry
☒	☐ MRI-based neuroimaging

Plants

Seed stocks

Report on the source of all seed stocks or other plant material used. If applicable, state the seed stock centre and catalogue number. If plant specimens were collected from the field, describe the collection location, date and sampling procedures.

Novel plant genotypes

Describe the methods by which all novel plant genotypes were produced. This includes those generated by transgenic approaches, gene editing, chemical/radiation-based mutagenesis and hybridization. For transgenic lines, describe the transformation method, the number of independent lines analyzed and the generation upon which experiments were performed. For gene-edited lines, describe the editor used, the endogenous sequence targeted for editing, the targeting guide RNA sequence (if applicable) and how the editor was applied.

Authentication

Describe any authentication procedures for each seed stock used or novel genotype generated. Describe any experiments used to assess the effect of a mutation and, where applicable, how potential secondary effects (e.g. second site T-DNA insertions, mosiacism, off-target gene editing) were examined.
