# Supplementary material for: SMURF: soft-segmentation for single-cell reconstruction and topological analysis of spatial transcriptomic data
Source: Nat Commun. 2026 Jun 26;17:7990. doi: 10.1038/s41467-026-74464-4 (PMC13447825; doi:10.1038/s41467-026-74464-4)
Supplement: Supplementary file 4 [file 41467_2026_74464_MOESM5_ESM.pdf]

nature portfolio

Peer Review File

SMURF: soft-segmentation for single-cell reconstruction and topological analysis of spatial transcriptomic data

Corresponding Author: Dr Brian Muegge

This file contains all reviewer reports in order by version, followed by all author rebuttals in order by version.

Version 0:

Reviewer comments:

Reviewer #1

(Remarks to the Author)
The manuscript by Guo et al describes a new method for analysis of tissue based spatial transcriptomic data named SMURF (soft-segmentation for single-cell reconstruction and topological analysis of spatial transcriptomic data). The authors importantly address a couple of key issues in the current repertoire of spatial analysis methods – the issue of resolution and assignment of transcripts and analysis that incorporates underlying structural tissue architecture. The manuscript ultimately provides a series useful methods and tools and provides an important analytical advance for the field. Some of the biological insights in the intestine largely re-capitulate previous frameworks and some of the interpretation falls into the trap of overreliance on previous (and sometimes lower resolution) descriptions and data around intestinal zonation. However, the spatial /zonated transcriptional network analysis is both novel and likely to be a valuable resource for future studies.

Some specific points are outlined below

1. Single cell reconstruction
a. The authors compare their technique with three techniques, Bin2Cell, the 8um method which is a default method in Visium and nuclear segmentation alone (and Spateo in part). A concern is that the 8um and nuclear segmentation alone methods are well known to have very significant limitations and there is other more recent work that is not benchmarked. There are several methods such as Sparcle that enable single cell transcript assignment, with others such as SCS, (despite the inherent limitation in the underlying assumption of single cell assignment of capture elements), that also enable subcellular resolution of transcripts. It would seem premature to conclude that “SMURF improves single cell reconstruction of high-resolution ST data” without comparing /benchmarking against these methods in addition to Bin2Cell.
b. The main methodological advance here is that the transcripts in between two neighboring cells are deconvoluted between the cells based on cosine similarity. Given that neighboring cells are likely to be similar, will trying to implement deconvolution actually improve performance against a spatial distance weighted random distribution of transcripts?
c. Most enterocytes in villus of the small intestine have a distinct cellular morphology which is columnar/rectangular rather than circular, while most immune cells can be thought of as circles. Given the concentric nature of the soft-segmentation approach, is there be an inherent bias or difference in accuracy in the reconstruction of the transcripts between the different cell types? Is there undercounting of transcripts, because the actual tissue boundaries are not defined -especially in the intestine? For example, in Figure 2b, there are clearly regions where transcripts are recorded in Bin2cell but potentially neglected in SMURF.
d. What are the upper limits on the cell expansion steps in terms of nuclear distance? This should explicitly stated.

2. Implementation of “the unrolling analysis” that identifies complex morphological structures based on spatial clustering. This novel approach towards understanding underlying tissue architecture is an important advance in the field. I appreciate the overall implementation of the technique, and I think it is analytically sound, at least at the broad zonation of the tissue. However, a limitation that should be discussed is that there is no benchmarking (sensitivity/specificity) of how well the method resolves tissue structures (especially villus, crypts, etc.).
a. The methods state that “We then selected the clusters corresponding to the muscularis externa and myenteric plexus to define the x-axis” How were these defined?
b. Line 820: “To detect the crypt–villus boundary, we first performed a rough division based on a normalized distance threshold of 9” It is not very clear how this normalization is conducted. Is this constant through the small intestinal tissue

even though the length and shape of villi and ratio with crypt depth changes through the intestine ?
c. Line 847: “We selected only intact complete villi, including tip enterocytes, for zonation analysis.” Is this done manually?
d. It is not clear what input data is used to assign cells to the crypt to villus axis. Is it the entire tissue or individual villi?

3. The major characterization in the intestine is that of transcript zonation along the villus axis with implication for function. There are couple of issues that should be discussed to qualify the interpretation. Firstly, although largely spatially arranged, maturation/differentiation and lineage trajectory is not exclusively positional – this is particularly the case for Paneth cells which are not part of the enterocyte maturation path, as well as several of the rarer secretory cells analyzed which may have discrete lineage branch points. Secondly more broadly, transcript (especially for transporters and channels) is often not correlated with functionally localized protein – this is less of an issue at the extremes (villus tip and crypt base) but in the intermediate regions.
a. The statistics and experimental details in Figures 3-5 need to be more clearly described. Exactly what was analyzed was often unclear. E.g. the number of tissue structures analyzed for each experiment. The error bounds for the expression levels in Figure 5d as an example (and others) are missing.

b. What are the limitations of the small number of UMI per cell. One of the big issues with spatial transcriptomics, as the authors point out in the small intestine, is that median number of UMI per cell is 165. The authors should comment on limitations on any analysis including theirs. Supplementary 1 which starts to look at this analytically is helpful (mentioned below) and further insights /discussion would be useful as the authors have clearly thought about this issue. This is especially important for any biological insights.
c. Given the low UMI per cell, I am curious to know about the limitations associated with cell annotations? How many cells were not annotated because of the lack of data. Also, what was the overall cellular composition of their tissues and how does this compare to scRNA-seq atlas data.

4. Misc Findings: The authors should be commended as there are several important technical findings that are often taken for granted but have been explicitly tested here.
• Using Negative Binomial to fit spatial data and comparison with various other distributions.
a. I think this is an important section and I would like to see this highlighted more in the main text.
• Normalization artifact in the 8 μm method: I think is important especially given that the default binning is 8x8 in10X Visium and has been used in number of papers. I think the artifacts if detailed better will be useful to the community.

5. Minor points
a. Figure 2, spelling of Brain in 2c.
b. Figure legends in supplementary material should be expanded.
c. Are the figures S5 and S6 repeated?
d. How is the performance of the lite version to the GPU version? Where should one use the GPU version? Could the authors expand on the actual runtime of the two versions. I was unable to directly test the software.

(Remarks on code availability)

Reviewer #2

(Remarks to the Author)
The work for the Muegge team presents a new analytical tool to process spatial transcriptomics data and deconvolute tissues to provide a more consistent structure for their analysis and interpretation. This tool, called SMURF, couples existing nuclear segmentation approaches (imaged base) with an algorithm to then assign spatial transcriptomic capture spots to nearby nuclei based upon their distance and expression patterns. The authors show notable improvements over a popular Bin2cell algorithm or the default 8um bin analysis in figure 2. In figures 3-4 they show how their application can “unwind” tissues to provide more order in the analysis, such as flattening the curvature of the brain cortex or unwinding a swiss roll of the intestine. Finally, in figures 5-6, the authors investigate how they can analyze spatial features in the intestine, focusing on the crypt-villus axis of the epithelium. They use SCENIC to predict important transcription factors along each “zone” of the intestinal differentiation trajectory. They use published scRNAseq data to show that the upper villus zones are the most plastic zones after a perturbation such as a small bowel resection. The work is timely in that there is a need for improved spatial transcriptomics analysis. The work is nicely written and presented. The current manuscript has a few significant shortcomings that I think should be addressed to more clearly demonstrate that it is a tool worth broadly deploying throughout the informatics community:

A key aspect of benchmarking SMURF for readers to make a decision on whether to implement it into their analysis will be to show Bin2cell results for the analysis in figure 2d-e (it seems only nuclear segmentation data is shown) and, more importantly, compare SMURF to the latest release of the LOUPE browser (v9.0 or later) that deployed this June, as the approach SMURF and 10x takes seems similar.

Additionally, how long does it take to run SMURF vs LOUPE vs Bin2Cell? What are the processing and data storage requirements?

The authors mention broad applicability of their method to deconvolute other tissues and data types (such as spatial metabolomics or proteomics). What about other imaging data? Could the authors demonstrate this process for immunostaining, for example, to further expand the utility of their work?

Minor:
The utility of SMURF to make new discoveries is fairly limited within the current paper, though an attempt to do so is provided in figure 6. This seems reasonable given the paper's focus on the analysis tool. The villus tip repatterning after resection is fairly modest, with only 34 genes differentially expressed in their re-analysis of published scRNAseq data. The authors do mention important caveats to their findings though, so this concern is modest.

(Remarks on code availability)

Reviewer #3

(Remarks to the Author)
The manuscript introduces SMURF, a timely and well-motivated framework for assigning spot-level spatial transcriptomics data to individual cells by integrating nuclei segmentation, histological information, and iterative soft assignment. The method demonstrates improved transcript recovery and clustering precision and a strength that reveals transcriptional organization not readily apparent in two-dimensional analyses using a manifold-based strategy for unrolling complex tissue geometries. The application to intestinal zonation is biologically convincing, and the software appears well packaged with clear documentation.

However, the robustness of SMURF seems to depend strongly on the accuracy of nuclei segmentation, while quantitative evaluation of segmentation specificity remains limited. In addition, broader and more systematic benchmarking against state-of-the-art methods would be necessary to firmly establish SMURF as a reliable standard for single-cell–resolution spatial transcriptomics. Strengthening the methodological validation would substantially enhance the impact and generalizability of the framework.

Major comments:
1. The manuscript convincingly shows that SMURF recovers more UMIs per cell than nuclei-based approaches. However, increased sensitivity may come at the expense of specificity. Could the authors quantify potential transcript "bleed-over" or contamination? A negative control analysis assessing how frequently cell-type specific markers are incorrectly assigned to neighboring cells—relative to rigid nuclei segmentation—would help evaluate the trade-off introduced by the soft segmentation strategy.

2. The current benchmarking focuses on comparisons with rigid binning and nuclei-only segmentation. To better position SMURF among state-of-the-art methods, it would be valuable to include comparisons with existing probabilistic molecule assignment approaches adapted for high-resolution data (e.g., Baysor, JSTA, or Cellpose). Additionally, for the deconvolution step (Figure 1), comparison with established deconvolution methods such as RCTD or Cell2Location (applied to small bins) would help justify the proposed deep learning formulation. Demonstrating performance relative to state-of-the-art methods in common downstream tasks (e.g., spatially variable gene detection, spatial domain identification) would further strengthen the appeal of SMURF to potential users.

3. The method introduces a deep learning module to resolve spots shared across multiple cells. Given the additional computational complexity, it would be helpful to clarify the specific contribution of this component. An ablation analysis quantifying the performance gain attributable to this module, relative to the soft-segmentation strategy alone, would help justify its inclusion in the pipeline.

There are also minor comments.

1. The methods state that the analysis focuses on regions covered by spots, which may include cells only minimally overlapped by a spot. Could the authors clarify whether the method can handle cells that are entirely uncovered by any spots? If so, highlighting this capability would strengthen the manuscript.

2. A minimum overlap fraction of 1/6 is used when assigning shared spots to cells. Please clarify how this threshold was chosen and whether the results are sensitive to this parameter.

3. Leiden clustering is used to generate initial clusters, but the resolution parameter can strongly influence the number of clusters. Could the authors specify the resolution value used in SMURF and explain whether it is fixed or dataset-dependent? Additional guidance on how this parameter is selected to optimize results would improve reproducibility.

(Remarks on code availability)
SMURF can be easily installed in a Python ecosystem and a clean and comprehensive documentation and tutorial is also available on the accompanying website.

Reviewer #4

(Remarks to the Author)
This paper presents the package SMURF which soft assigns Visium HD spots to cells and maps layered biological structures such as Swiss rolled intestines and the cerebral cortex onto a straight rectilinear grid. The authors reported improved cell type identification thanks to improved soft assignment of spots to cells and deconvolution of spots shared by multiple cells. The authors also present an in depth analysis of unrolled mouse intestine and interesting biological findings on zonation of the villi and differences between the proximal and distal intestine. SMURF seems to be a much needed preprocessing tool for Visium HD and the unrolling can be applied to other methods as well. Below are some mostly minor questions and comments on this paper:

1. There have been other methods that unrolls Swiss rolled intestines in spatial transcriptomics, for example: https://ludvigla.github.io/semla/articles/digital_unrolling.html and https://doi.org/10.1093/bioinformatics/btad140. It would be helpful to discuss how SMURF's unrolling relates to the existing unrolling methods, much like the discussions on bin2cell when it comes to assigning spots to cells.

2. Regarding zonation of the villi, the villi were divided into 6 zones. Gene expression from the zones was pseudo-bulked and the Wilcoxon rank sum test was used to identify genes differentially expressed in each zone. What's the reason to use 6 discrete zones instead of the continuous values of the y-axis with a spatially variable gene method that accounts for spatial autocorrelation in this axis? And why 6 zones instead of a different number?

3. Bin2cell normalizes Visium HD data row by row due to the stripy artifact at the 2 micron resolution. Do you find such normalization relevant or helpful to downstream analyses? What may be other Visium HD technical artifacts?

(Remarks on code availability)
The package is available from pip and has a nice and detailed documentation website with instructions on installing and using the package.

Version 1:

Reviewer comments:

Reviewer #1

(Remarks to the Author)
The authors have very comprehensively addressed all my previous questions including the key benchmarking comparisons requested. I have no further comments or suggestions. The work represents an important and useful addition to spatial tissue analysis methods.

(Remarks on code availability)

Reviewer #2

(Remarks to the Author)
My concerns were all addressed.

(Remarks on code availability)

Reviewer #3

(Remarks to the Author)
Most of my comments have been addressed in this revision, but one comment remains unresolved.

“SMURF produced spatial domains that more closely matched known hippocampal anatomy than the other methods (Supplementary Fig.S3).” This is a comparison of SMURF with other methods for spatial domain detection. While the visual results suggest that SMURF outperforms other approaches, the argument would be significantly strengthened by including a quantitative measure of how closely the inferred domains match the reference anatomy.

In spatial transcriptomics methods that perform clustering at spot resolution, such “closeness” is typically evaluated using metrics like the adjusted Rand index (ARI). Although the qualitative comparison is compelling, reporting these scores for each method would make the results more rigorous and convincing. Moreover, providing quantitative benchmarks would be useful for future studies on single-cell transcript assignment, as it would help identify areas for further improvement.

SMURF addresses a different problem compared to methods such as GraphST or Spatial-MGCN, where clustering is the primary objective. Consequently, benchmark datasets used for GraphST and Spatial-MGCN typically include clustering-

specific ground truth annotations that can serve as references for evaluation using metrics such as the adjusted Rand index (ARI). In contrast, numerical evaluation of SMURF may be more challenging due to the lack of clustering-specific ground truth annotations in datasets relevant to its task. If quantitative evaluation is not feasible, the authors should clearly explain why. Otherwise, the inclusion of appropriate quantitative metrics is strongly encouraged.

(Remarks on code availability)

Reviewer #4

(Remarks to the Author)
The other reviewers have given very insightful and helpful comments, and the authors have thoroughly and thoughtfully responded to these comments. Specifically, the benchmarks comparing SMURF to other relevant methods for assigning bins to cells and for unrolling the intestine Swiss roll convincingly show that SMURF is a valuable contribution in this area. However, the t-test comparing SMURF with deep learning and SMURF-lite does not seem very meaningful, because the effect size is so small despite the very significant p-values.

(Remarks on code availability)

Version 2:

Reviewer comments:

Reviewer #3

(Remarks to the Author)
My concerns were all addressed in this revision. I have no further comments or suggestions.

(Remarks on code availability)

Open Access This Peer Review File is licensed under a Creative Commons Attribution 4.0 International License, which permits use, sharing, adaptation, distribution and reproduction in any medium or format, as long as you give appropriate credit to the original author(s) and the source, provide a link to the Creative Commons license, and indicate if changes were made.
In cases where reviewers are anonymous, credit should be given to 'Anonymous Referee' and the source.
The images or other third party material in this Peer Review File are included in the article's Creative Commons license, unless indicated otherwise in a credit line to the material. If material is not included in the article's Creative Commons license and your intended use is not permitted by statutory regulation or exceeds the permitted use, you will need to obtain permission directly from the copyright holder.
To view a copy of this license, visit https://creativecommons.org/licenses/by/4.0/

RESPONSE TO REVIEWERS

We thank the four anonymous reviewers for their helpful and constructive feedback. In response, we have performed extensive additional analyses and computational experiments to address each of their comments and have revised the manuscript accordingly. As a result, the manuscript is now more rigorous and polished. We have also updated our Git repository and tutorial to ensure that readers can reproduce the revised analyses.

We were encouraged that the reviewers responded positively to SMURF and the biological findings presented in the manuscript. For example, Reviewer 1 felt "The authors importantly address a couple of key issues in the current repertoire of spatial analysis methods – the issue of resolution and assignment of transcripts and analysis that incorporates underlying structural tissue architecture" and that SMURF "provides an important analytical advance for the field." Reviewer 2 found that SMURF provides 'notable improvements' over existing methods and found the work "timely" and "nicely written and presented." Reviewer 3 thought SMURF was "a timely and well-motivated framework" and found our use cases "biologically convincing." Reviewer 4 stated "SMURF seems to be a much needed preprocessing tool for Visium HD".

The reviewers also raised several important concerns. All four reviewers touched upon one common theme: the need to benchmark SMURF against additional methods, particularly Space Ranger (Loupe Browser), which is now the default analysis software provided by 10x Genomics for Visium HD.

Below, we first detail our efforts to address the request for additional benchmarking, and then we describe the analyses we performed and revisions we made to address each individual reviewer's comments.

Major Theme - Benchmark SMURF segmentation against additional methods

Our original manuscript benchmarked SMURF's segmentation and transcript assignment algorithm against three competing methods: Bin2cell [1], the 8μm method, and nuclear segmentation. Three of the four reviewers suggested we benchmark SMURF against additional methods, especially Space Ranger (Loupe Browser) [2,3], which has recently become the software 10x Genomics recommends for Visium HD analysis, but also other methods like SCS [4], Baysor [5], Sparcle [6], and JSTA [7]. Importantly, the reviewers also suggested that we also benchmark the computational time required to run each analysis.

Specifically, **<u>Reviewer 1</u>** wrote "*there is other more recent work that is not benchmarked. There are several methods such as **Sparcle** that enable single cell transcript assignment, with others such as **SCS**, (despite the inherent limitation in the underlying assumption of single cell assignment of capture elements), that also enable subcellular resolution of transcripts. It would seem premature to conclude that "SMURF improves single cell reconstruction of high-resolution ST data" without comparing benchmarking against these methods in addition to Bin2cell.*" **<u>Reviewer 2</u>** wrote: "*A key aspect of benchmarking SMURF for readers to make a decision on*

*whether to implement it into their analysis will be to show Bin2cell results for the analysis in figure 2d-e (it seems only nuclear segmentation data is shown) and, more importantly, compare SMURF to the latest release of the **LOUPE** browser (v9.0 or later) that deployed this June, as the approach SMURF and 10x takes seems similar… Additionally, how long does it take to run SMURF vs LOUPE vs Bin2cell? What are the processing and data storage requirements?"* **Reviewer 3** wrote, "*To better position SMURF among state-of-the-art methods, it would be valuable to include comparisons with existing probabilistic molecule assignment approaches adapted for high-resolution data (e.g., **Baysor, JSTA, or Cellpose**).*"

We agree it is important to evaluate SMURF against these additional methods. We therefore made extensive efforts to benchmark all of the suggested methods using the mouse brain dataset. Since many of the suggested methods were not designed for the analysis of Visium HD data, we adapted and/or refactored the source code to accept Visium HD data formats. Our benchmarking efforts are summarized in the Supplementary Note 1 (p.1–5), which include a new Supplementary Table reproduced below, which inludes information about: (i) whether the method can natively accept Visium HD inputs, (ii) whether modifications to the codebase were required, (iii) runtime and computational resource usage when it ran successfully, and (iv) if we were able to benchmark the method, or if it failed due to compute time constraints. In addition to this summary table, the actual results of our additional benchmarking are reported in Supplementary Figure S2, which we have pasted below for the reviewers' convenience.

Table S1: Summary of Extended Benchmarking.

Method	Works out of the box	Required codebase modifications	Runtime and computational resource usage (mouse brain dataset)	Successfully benchmarked
SMURF	Yes	None	2.92 h for SMURF and 1.69 h for SMURF-lite (3-run averages)	Yes
Space Ranger (Loupe Browser)	Yes	None	N/A (runs automatically with FASTQ alignment)	Yes (only works for H&E staining)
Bin2cell	Yes	None	5.62 min (3-run average)	Yes
8 μm binning	Yes	None	N/A (runs automatically with FASTQ alignment)	Yes
Baysor	No	None	26.48 h (single run; summed across 3 × 3 slides)	Yes
SCS	No	Yes	100.51 h (4.12 days; single run summed across 4 × 5 slides, with GPU; substantially longer without GPU)	Yes
Sparcle	No	Yes	Estimated to be hundreds of years	Failed due to excessive compute requirements
JSTA	No	Yes	Estimated to require months	Failed due to excessive compute requirements

We made the following changes to the manuscript in response to these reviewer comments:

1. **Benchmarking against Space Ranger (Loupe Browser), SCS, Baysor, Nuclei segmentation methods (Stardist/Cellpose).**

We agree with the reviewers that these are crucial comparators. We have added head-to-head benchmarks for all of these methods vs. SMURF in the main or Supplementary Note 1 as explained below.

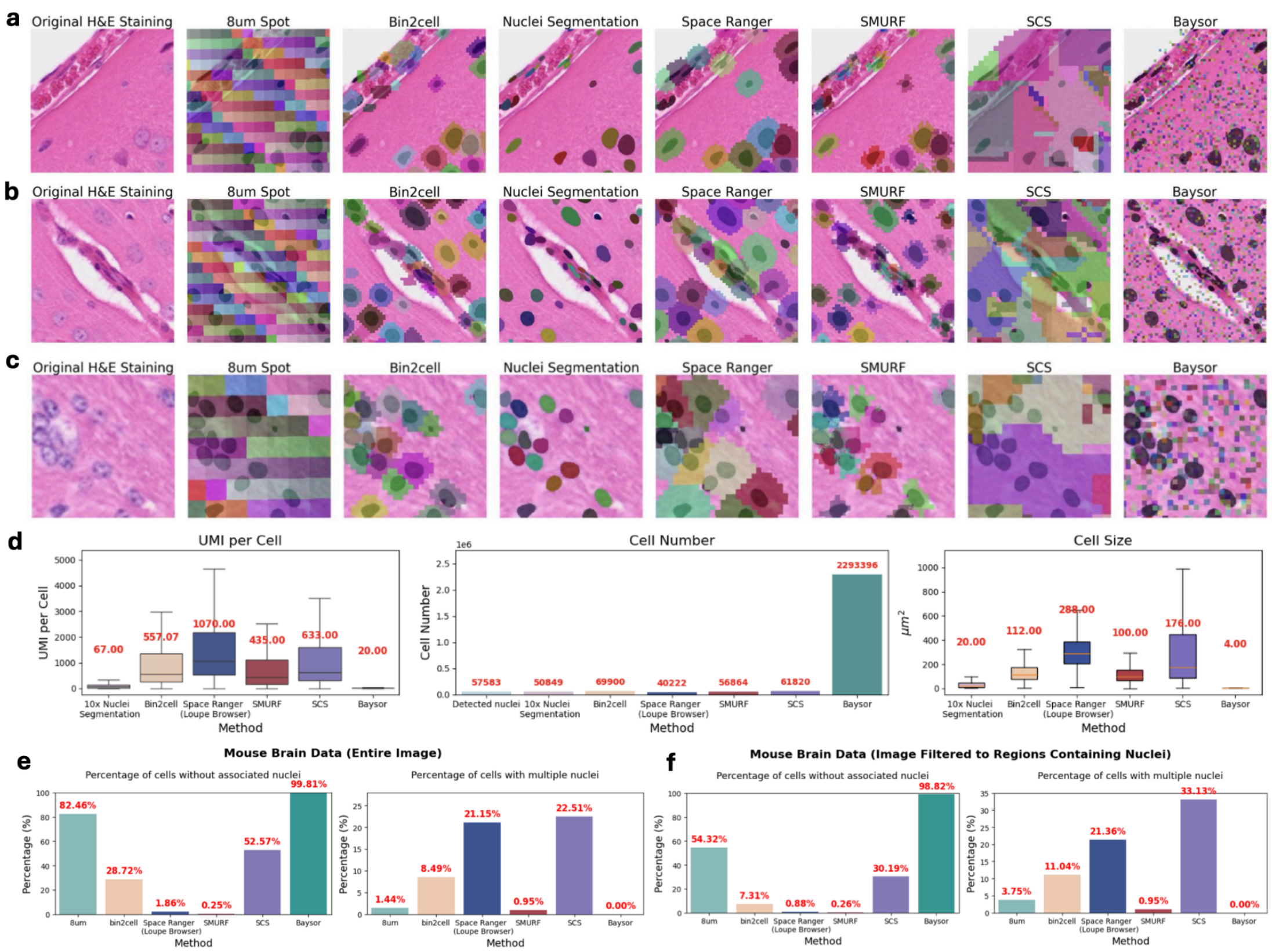

Fig. S2: **Extended comparison of ST segmentation methods on the mouse brain dataset.** (a-c) Representative images illustrating how different ST segmentation methods aggregate Visium HD capture spots to define cells in mouse brain tissue. Columns show the original H&E staining, 8 μm spots, and cell assignments from Bin2cell, nuclei segmentation, Space Ranger, SMURF, SCS, and Baysor. Capture spots are shaded according to their assigned cell. (d) Number of UMIs assigned per cell, total cells detected, and cell size for each method. (e,f) Percentage of cells without an associated nucleus and percentage of cells containing multiple nuclei, calculated using the entire image (e) or only regions containing nuclei (f). Abbreviations: ST, spatial transcriptomics; UMI, Unique Molecular Identifier.

A. Space Ranger (Loupe Browser): Loupe is the 10x Genomics visualization platform for exploring Visium HD outputs, while Space Ranger performs the primary analysis. For Visium HD datasets with H&E images, Space Ranger can generate both image

alignment and nucleus/cell segmentation results. We agree that Space Ranger (Loupe Browser) is a crucial comparator because it is now the default Visium HD software for data with H&E images. Therefore, we have added to Figure 2 in the main text, a head-to-head benchmark of SMURF versus Space Ranger 4.0.1 (Loupe Browser) alongside the previously reported benchmarks vs Bin2cell, the 8 μm method, and nuclear segmentation (new Figure 2 panels a-d; associated text in p.4–5, l.155–194). We found that Space Ranger does not accurately segment cells relative to SMURF. For example, 21.15% of the cells detected by Space Ranger contain multiple nuclei, compared to only 0.95% for SMURF (see Supplementary Figure S2 from the revised manuscript, which is copied into this document for ease of reference). Space Ranger detects more UMIs per cell, but this clearly comes at the cost of merging a substantial number of neighboring cells.

B. Baysor: We successfully restructured the Visium HD data to make it compatible with the Baysor package. When we benchmarked Baysor on the mouse brain, Baysor detected 2,293,396 “cells”, far exceeding the true number of nuclei (57,583). Perhaps unsurprisingly, 98.81% of the detected cells did not have an associated nucleus, and the average area of the detected cells was only 4.0 μm^2. We believe this is because Baysor was designed for imaging-based spatial transcriptomics which assumes that transcripts are resolved to single-molecule resolution. Baysor has not been deployed to analyze Visium HD before. We discuss this more fully in the short supplemental report at the end of this document that is also included in our Supplementary Note 2 (included in the bundled Supplementary File).

C. SCS: We had to extensively refactor the source code for SCS to run on Visium HD data as explained in Supplementary Note 2. Even with our optimization and refactoring, SCS took 100.5 hours to process the mouse brain dataset on our workstation with GPUs (compared to average 2.92 hours for SMURF). Despite the onerous runtime, we still benchmarked SCS on our Visium HD mouse data set. As shown in Supplementary Figure S2 (copied above), the segmentation with SCS did not resolve single cells as well as SMURF. 52.57% of cells detected by SCS had no associated nucleus and 22.51% of detected cells had multiple nuclei (compared to 0.25% and 0.95% with SMURF) in the entire image.

D. Cellpose and Stardist. These are highly useful tools for nuclei segmentation. In fact, Stardist is the package that SMURF (and Bin2cell and Space Ranger) uses for nuclei segmentation of H&E images. Cellpose is the package SMURF uses for DAPI segmentation. As a result, these methods were already benchmarked in our original manuscript. Stardist segmentation is represented in Figure 2 (labeled as nuclei segmentation). Cellpose nuclei segmentation was used in the DAPI stained human tonsil data that was analyzed in our original submission, now labeled as Supplementary Figure S5 in the revised submission (again labeled as nuclei segmentation). Our benchmarking against these additional methods shows that SMURF continues to demonstrate

substantial improvements in segmentation accuracy, transcript recovery per cell, and downstream clustering/annotation consistency.

We have included all of the additional benchmarking metrics that reviewers requested, including UMI per cell, number of detected cells, and cell size for all of the methods analyzed in the new Supplementary Figure S2. We have added the results for Space Ranger (Loupe Browser) in main text Figure 2.

2. **Benchmarking against other methods.** We attempted to benchmark SMURF against the two additional suggested methods (Sparcle [6], and JSTA [7]), and we report outcomes transparently in Supplementary Table S1. Briefly, we attempted to run these methods on Visium HD data. However, they each required substantial refactoring as explained in Supplementary Note 2. Even after substantial effort to rewrite their code, we found the runtime required to analyze a single dataset would take months to years on our compute setup. We estimated the runtime by applying the algorithms on subsamples of our Visium HD data.

3. **Runtime and computational resource requirements**. As requested by reviewer 2, we have added explicit runtime and resource benchmarking for all suggested methods (new Supplementary Table S1). We report wall-clock times, and we provide practical guidance on which SMURF mode is appropriate given typical user compute environments.

4. **Additional edits throughout the manuscript.** As requested by the reviewers, we modified wording throughout the manuscript to ensure the accuracy of our claims. In particular, we softened broad statements where the reviewers correctly noted that not all state-of-the-art methods could be benchmarked natively on Visium HD at present (see for example, p.4, l.155–160).

Comments from Reviewer 1

1. Single cell reconstruction
a. The authors compare their technique with three techniques, Bin2cell, the 8um method which is a default method in Visium and nuclear segmentation alone (and Spateo in part). A concern is that the 8um and nuclear segmentation alone methods are well known to have very significant limitations and there is other more recent work that is not benchmarked. There are several methods such as Sparcle that enable single cell transcript assignment, with others such as SCS (despite the inherent limitation in the underlying assumption of single cell assignment of capture elements), that also enable subcellular resolution of transcripts. It would seem premature to conclude that “SMURF improves single cell reconstruction of high-resolution ST data” without comparing /benchmarking against these methods in addition to Bin2cell.

Thank you for this important suggestion. We performed extensive additional benchmarking of SMURF against other methods, which is described in detail in our response to Major Theme above with Supplementary Note 1 (section title: *Extended benchmarking of additional cell*

segmentation methods, p 1-5) as well as in the short report (Supplementary Note 2) at the end of this document.

b. The main methodological advance here is that the transcripts in between two neighboring cells are deconvoluted between the cells based on cosine similarity. Given that neighboring cells are likely to be similar, will trying to implement deconvolution actually improve performance against a spatial distance weighted random distribution of transcripts?

Great question. In fact, we use a spatial-distance weighted random distribution in our "SMURF-lite" package. We agree that it is important to quantify what the deep learning step in the full SMURF algorithm contributes to overall accuracy. Before we describe the additional analysis we performed, we should first clarify two points:

1. Deep learning is only one component of SMURF's novel transcript assignment algorithm. When spots shared between two cells of different types are deconvolved, a computationally efficient, "canonical" deconvolution algorithm is used. However, when two spots are shared between cells of the same type, SMURF uses a (moderately) computationally expensive deep learning algorithm to partition the transcripts.
2. SMURF supports two operating modes that differ in how these shared spots are handled: a "full" mode (deep learning-based; typically requiring a GPU for practical runtimes) and a "lite" mode (non-deep learning; runnable on a standard machine). SMURF-lite uses a spatial-distance weighted multinomial distribution to assign transcripts. If a user does not have access to a GPU, they should run SMURF-lite.

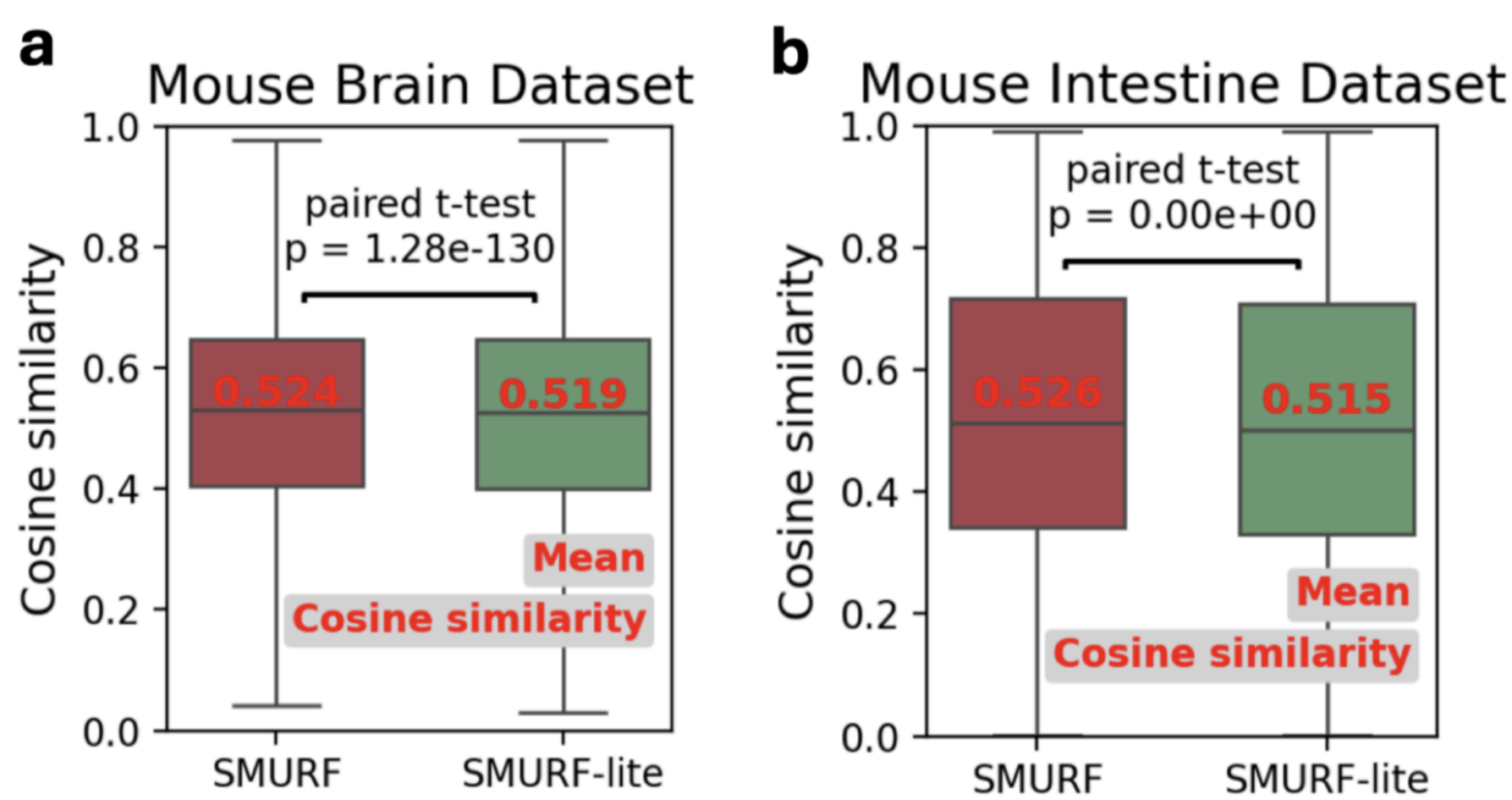

Fig. S6: **Comparison of cosine similarity for SMURF and SMURF-lite.** We compared cosine similarity between reconstructed cells and the mean expression profile of their assigned clusters for SMURF and SMURF-lite. (a) Mouse brain dataset. (b) Mouse small intestine dataset. Red numbers indicate mean cosine similarity. P values were calculated using paired t-tests.

To address the reviewer's request to quantify the improvement in transcript assignment enabled by deep learning, we computed the cosine similarity between the mean profile of each cluster

and each cell within that cluster that received transcript assignments from the deep-learning module in SMURF. We then compared the resulting distribution of cosine similarities with that obtained using the classical deconvolution algorithm implemented in SMURF-lite. A paired t-test showed that cells assigned transcripts by SMURF had significantly higher cosine similarity to their corresponding cell type than did cells assigned transcripts by SMURF-lite (see figure above).

We have revised the Methods to clarify the availability of SMURF-lite (p. 17, l. 740-742), stating that: *“For this step, the "lite" version of SMURF uses a spatially distance-weighted multinomial model to assign counts by random sampling.”* We have also added a more detailed description in the Supplementary Note 1 (section title: *SMURF vs. SMURF-lite: evaluating the contribution of the deep learning module*, p.9, l.172–196).

c. Most enterocytes in villus of the small intestine have a distinct cellular morphology which is columnar/rectangular rather than circular, while most immune cells can be thought of as circles. Given the concentric nature of the soft-segmentation approach, is there an inherent bias or difference in accuracy in the reconstruction of the transcripts between the different cell types? Is there undercounting of transcripts, because the actual tissue boundaries are not defined -especially in the intestine? For example, in Figure 2b, there are clearly regions where transcripts are recorded in Bin2cell but potentially neglected in SMURF.

Thank you for raising this point. We also agree that since Visium HD does not provide direct membrane-boundary information, capturing all the transcripts from complex cellular geometries can be challenging. Any nuclei-guided method (including SMURF, Bin2cell, and Space Ranger) must make implicit assumptions about how far the cytoplasm extends from the nucleus.

For SMURF’s segmentation algorithm, while expansion proceeds in concentric layers around nuclei, the addition of spots is not constrained to expand symmetrically on all “sides” of a nucleus; spots are added based on overlap and assignment criteria and can therefore capture non-symmetric cell shapes when supported by local spatial and expression evidence. SMURF’s default limit for maximum expansion is 2 spots. Cells with highly irregular shapes may extend beyond this default limit, so the user is encouraged to adjust this parameter to determine the best threshold for their data.

We added the following text to our overview of the method in p.3–4, l.142–144 to clarify this point:

“During concentric expansion, new spots can be assigned asymmetrically, allowing SMURF to recover non-circular and elongated cell morphologies when supported by gene expression similarity and proximity.”

We also now discuss potential limitations for large or elongated cells and the trade-offs between sensitivity and specificity when expanding farther from nuclei. We also specify the default maximum expansion distance (two capture spots away from the nucleus by default) and describe how this parameter can be adjusted (p.11, l.524–529).

"SMURF's segmentation algorithm has some caveats. Because Visium HD does not directly provide plasma-membrane boundaries, transcript assignment can be more challenging for large columnar or elongated cells. While SMURF can assign spots to cells asymmetrically, this process depends on a user-defined parameter that specifies the maximum expansion distance from the nucleus which may need to be tuned to maximize the capture of cytoplasmic transcripts."

The reviewer also points out that in the presented images there are places where Bin2cell (and now Space Ranger) associate cytoplasm to nearby cells that SMURF does not. We believe this is because Bin2cell and Space Ranger are overly-tuned for sensitivity at the expense of specificity. For example in that same image, there are clearly examples where Bin2cell and Space Ranger inappropriately group multiple nuclei into the same "cell". We designed the SMURF algorithm to prioritize conservative per-cell assignments when boundary evidence is weak, which can be appropriate for downstream analyses that are sensitive to contamination. Our benchmarking supports this idea; SMURF cells are on average slightly smaller and contain fewer UMIs per cell than Bin2cell and Space Ranger, but with a remarkably lower frequency of multinucleated cells (Figure 2).

1d What are the upper limits on the cell expansion steps in terms of nuclear distance? This should explicitly stated.

We addressed this in our answer to 1c above.

2. Implementation of "the unrolling analysis" that identifies complex morphological structures based on spatial clustering. This novel approach towards understanding underlying tissue architecture is an important advance in the field. I appreciate the overall implementation of the technique, and I think it is analytically sound, at least at the broad zonation of the tissue. However, a limitation that should be discussed is that there is no benchmarking (sensitivity/specificity) of how well the method resolves tissue structures (especially villus, crypts, etc.).

We agree that this is a limitation that should be discussed further, and we have edited the manuscript to discuss this limitation. Specifically, we added the following text in the discussion (p.12, l.535–540):

"One limitation of SMURF's unrolling algorithm is that, while it enabled novel biological results that could be validated using published data in the intestine, it is difficult to rigorously benchmark how well SMURF's unrolling algorithm resolves the fine structure of tissues because there are few gold standard reference datasets for such analysis. Despite this limitation, we show that SMURF's output is highly consistent with biological expectations."

The major challenge with doing comprehensive benchmarking is that there is no gold standard reference data of crypt or villus composition. scRNA-seq atlases can only infer crypt and villus composition because the cells are dissociated from their tissue context. The existing laser capture microdissection study by Moor et al [8] was performed in the jejunum. As we show in

the analysis related to Figure 6, the zonated gene programs in the jejunum and ileum (the intestinal section that we analyzed) are very divergent.

We have added analysis, one new figure, and one new table to our Supplementary Note 1 to support this point. First, we added an image of the intestinal Swiss roll with the crypt-villus boundary line shown along the entire length of the intestine. As discussed in a later response, the boundary value was calculated and refined separately for each crypt-villus unit. Second, we added a table that shows cell type abundance in the crypt and villus epithelial fractions. As expected, cells assigned to the stem/paneth and TA clusters are almost exclusively in the crypt group, which is further validation of our boundary calculations. This analysis was added to the Supplementary Note 1 (Section title: *Crypt–villus boundary analysis in the mouse ileum*, p. 22, l. 398-409)

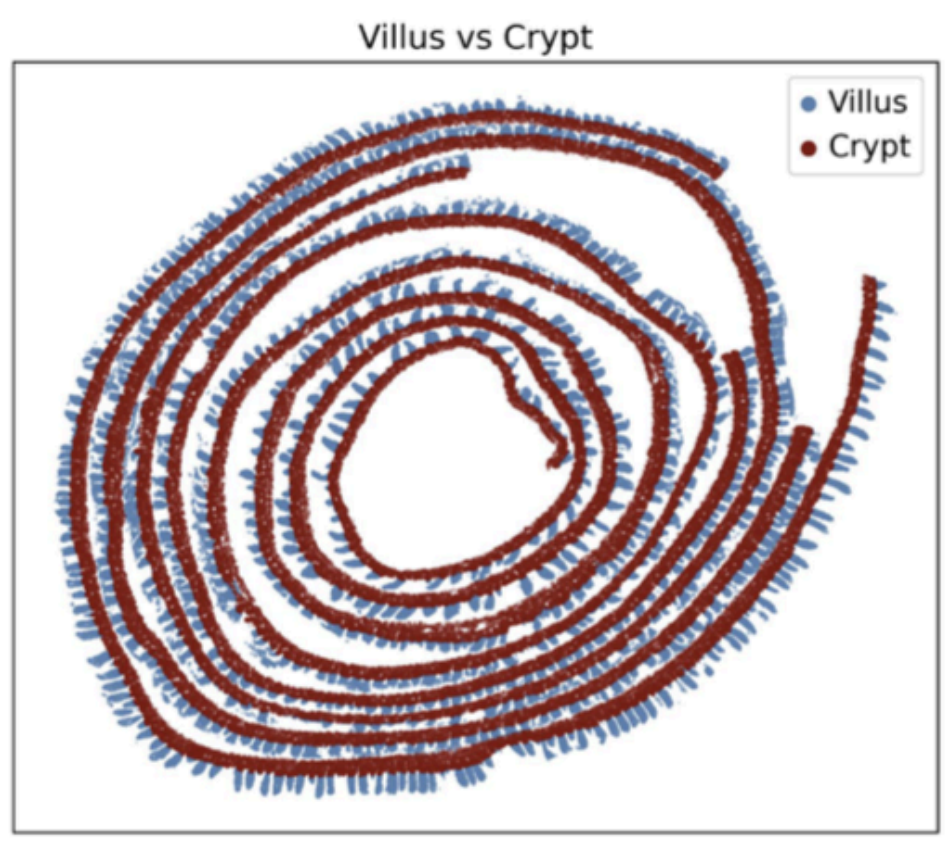

Fig. S16: Validation of the Inferred Crypt–Villus Boundary Across the Entire Length of the Mouse Ileum Swiss Roll.

Table S4: Percentage of Epithelial Cell Types Assigned to the Crypt and Villus Compartments Based on Intact Villi.

Cell type	crypt (%)	villus (%)
Enterocytes	39.62	60.38
Transit Amplifying cells	99.43	0.57
Paneth and stem cells	99.74	0.26

a. The methods state that "We then selected the clusters corresponding to the muscularis externa and myenteric plexus to define the x-axis" How were these defined?

Thank you for raising this point. In the revised Methods (p.20, l.857–863), we now state explicitly how these clusters were identified. Specifically, we added the following text:

"The intestine is surrounded by a muscular layer composed of smooth muscle cells and nerves (myenteric plexus). We used this layer to define the x-axis position representing the longitudinal

axis of the Swiss roll preparation. We identified the clusters representing the muscularis externa and myenteric plexus by their spatial location at the base of the Swiss roll, and confirmed their identity by marker-gene expression (e.g., Acta2, Tagln, and Myh11 for muscularis externa; Snap25, Uchl1, and Prph for myenteric plexus).”

b. Line 820: “To detect the crypt–villus boundary, we first performed a rough division based on a normalized distance threshold of 9” It is not very clear how this normalization is conducted. Is this constant through the small intestinal tissue even though the length and shape of villi and ratio with crypt depth changes through the intestine ?

AND

d. It is not clear what input data is used to assign cells to the crypt to villus axis. Is it the entire tissue or individual villi?

Thank you for the comment. We have edited the Methods to clarify these issues. Briefly, the initial division threshold is a starting point for iterative analysis, and is dynamically adjusted at each villus. We algorithmically identified villi using all of the cells in the dataset, with light manual curation. We added the following text at p.20, l.865–870 and p.21, l.896–900:

“The y-axis was assigned using all cells in the tissue except those used to define the x-axis, rather than being computed separately for individual villi. The corrected y-axis is first obtained in physical units (μm), and then we calculate a normalized corrected y-axis by setting the tip of each villus to a fixed value of 20 (Extended Data Figure 3c; Extended Data Figure 4). This facilitates comparison across villi of different lengths and shapes.”

AND

“To detect the crypt–villus boundary, we initially split each crypt-villus unit into a lower and upper section at normalized y-axis values of 9. Cells with normalized y-axis values less than 9 were assigned to the crypt group, and those with higher values were assigned to the villus group.”

c. Line 847: “We selected only intact complete villi, including tip enterocytes, for zonation analysis.” Is this done manually?

We identified intact villi by algorithmic identification of villus tips to find the locations of complete villi. We applied light manual QC afterwards to verify the output. We edited the methods (p.22, l.928–939) with the following text to clarify this issue.

“We selected only complete villi for zonation analysis. We used the presence of tip enterocytes as a marker of villus completeness. To select intact villi in an unbiased manner, the intestine was divided into small sections along the x-axis. We applied a Kolmogorov–Smirnov test to evaluate the distribution of tip enterocytes in each section against a uniform distribution. To ensure comprehensive coverage, multiple candidate sections from each area that passed the

test were retained. We refined the final list of villi by excluding a small number of sections with obvious artifacts on manual review of the corresponding H&E image. This procedure retained over 100 intact villus structures for downstream zonation analysis. We then calculated an x-axis anchor position for each of the intact discrete villi. Epithelial cells nearest those anchors were assigned to that villus, and the previously calculated y-axis coordinates were used to assign each cell a position on that crypt-villus axis."

3. The major characterization in the intestine is that of transcript zonation along the villus axis with implication for function. There are couple of issues that should be discussed to qualify the interpretation. Firstly, although largely spatially arranged, maturation/differentiation and lineage trajectory is not exclusively positional – this is particularly the case for Paneth cells which are not part of the enterocyte maturation path, as well as several of the rarer secretory cells analyzed which may have discrete lineage branch points. Secondly more broadly, transcript (especially for transporters and channels) is often not correlated with functionally localized protein – this is less of an issue at the extremes (villus tip and crypt base) but in the intermediate regions.

That is an excellent point. We clarified the initial discussion of this analysis in the results section by specifying that we are focusing on enterocyte development. We added additional discussion on p.12, l.535–547.

"One limitation of SMURF's unrolling algorithm is that, while it enabled novel biological results that could be validated using published data in the intestine, it is difficult to rigorously benchmark how well SMURF's unrolling algorithm resolves the fine structure of tissues because there are few gold standard reference datasets for such analysis. Despite this limitation, we show that SMURF's output is highly consistent with biological expectations. Another caveat with our analysis is that spatial zonation of mRNA transcripts does not always correspond directly to lineage progression or protein-level function. For enterocytes, maturation and zonation are broadly aligned, but for some secretory cell types, such as Paneth cells, this relationship is obfuscated by the retrograde migration of these cells. In addition, spatial transcriptomic data measures RNA and not protein abundance and these are not perfectly correlated. Our analysis should therefore be interpreted as describing the zonation of transcript abundance rather than protein function."

a. The statistics and experimental details in Figures 3-5 need to be more clearly described. Exactly what was analyzed was often unclear. E.g. the number of tissue structures analyzed for each experiment. The error bounds for the expression levels in Figure 5d as an example (and others) are missing.

We apologize that the statistical and experimental details for Figures 3–5 were not sufficiently clear. In the revised manuscript, we expanded and clarified a dedicated *"Statistics and Reproducibility"* section in the Methods (p. 25, l. 1092-1103) and further revised the relevant Methods text and figure legends to specify the sample sizes, what was analyzed in each panel, and how uncertainty was estimated. The revised Methods and figure legends now clarify the sample sizes, what was analyzed in each panel, and that the error bounds in the zonation plots

represent 95% bootstrap confidence intervals for mean expression across cells within each zone, reflecting within-section cell-level variability.

b What are the limitations of the small number of UMI per cell. One of the big issues with spatial transcriptomics, as the authors point out in the small intestine, is that median number of UMI per cell is 165. The authors should comment on limitations on any analysis including theirs. Supplementary 1 which starts to look at this analytically is helpful (mentioned below) and further insights /discussion would be useful as the authors have clearly thought about this issue. This is especially important for any biological insights.

We appreciate the reviewer's comment on the limitations imposed by low UMI counts in high-resolution spatial transcriptomics. We agree that this issue is important for both our method and the interpretation of downstream biological results. In the revised manuscript, we now state more explicitly in the Discussion that the sparsity of high-resolution spatial transcriptomic data can limit sensitivity and introduce additional uncertainty into biological interpretation. We also expanded Supplementary Note 1 to further discuss this issue, emphasizing that sparse reconstructed single-cell profiles reduce sensitivity for lowly expressed genes and make subtle differences in cell state or gene expression more difficult to resolve robustly. At the same time, we clarify that SMURF can partially mitigate these limitations by incorporating transcripts beyond the nucleus and leveraging spatial continuity, but it cannot recover molecular signal that was not captured experimentally.

Specifically we added the following text to the main text (Discussion p. 11-12, l. 529–534).

"Another limitation inherent to sequencing-based ST datasets like those produced by Visium HD is that the low number of UMIs captured per cell limit sensitivity to detect lowly expressed genes and increase uncertainty in downstream analyses, including annotation of rare populations or closely related subtypes because of marker dropout (Supplementary Note 1)."

And in the Supplementary Note 1 (p. 15, l. 304–313):

"These analyses further highlight a general limitation of high-resolution spatial transcriptomics: reconstructed single-cell profiles remain sparse because only a limited number of transcripts are captured per cell. As a result, sensitivity for lowly expressed genes is reduced, zero counts and dropout remain common, and subtle differences in cell state or gene expression may be harder to resolve robustly. Although SMURF improves effective cell-level representation by incorporating transcripts beyond the nucleus and leveraging spatial continuity, it cannot recover molecular signals that were not captured experimentally. Therefore, downstream biological interpretation should be made with appropriate caution, particularly for low-abundance genes and subtle intermediate states."

c. Given the low UMI per cell, I am curious to know about the limitations associated with cell annotations?

We thank the reviewer for this important question. We agree that low UMI counts can limit the resolution and confidence of cell annotation, particularly for rare populations and closely related subtypes. In the revised manuscript, we now clarify this limitation more explicitly. Specifically, we note that low UMI counts can lead to marker dropout and make it more difficult to distinguish subtle subclusters whose annotations depend on a small number of lowly expressed genes. We mitigated this issue in several ways. First, during preprocessing we applied stringent quality control to remove low-count and poor-quality cells. Second, our annotations were based on cluster-level expression patterns and spatial context rather than single-marker expression alone, which makes major cell-type assignments more robust despite sparse counts. Third, the large number of reconstructed cells in Visium HD provides strong aggregate signal at the cluster level, and the resulting broad cellular composition is consistent with established scRNA-seq atlas data and known spatial organization. We therefore view low UMI counts as primarily limiting fine subtype granularity and confidence for rare or closely related states, rather than broad annotation of major cellular classes.

We added the following text to the discussion to address this point (Discussion p. 11-12, l. 529–534):

"Another limitation inherent to sequencing-based ST datasets like those produced by Visium HD, is that the low number of UMIs captured per cell limits sensitivity to detect lowly expressed genes and increase uncertainty in downstream analyses, including annotation of rare populations or closely related subtypes because of marker dropout (Supplementary Note 1)."

How many cells were not annotated because of the lack of data.

All cells that passed quality control were clustered and assigned an annotation. Cells were therefore not left unannotated after clustering because of sparse counts alone. However, we removed 28,383 cells (7.0% of the initial 404,382 cells) during quality control because they had low counts and/or poor-quality metrics, and these cells were not included in downstream annotation analyses. We now added the following clarification in the Methods p. 21, l. 882–884:

"After quality control filtering, 375,999 of 404,382 reconstructed cells were retained for downstream analysis and annotation, whereas 28,383 cells (7.0%) were removed because of low-count and/or poor-quality metrics."

Also, what was the overall cellular composition of their tissues and how does this compare to scRNA-seq atlas data.

Comprehensive atlas data is available from the mouse brain. We compared the abundance of major cell types in the mouse brain detected by SMURF to a reference Mouse Brain Atlas using MapMyCells tool [9]. As shown in Extended Figure 1b, the composition of cell types was very similar between SMURF and the reference scRNA-seq atlas.

To our knowledge, there is no single reference atlas for the mouse intestine that contains abundance data for all of the epithelial and non-epithelial cell types. While there are many excellent scRNA-seq studies from the mouse intestine, few are directly matched in terms of anatomical region, processing conditions, and cellular focus. A particular challenge in the intestine is that different cell compartments require distinct dissociation protocols and subsequent library preparations so that the abundance of cells across compartments can't be directly compared (e.g epithelium, stroma, and immune compartments).

To validate SMURF results in the intestine, we focused on the most abundant cellular compartment, the epithelium. To derive an estimate of expected epithelial cell type abundance, we collated data from 3 studies: Haber et al [10], Zwick et al [11], and Xu et al [12]. Haber performed scRNA-seq from the entire small intestine (SI) using a crypt enriched isolation protocol. Zwick performed 10x scRNA-seq from multiple contiguous SI segments using a total epithelial cell isolation strategy. Xu studied the mouse ileum using a custom MERFISH probe panel. Allowing for variance in experimental conditions and computational analysis, the epithelial cellular composition in our SMURF analysis was highly concordant with these reference atlases.

We have added 2 tables in Supplementary Note 1 documenting this analysis. The first presents the overall cellular abundance of all major cell types in the SMURF analysis. The second presents the epithelial cell abundance of SMURF compared to the reference atlases. We have updated the main text as follows (p. 5, l. 235-237):

"The calculated abundance of epithelial cell-types using SMURF assignments was broadly consistent with previous scRNA-seq and ST intestinal surveys (Supplementary Table S6)"

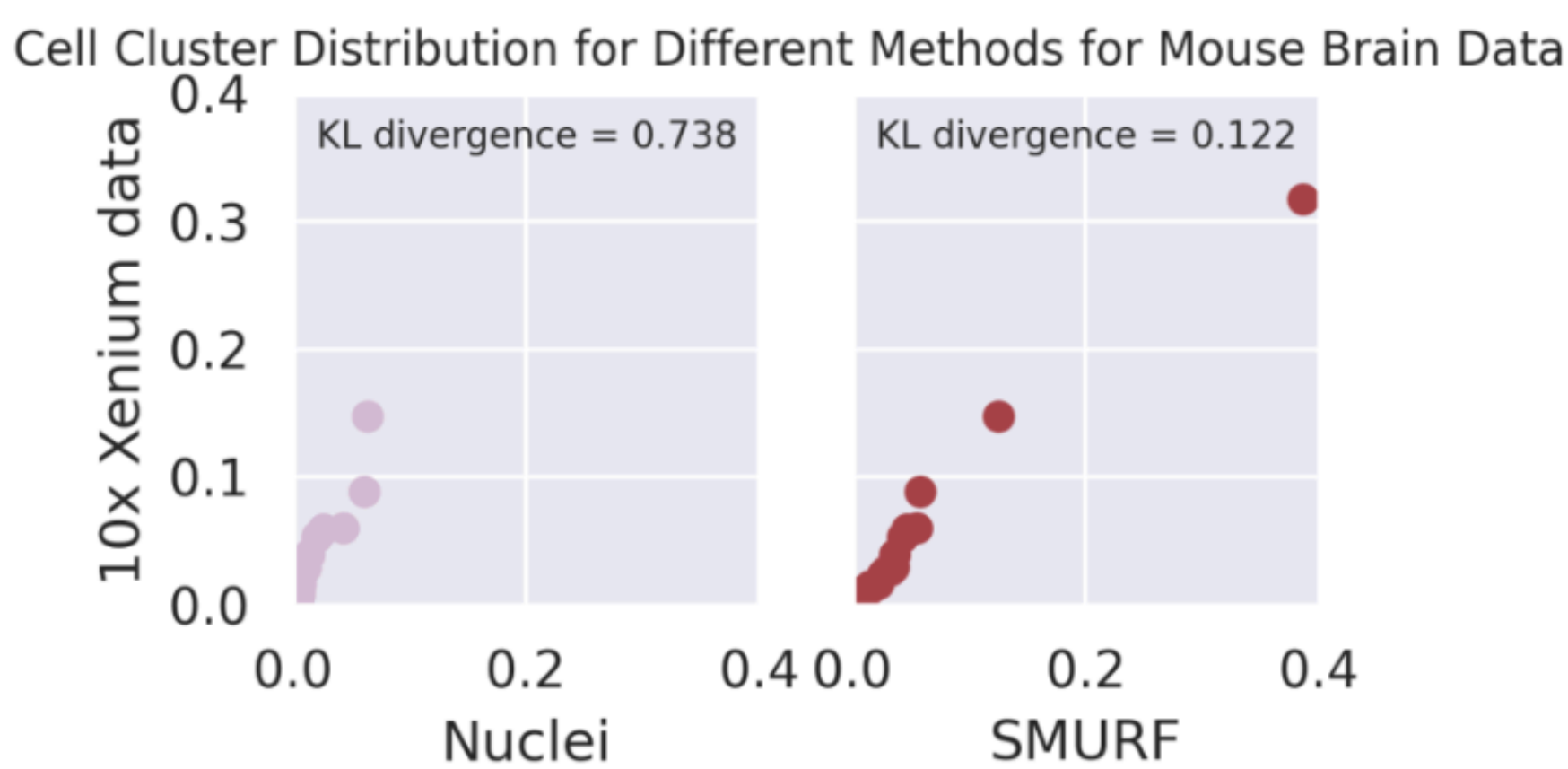

Extended Data Figure 1b: Cell type proportions annotated by MapMyCells in probe-based mouse brain data, comparing nuclei results and SMURF results. KL divergence values quantify differences in cell type distributions. Lower KL divergence indicates better concordance.

Table S5: Cell-Type Composition in the Visium HD Dataset.

Cell type	n	percent
Enterocytes	90145	23.97
Immune cells (excluding Peyer's patches)	57489	15.29
Smooth Muscle cells	45290	12.05
Peyer's patch	42875	11.40
Fibroblasts	36940	9.82
Paneth and Stem cells	30589	8.14
Goblet cells	24061	6.40
Transit Amplifying cells	18903	5.03
Endothelial cells	9850	2.62
Enteroendocrine cells	6365	1.69
Tuft cells	4677	1.24
High-mito cells	4648	1.24
Neurons	4167	1.11

Table S6: Comparison of Intestinal Cell-Type Proportions across Studies. Values Are Percentages. NR: Not Reported.

	Cell type proportion (%) by study			
Cell type	SMURF	Haber et al.	Zwick et al.	Xu et al.
Enterocyte	51.6	44.6	63.5	61.0
Paneth and stem	17.5	21.2	8.0	27.9
Goblet	13.8	7.1	11.7	9.7
Transit-amplifying	10.8	20.6	7.1	NR
Enteroendocrine	3.6	4.3	4.1	0.3
Tuft	2.7	2.3	5.6	1.1
Platform	Visium HD	scRNA-seq	scRNA-seq	MERFISH
Tissue / source	Ileum	Crypt isolation, SI	Total epithelium, SI	Ileum

4. Misc Findings: The authors should be commended as there are several important technical findings that are often taken for granted but have been explicitly tested here.

We thank the reviewer for the positive feedback and for recognizing the technical rigor of our statistical evaluations.

Using Negative Binomial to fit spatial data and comparison with various other distributions.
a. I think this is an important section and I would like to see this highlighted more in the main text.

Thank you for this positive comment. We agree that this is an important technical finding. In the revised manuscript, we now highlight more explicitly that reconstructed high-resolution spatial single-cell data are better described by a negative binomial model than by Poisson-based alternatives, and we direct readers to the Supplementary Note 1 for the full comparison across count models. We have added the following to the main text (p.5, l.197–200)

"Reconstructed high-resolution spatial single-cell counts were also more accurately modeled by a negative binomial distribution than by Poisson-based alternatives, consistent with substantial overdispersion in these data (Supplementary Note 1, Supplementary Figures S9-S12)."

Normalization artifact in the 8 µm method: I think is important especially given that the default binning is 8x8 in10X Visium and has been used in number of papers. I think the artifacts if detailed better will be useful to the community.

Thank you for highlighting this point. We agree that the normalization artifact at the 8 µm binning scale is important, particularly because this is the default setting for 10x Visium HD and has been widely used. In the revised manuscript, we now describe this issue more explicitly in the main text (p. 5, l. 194-197): *"We also found that SMURF's cell-level normalization corrects a severe count imbalance artifact observed in transcript-sparse regions when using the default 8 µm method in Visium HD (Supplementary Note 1, Extended Data Figure 1a)."* and provide additional detail in the Supplementary Note 1 (section title: *Normalization artifact in the 8 µm method*, p. 17, l. 314-324) to clarify the nature of the artifact and its implications for downstream interpretation.

5. Minor points a. Figure 2, spelling of Brain in 2c.

Thank you, the typo has been fixed.

b. Figure legends in supplementary material should be expanded.

We have expanded the figure legends throughout the Supplementary Materials. They now provide more comprehensive details regarding experimental conditions, data processing steps, and the specific statistical metrics visualized in each panel.

c. Are the figures S5 and S6 repeated?

We apologize. Figures S5 and S6 were indeed accidentally duplicated. This has now been corrected.

d. How is the performance of the lite version to the GPU version? Where should one use the GPU version? Could the authors expand on the actual runtime of the two versions. I was unable to directly test the software.

Thanks for the question. We agree that these issues were not clear in our original manuscript. We have now compared the performance of SMURF-lite relative to SMURF in response to Reviewer 1, Question 1b. We have also benchmarked the runtimes of both SMURF and SMURF-lite. In the mouse brain dataset, it took 2.92h for SMURF and 1.69h for SMURF-lite. We have updated our Supplementary Note 1 Table S1 to include the runtime benchmarking. We have updated our README/tutorial in our Git repository so that readers can make an informed decision about which package to use.

Comments from Reviewer 2

A key aspect of benchmarking SMURF for readers to make a decision on whether to implement it into their analysis will be to show Bin2cell results for the analysis in figure 2d-e (it seems only nuclear segmentation data is shown) and, more importantly, compare SMURF to the latest release of the LOUPE browser (v9.0 or later) that deployed this June, as the approach SMURF and 10x takes seems similar. Additionally, how long does it take to run SMURF vs LOUPE vs Bin2cell? What are the processing and data storage requirements?

We agree with the reviewer's comment that further benchmarking was needed, especially for the Space Ranger package. As detailed in our response to main theme 1, we have now benchmarked SMURF against additional packages including Space Ranger and substantially revised the manuscript to include these analyses.

With regard to some of the more specific questions posed above, we have now included the Bin2cell results as requested in the Supplementary Note 1. The relevant figure is pasted below (and the details of this analysis can be found in Supplementary Note 1, section title: *Comparison of cell-type annotation across transcript-assignment methods*, p. 23, l. 410–431). Bin2cell did not robustly resolve tuft or enteroendocrine cells, whereas SMURF reliably detects these cell types. Neither Space Ranger nor Nuclei Segmentation methods detected these cell types. We do note that all methods have some bleed-over for enterocyte specific genes across multiple clusters, although this issue is less acute for SMURF. In summary, this analysis reveals SMURF to be more sensitive and specific than both Space Ranger and Bin2cell.

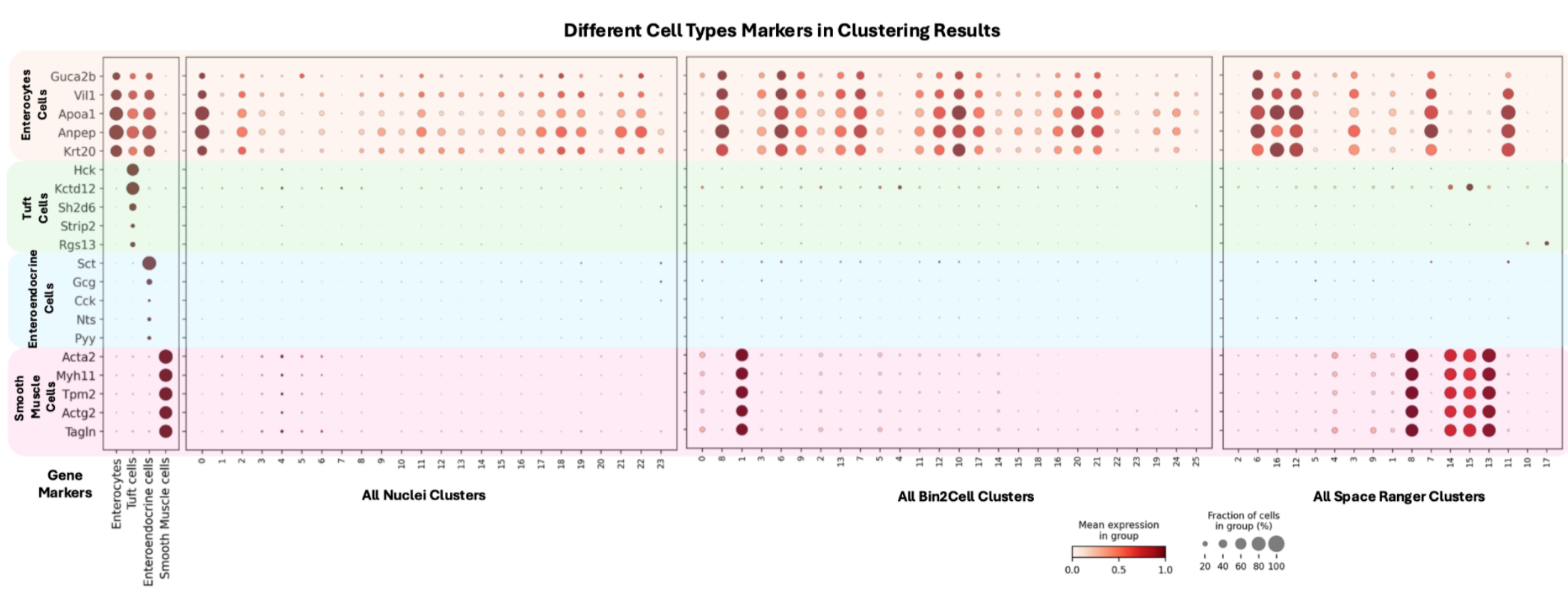

Fig. S17: **Marker-based comparison of clustering results across transcript-assignment methods in the mouse small intestine dataset.** Rows show canonical marker genes for enterocytes, tuft cells, enteroendocrine cells, and smooth muscle cells. Columns show clusters identified from SMURF, nuclei segmentation, Bin2cell, and Space Ranger outputs. Rare epithelial marker signatures, particularly for tuft cells and enteroendocrine cells, were weak or not cleanly resolved across these alternative methods.

With regard to data storage requirements, this depends on the specific dataset and analysis settings, including the size of the Visium HD 2 µm bin outputs, associated histology image files, and any external image-segmentation results provided by the user. Storage needs also depend on whether high-resolution visualizations and intermediate outputs are generated and saved. In our experience, processing a typical mouse brain dataset with SMURF requires approximately 25 GB of available storage. However, for datasets with larger numbers of cells and for analyses that include saving high-resolution visualizations and intermediate files, we recommend having on the order of 100 GB of storage available. We have added the following text to the main text on p.26, l.1133–1137 and in our Git tutorial.

"Storage requirements depend on the dataset and analysis settings, but in our experience, SMURF can typically be run on a mouse brain dataset with 25 GB of available storage, whereas larger datasets or analyses that retain high-resolution visualizations and intermediate files may require on the order of 100 GB."

The authors mention broad applicability of their method to deconvolute other tissues and data types (such as spatial metabolomics or proteomics). What about other imaging data? Could the authors demonstrate this process for immunostaining, for example, to further expand the utility of their work?

Thank you for this comment. We are excited to expand SMURF to other types of spatial technologies. Unfortunately, publicly available data from spatial proteomic or spatial metabolomic platforms is more limited than spatial transcriptomics data. We did, however, identify a study that used CODEX proteomics [13] in the mouse intestine [14]. We analyzed that data and added the following section (*Demonstration of the SMURF unrolling framework on spatial proteomics data*) as Supplementary Note 1 (p. 27, l. 468-481).

"We sought to evaluate whether SMURF's unrolling algorithm could be applied to non-transcriptomic spatial data. We identified a published mouse intestine spatial proteomics dataset generated using CODEX. We used the publicly available data provided with that study [35], including cell locations and antibody-intensity measurements. The paper provided public data for 10,000 of the 150,793 total cells in the form of cell locations and antibody staining intensity. The authors manually traced the base of the intestine. Using the authors' reported trace as the pseudo-x axis base in SMURF, we were able to assign x-y coordinates to the other cells (Supplementary Figure S18). Although the available data was sparse, the cells in the image were placed on appropriate x and y coordinates reflecting their proximal-distal and crypt-villus location. These results suggest that the unrolling framework may be adaptable to protein-based spatial imaging data."

We have added the following sentence to the main text (p.12, l. 556-558): *"Finally, SMURF's unrolling algorithm will likely be adaptable to protein- or metabolite-based spatial technologies, although this will require further validation on high-quality datasets (Supplementary Figure S18)."* to

reflect that we have not fully deployed SMURF to proteomics or metabolomics yet but hope to in the future as this kind of data becomes more available.

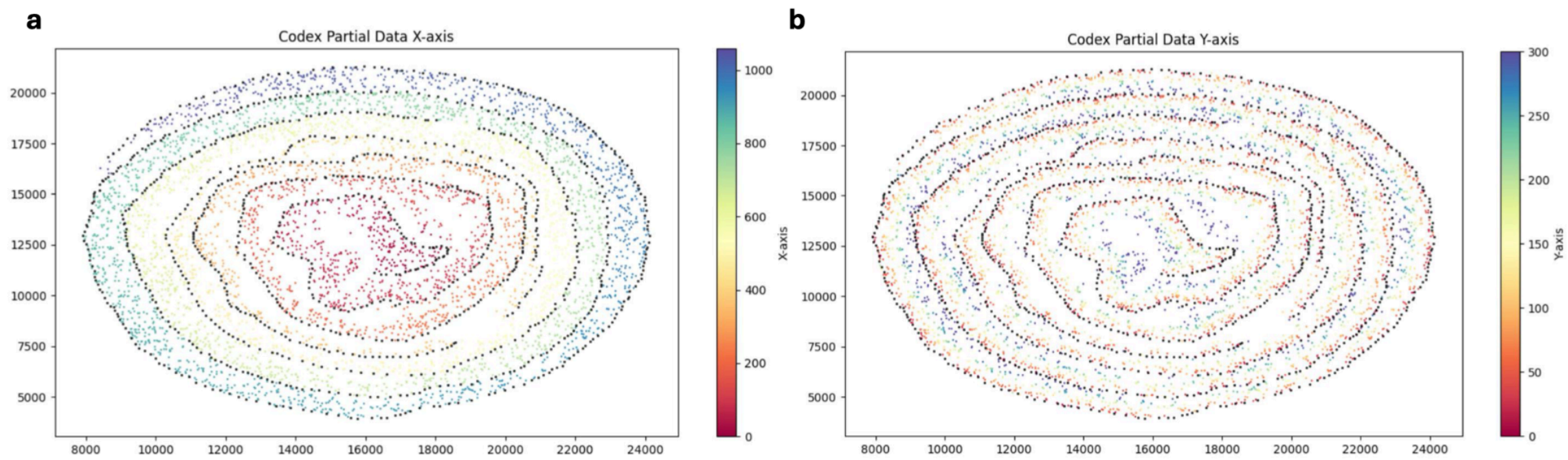

Fig. S18: Exploratory application of the SMURF unrolling framework to a published mouse intestine CODEX dataset. Using the author-reported intestine base trace as the pseudo-x-axis base, cells were projected into an unrolled coordinate system. The resulting layout was broadly consistent with expected proximal-distal and crypt-villus organization. This analysis is intended as a proof-of-concept visualization rather than a validated deployment of SMURF to spatial proteomics data.

Minor:
The utility of SMURF to make new discoveries is fairly limited within the current paper, though an attempt to do so is provided in figure 6. This seems reasonable given the paper's focus on the analysis tool. The villus tip repatterning after resection is fairly modest, with only 34 genes differentially expressed in their re-analysis of published scRNAseq data. The authors do mention important caveats to their findings though, so this concern is modest.

We appreciate that the reviewer felt we appropriately discussed the caveats to our study. To briefly clarify, we identified 77 genes that were differentially expressed in the intestinal resection data using stringent filtering criteria. 72 of them were present in the ST data, and all of them are shown in Figure 6 where they exclusively localize in the upper villus (Zones 4, 5, and 6). 32 of the differentially expressed genes localized in Zone 6.

More broadly, these comments were very helpful as we re-evaluated our description of the results in this section. We intended to communicate that regionally specific genes are accumulated in the upper parts of the villus which are in closest contact with luminal environmental factors. We often used the term “tip” interchangeably with “upper villus” as we described our results. In the context of the rest of our paper and the established literature, we realized that our use of the word “tip” here placed unintended emphasis on Zone 6 cells. We have replaced the word “tip” in this section with “upper villus”, “top”, or similar terms. In the remainder of the manuscript, we now restrict the term “tip” to discussion of Zone 6.

Comments from Reviewer 3

However, the robustness of SMURF seems to depend strongly on the accuracy of nuclei segmentation, while quantitative evaluation of segmentation specificity remains limited. In addition, broader and more systematic benchmarking against state-of-the-art methods would be necessary to firmly establish SMURF as a reliable standard for single-cell–resolution spatial transcriptomics. Strengthening the methodological validation would substantially enhance the impact and generalizability of the framework.

We thank the reviewer for this thoughtful summary. We performed extensive additional analyses to address these points. These results and our subsequent additions to the manuscript are described in detail in our response to Major Theme above.

Major comments:

1. The manuscript convincingly shows that SMURF recovers more UMIs per cell than nuclei-based approaches. However, increased sensitivity may come at the expense of specificity. Could the authors quantify potential transcript "bleed-over" or contamination? A negative control analysis assessing how frequently cell-type specific markers are incorrectly assigned to neighboring cells—relative to rigid nuclei segmentation—would help evaluate the trade-off introduced by the soft segmentation strategy.

We appreciate the reviewer's concern that the increased sensitivity of SMURF to detect UMIs might lead to an increase in the erroneous assignment of transcripts from one cell type to another relative to nuclei segmentation.

The reviewer identifies an important limit of ST technologies, in that RNA from one area of the tissue section can diffuse into neighboring spaces. This is one form of bleedover or "spot-swapping" that is unavoidable in ST approaches [15,16].

Because some bleed-over is unavoidable, the most relevant question is: does SMURF have more "bleed over" than nuclei segmentation. A real world test of this is to measure whether one cell type has markers of another cell type using output from rigid nuclei segmentation compared to SMURF segmentation. We tested this using the mouse brain dataset and assigned cell types using the Allen Brain Atlas MapMyCells tool [9]. As expected, cells called by SMURF soft-segmentation had greater sensitivity for canonical cell-type marker genes than nuclei segmentation alone (higher average expression) . Surprisingly, the SMURF segmentation was also more specific than nuclei data alone. SMURF assignments had fewer examples of erroneous marker gene detection in each cell type than the nuclei data. This was true across 4 different neuronal cell types.

This result may seem counterintuitive. We interpret this to mean that SMURF's increased sensitivity for assigning UMI to cells leads to more accurate clustering of a given cell into its type. This real world example illustrates how sensitivity and specificity are intrinsically linked,

because specificity depends on the accurate assignment of “cells” (or nuclei) to their type-resolved cluster, which in turn requires that UMIs are detected with high sensitivity. We thank the reviewer for suggesting this analysis as we did not anticipate this unappreciated link between the sensitivity and specificity of spatial transcriptomic analysis methods. We have included this analysis in Supplementary Figure S8 and added the following comment to the Supplementary Note 1 (p.11-12, l.217-239).

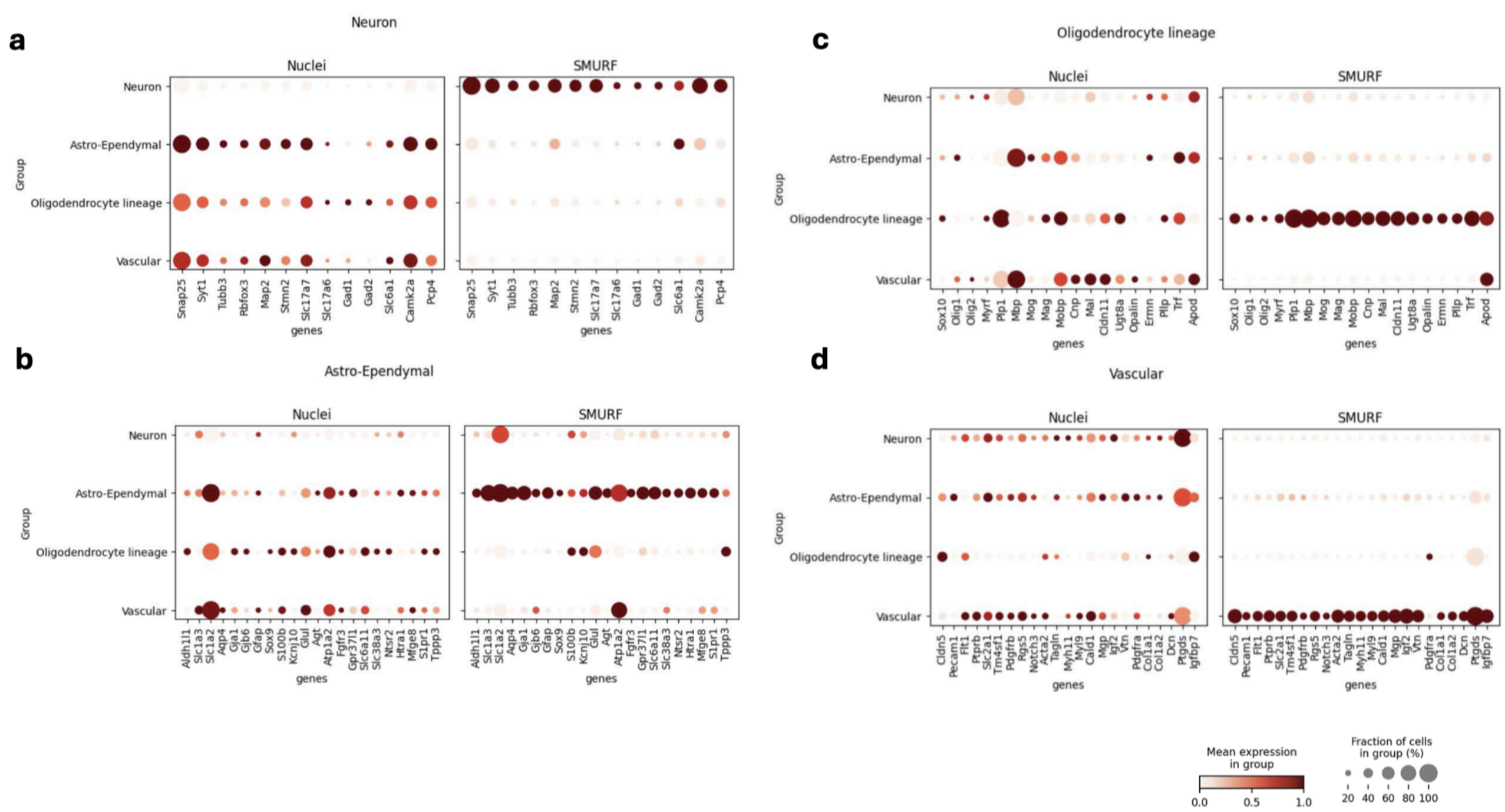

Fig. S8: Assessment of cross-cell-type marker contamination in SMURF versus nuclei-only segmentation in the mouse brain dataset. (a–d) Dot plots showing the expression of canonical marker genes for neurons (a), astro-ependymal cells (b), oligodendrocyte-lineage cells (c), and vascular cells (d), comparing cells defined by nuclei-only segmentation and SMURF segmentation. Dot size indicates the fraction of cells expressing each gene, and color indicates mean expression level. Marker specificity was compared across major cell groups to assess whether SMURF expansion increased erroneous cross-cell-type transcript assignment.

“A general limitation of sequencing-based spatial transcriptomics is that transcripts may diffuse into nearby locations, producing local bleedover or “spot swapping” between neighboring measurement units. To determine whether SMURF-based cell expansion increases erroneous assignment of transcripts across cell types relative to rigid nuclei segmentation, we evaluated marker specificity in the mouse brain dataset. Cell types were assigned using the Allen Brain Atlas MapMyCells tool, and we compared the expression of canonical marker genes across matched cell populations defined by nuclei-only segmentation or SMURF segmentation. As expected, SMURF-assigned cells showed higher overall marker-gene detection than nuclei-only segmentation, consistent with an increased sensitivity due to broader transcript recovery. However,

SMURF segmentation did not show an increase in cross-cell-type marker contamination. Instead, across the four neuronal cell types examined, SMURF-assigned cells showed fewer instances of erroneous marker-gene detection than nuclei-only segmentation (Supplementary Figure S8).

These results suggest that the increased transcript recovery obtained by SMURF improves effective cell-level representation without increasing cross-cell-type contamination in this setting. One possible interpretation is that the higher sensitivity of SMURF leads to more accurate clustering and cell-type assignment, thereby improving both marker detection and marker specificity. Thus, in this empirical comparison, SMURF achieved a more favorable balance between sensitivity and specificity than rigid nuclei segmentation alone."

2. The current benchmarking focuses on comparisons with rigid binning and nuclei-only segmentation. To better position SMURF among state-of-the-art methods, it would be valuable to include comparisons with existing probabilistic molecule assignment approaches adapted for high-resolution data (e.g., Baysor, JSTA, or Cellpose).

This question was addressed in our response to Major Theme above.

Additionally, for the deconvolution step (Figure 1), comparison with established deconvolution methods such as RCTD or Cell2Location (applied to small bins) would help justify the proposed deep learning formulation.

This is an excellent question, and we appreciate the chance to clarify this issue. RCTD [17] and Cell2Location [18] were developed to deconvolve spatial transcriptomic measurements that contain mixtures of multiple cells of different cell-types, as is typical for lower-resolution platforms such as Visium [19] or Slide-seq [20]. In Visium HD, however, the 2 μm capture locations are smaller than most cells, so the deconvolution problem is different in an important way: many spots are expected to contain mRNA from a single cell or from adjacent cells of the same cell type, rather than from mixtures of clearly distinct cell types. For spots containing mRNA from different cell types, our SMURF algorithm uses a computational strategy that is highly similar to RCTD to deconvolve a spot into different cell types. The key additional challenge addressed by our deep learning formulation is the deconvolution of shared spots between adjacent cells <u>belonging to the same cell type</u>. Established cell-type deconvolution methods such as RCTD or Cell2Location are not designed to solve this problem. We have clarified this point in the revised manuscript.

We also note that practical limitations of applying Cell2Location to very high-resolution Visium HD data have been discussed by the authors and users of that package. Memory usage doesn't scale efficiently to very large numbers of capture locations and the authors don't recommend using Cell2Location for Visium HD data (https://github.com/BayraktarLab/cell2location/issues/358).

To clarify this issue, we edited the methods section (p.16–17, l.713–718) in the manuscript to more clearly indicate that we use a standard, least squares based deconvolution algorithm.

Demonstrating performance relative to state-of-the-art methods in common downstream tasks (e.g., spatially variable gene detection, spatial domain identification) would further strengthen the appeal of SMURF to potential user.

Thank you for this suggestion. We further evaluated method performance on the mouse brain dataset using a standard Squidpy workflow for spatial domain identification [21]. In the hippocampal region, SMURF was the only method that automatically resolved three anatomically coherent spatial domains, whereas the other methods generally recovered only two broader domains and merged finer hippocampal structures (Supplementary Fig. S3, also copied below for convenience). In particular, the other methods largely collapsed the hippocampal pyramidal layer into a single broad domain, while only SMURF was able to successfully separate the CA1/2 region from CA3. These results indicate that SMURF better preserves biologically meaningful spatial organization for downstream spatial domain identification. We have added this analysis to the Supplementary Note 1 (section title: *Comparison of downstream spatial domain identification across segmentation methods*, p. 6, l. 109–129) and cite it in the revised manuscript.

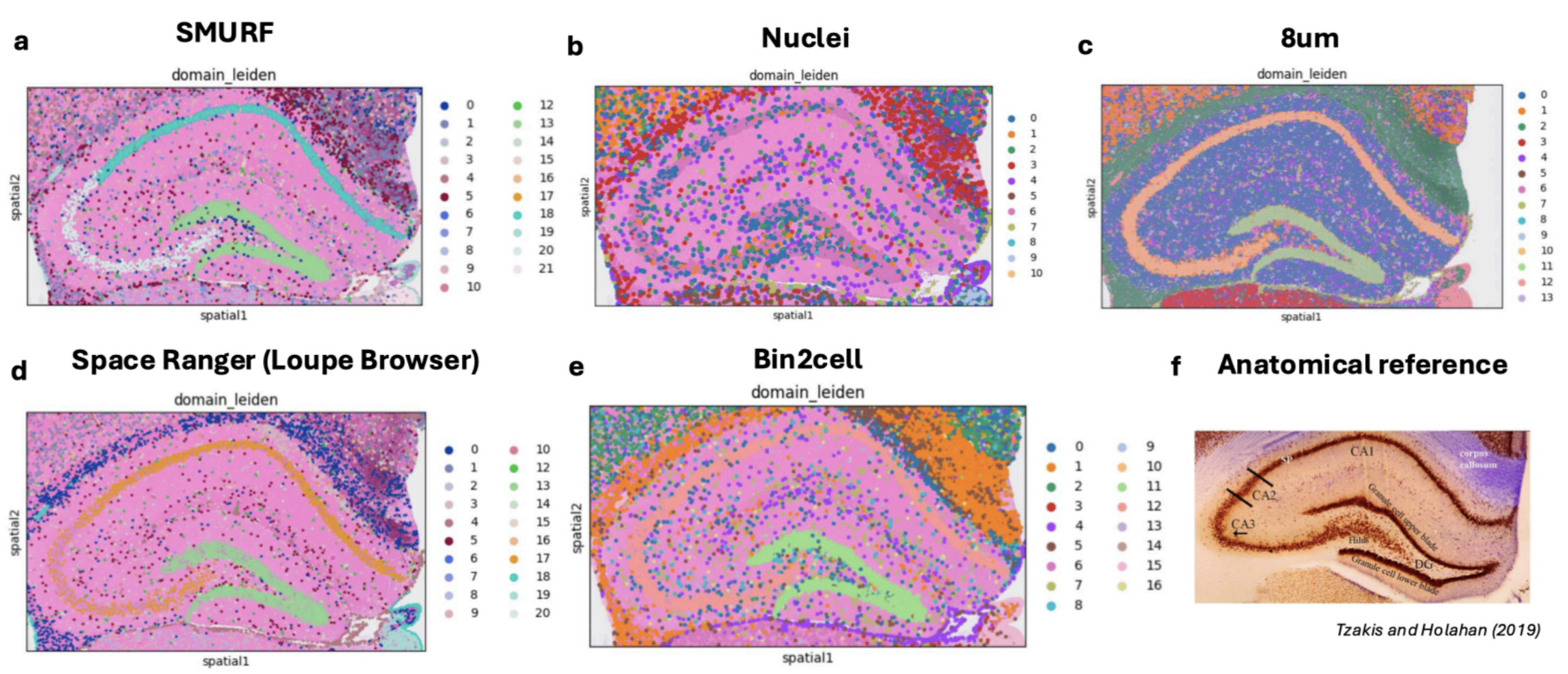

Fig. S3: Comparison of downstream spatial domain identification across segmentation methods in the mouse brain dataset using a standard Squidpy workflow. Spatial Leiden domains in the hippocampal region are shown for (a) SMURF, (b) nuclei-only segmentation, (c) 8 μm binning, (d) Space Ranger (Loupe Browser), and (e) Bin2cell. All methods were analyzed within the same general downstream framework for neighborhood graph construction, dimensionality reduction, and spatial domain identification. (f) Annotated anatomical reference image of the dorsal hippocampus, adapted from Tzakis and Holahan et al. , showing major subregions including CA1, CA2, CA3, dentate gyrus (DG), hilus, and the granule cell upper and lower blades.

We also investigated the use of Squidpy as a downstream analysis to detect spatially variable genes using the output of SMURF and the other methods. Our analysis reveals that they all give highly similar results (with the exception of nuclei segmentation, which performs poorly). However, we believe that SMURF's unrolling capability goes beyond Squidpy and similar methods because they ignore the added dimension of information provided by 2-D spatial transcriptomics analysis methods. For example, as described in our manuscript, SMURF revealed that the proximal to distal changes in gene expression occurred primarily in tip cells and not at the crypt base. This spatial dimension (crypt to villus) is completely ignored with standard packages like Squidpy.

3. The method introduces a deep learning module to resolve spots shared across multiple cells. Given the additional computational complexity, it would be helpful to clarify the specific contribution of this component. An ablation analysis quantifying the performance gain attributable to this module, relative to the soft-segmentation strategy alone, would help justify its inclusion in the pipeline.

We agree that it is important to quantify what the deep learning step in SMURF contributes to overall accuracy. We should clarify one point that was not clear in our original manuscript. SMURF supports two operating modes that differ in how these shared spots are handled: a "full" mode (deep learning-based; typically requiring a GPU for practical runtimes) and a "lite" mode (non-deep-learning; runnable on a standard workstation and uses a spatial distance weighted random distribution to assign transcripts). So to perform the ablation analysis as the reviewer suggests, we can compare the performance of SMURF versus SMURF-lite (since we have "ablated" the deep learning component of SMURF in SMURF-lite). To make this comparison, we computed the cosine similarity between the mean cluster profile and cells in the cluster, for all cells assigned transcripts by the deep learning algorithm in SMURF (the full version) or by using the classical deconvolution algorithm in SMURF-lite. A paired t-test shows that cells assigned transcripts by SMURF have a significantly higher cosine similarity to their parent cell type than those assigned transcripts by SMURF-lite (see Figure below).

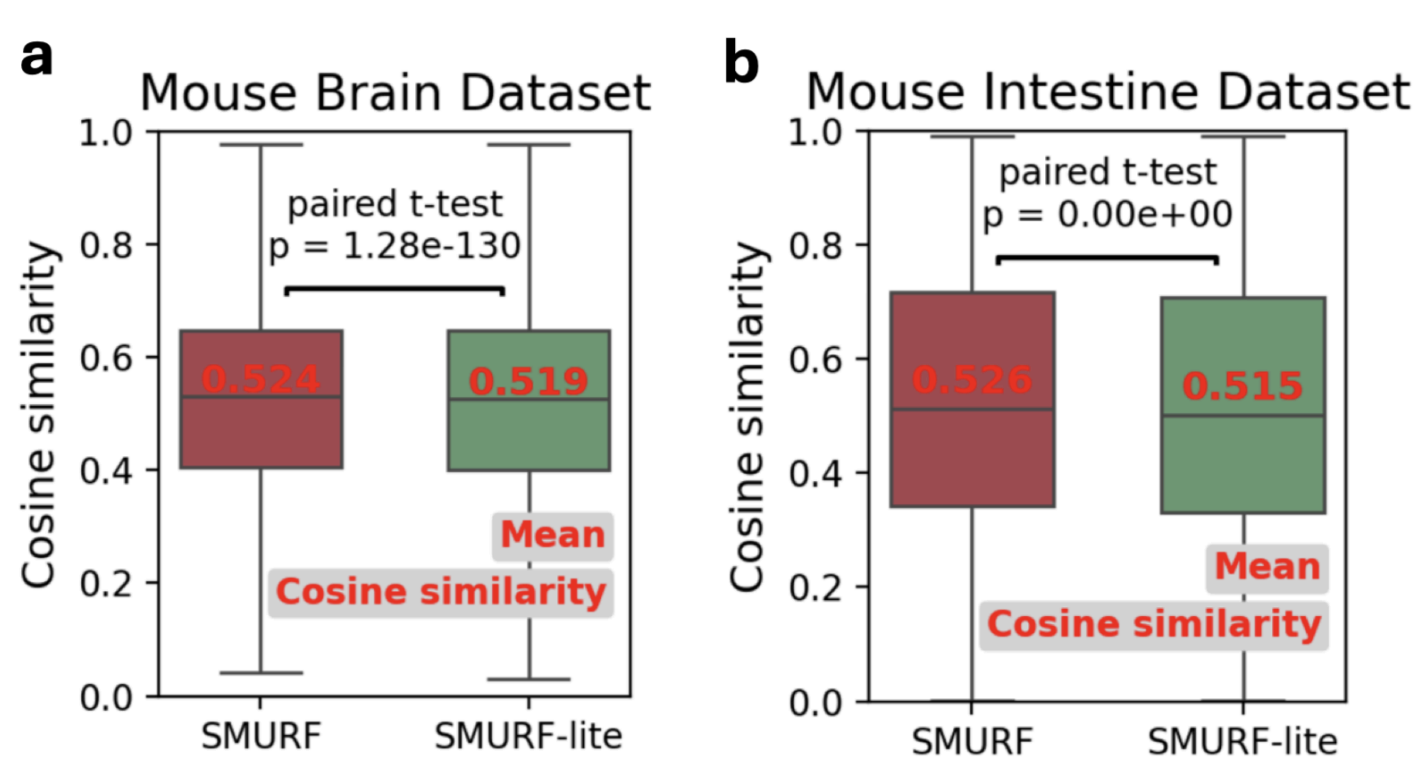

Fig. S6: Comparison of cosine similarity between reconstructed cells and the mean expression profile of their assigned clusters for SMURF and SMURF-lite. (a) Mouse brain dataset. (b) Mouse small intestine dataset. Red numbers indicate mean cosine similarity. P values were calculated using paired t-tests.

We have updated the manuscript to make the availability of SMURF-lite more clear, and we have added the analysis just described to Supplementary Figure S6 and added a more detailed description in the Supplementary Note 1 (section title: *SMURF vs. SMURF-lite: evaluating the contribution of the deep learning module*, p.9, l.172–196).

Minor comments:

1. The methods state that the analysis focuses on regions covered by spots, which may include cells only minimally overlapped by a spot. Could the authors clarify whether the method can handle cells that are entirely uncovered by any spots? If so, highlighting this capability would strengthen the manuscript.

Thank you, this was confusing. In some datasets, the tissue section can extend beyond the Visium HD capture area; conversely, some spots may not be covered by a tissue section if the geometries do not match. We were expressing that we only considered areas where spots and cells overlap. We have updated the methods (p. 12, l. 584-585) to clarify this point *"We limited our analysis to the section of the tissue bounded by the Visium HD capture area"*.

2. A minimum overlap fraction of 1/6 is used when assigning shared spots to cells. Please clarify how this threshold was chosen and whether the results are sensitive to this parameter.

We agree that this was not sufficiently clear in the original manuscript. The minimum overlap fraction helps decide the initial starting point for SMURF's transcript assignment. The value of 1/6 is the initial default value, and can be adjusted by the user. Because SMURF uses an expectation-maximization algorithm to assign spots to cells, and generally these are not too sensitive to the initialization state, we expect that the final results aren't highly sensitive to this parameter. To test this, we repeated the analysis using nearby thresholds (1/5 and 1/7) and found that the resulting UMAP embeddings and cell-type assignments were consistent across settings, even for the initial cluster assignments. We have added these comparisons to the Supplementary Note 1 (section title: *Sensitivity analysis for the minimum overlap fraction used in shared-spot initialization*, p. 11, l. 197–216) and now clarify in the Methods (p. 15, l. 627–628) that this parameter is user-adjustable and can be tuned for tissues with different nuclear density or imaging resolution. We have added the following text:

"To initialize transcript assignment for shared spots, SMURF uses a minimum overlap fraction threshold to define candidate cell–spot relationships at the start of the optimization. Because this parameter affects only the initialization step, whereas final transcript assignment is refined by the downstream expectation–maximization procedure, we expected SMURF to be relatively insensitive to modest variation in this setting.

To assess the sensitivity of SMURF to the minimum overlap fraction used for initializing shared-spot assignment, we repeated the analysis using thresholds of 1/5, 1/6, and 1/7. Across these settings, the resulting low-dimensional structure and cluster assignments remained broadly consistent (Supplementary Figure S7). To quantify this, we compared Leiden cluster assignments

among cells shared across runs (n = 56,774 common cells). Pairwise normalized mutual information values were 0.520 for 1/5 versus 1/6, 0.505 for 1/5 versus 1/7, and 0.550 for 1/6 versus 1/7, indicating similar overall clustering structure across nearby parameter choices. In addition, the overlap of retained cells across runs was very high, with the shared-cell set comprising 99.09% of cells in the 1/5 run, 99.84% in the 1/6 run, and 99.80% in the 1/7 run. These results indicate that SMURF is not highly sensitive to modest variation in this initialization parameter."

3. Leiden clustering is used to generate initial clusters, but the resolution parameter can strongly influence the number of clusters. Could the authors specify the resolution value used in SMURF and explain whether it is fixed or dataset-dependent? Additional guidance on how this parameter is selected to optimize results would improve reproducibility.

We thank the reviewer for this helpful comment. In SMURF, the Leiden resolution parameter is dataset-dependent rather than fixed, because the appropriate clustering granularity depends on tissue complexity, cellular heterogeneity, and data quality. By default, we use a resolution of 2.0 for the initial clustering of the nuclei-based representation as the starting point for soft segmentation. This parameter is user-adjustable, and we selected a value that produced biologically coherent, stable, and interpretable clusters while avoiding obvious over-splitting or inappropriate merging of distinct populations, consistent with established best practices in single-cell RNA-seq and spatial transcriptomics analysis (e.g., Scanpy or Seurat). We have now clarified the default value, its role in initialization, and practical guidance for tuning it in the revised Methods (p. 14, l. 639-642) and repository documentation. We added the following text to the methods:

"For this initial clustering step, we use a default Leiden resolution of 2.0, although this parameter is user-adjustable and may be tuned according to dataset complexity and the desired clustering granularity. For these analyses, we followed standard Scanpy/Seurat best practices."

SMURF can be easily installed in a Python ecosystem and a clean and comprehensive documentation and tutorial is also available on the accompanying website.

We thank the reviewer for this positive comment and appreciate the recognition of our efforts to make SMURF accessible through straightforward installation, documentation, and tutorials.

Comments from Reviewer 4

1. There have been other methods that unrolls Swiss rolled intestines in spatial transcriptomics, for example: https://ludvigla.github.io/semla/articles/digital_unrolling.html and https://doi.org/10.1093/bioinformatics/btad140. It would be helpful to discuss how SMURF's unrolling relates to the existing unrolling methods, much like the discussions on bin2cell when it comes to assigning spots to cells.

Several previous publications have indeed used manual annotation of the muscle layer in intestinal Swiss roll images to cast ST or spatial proteomic data onto a pseudo-x axis. These studies have many strengths, but one key advantage of SMURF is that the base layer coordinates are detected automatically. We attempted to use the two suggested programs on the Visium HD data. We were not able to run Semla on our computer, as the software did not recognize our mouse clicks when we tried to select lines for the base layer [22]. We did run IntestLine on the Visium HD dataset, and directly compared the results of that program with SMURF. We have added the following text describing the results of this analysis and commentary in the Supplementary Note 1 (section title: *Comparison of SMURF unrolling with existing Swiss-roll unrolling methods*, p.21–22, l.372–397) [14].

"To further benchmark SMURF against existing Swiss-roll unrolling methods, we examined Semla and IntestLine. These methods were developed primarily for spot- or cell-based datasets and rely on user-guided geometric operations, such as manual graph editing or manual specification of a base layer. By contrast, SMURF is designed for high-resolution spatial data and infers unrolling coordinates from cell organization and clustering while preserving physical distance information. We attempted to apply Semla to our Visium HD dataset; however, the software's graphical interface was unresponsive to the manual inputs required to delineate the base layer, preventing us from successfully executing it in our local computing environment. We therefore performed a direct comparison between SMURF and IntestLine on the same mouse small intestine section. Because manual specification of the x-axis and center point is required in the original IntestLine workflow, these inputs were provided using the same tissue geometry as that used for SMURF. This ensured that the comparison reflected differences in method performance rather than differences in the initial geometric reference. No modifications were made to the IntestLine algorithm itself.

IntestLine was substantially slower on more than 400,000 cells (3 h 45 min versus 35 min) and produced x- and y-axis assignments that were less consistent with the expected tissue geometry (Supplementary Figure S15). For example, IntestLine often assigned villus cells to the wrong muscle layer, resulting in low y-axis values for villus cells that would be expected to have high values. In panel d-f, this behavior is visible in regions where red villus cells appear adjacent to red muscle cells despite being separated by luminal space. Together, these results suggest that SMURF is better suited for dense, high-resolution spatial datasets."

Together, these analyses clarify how SMURF relates to existing Swiss-roll unrolling approaches and support the conclusion that SMURF is better suited for dense, high-resolution spatial datasets.

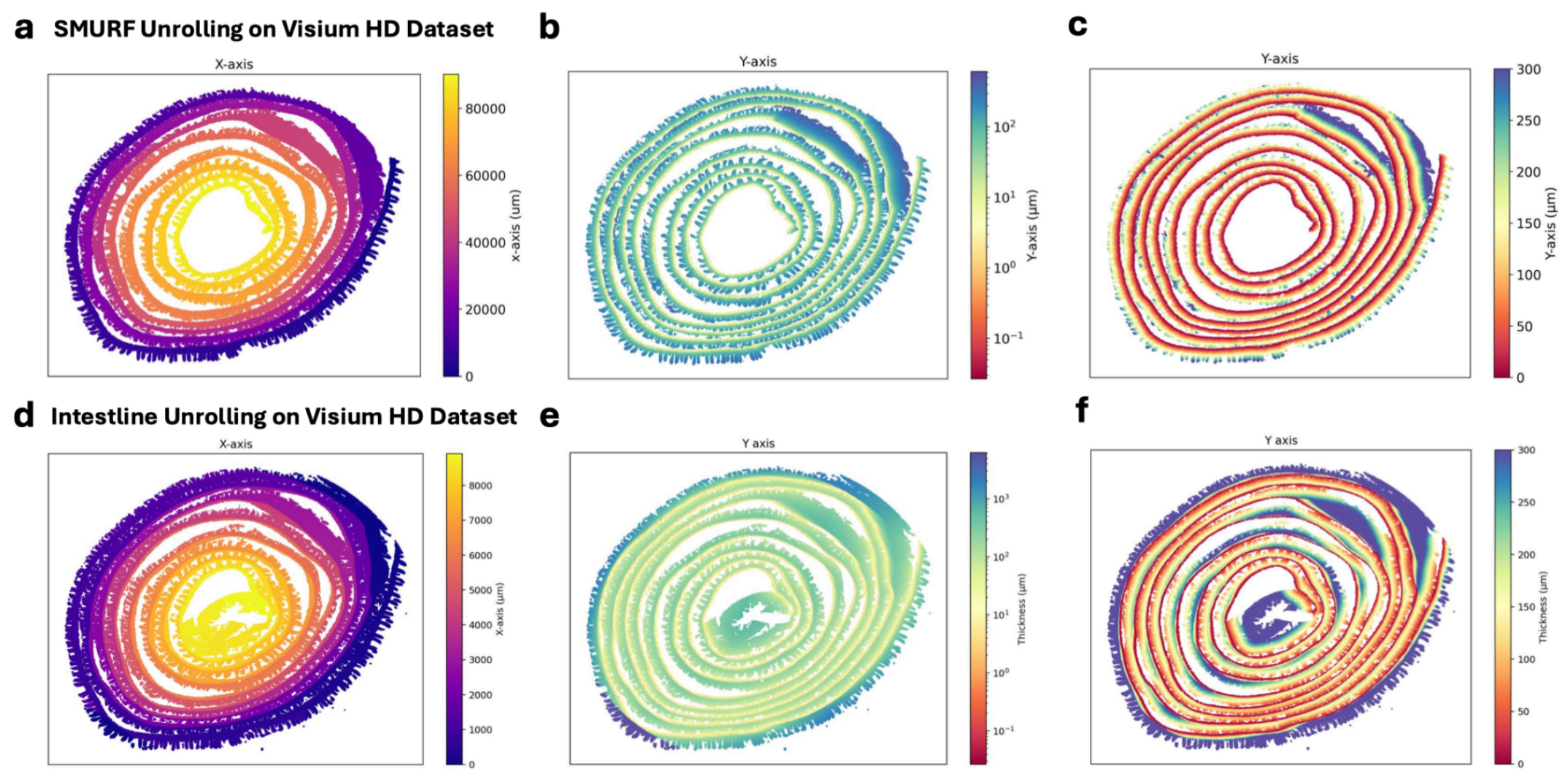

Fig. S15: **Comparison of Swiss-roll unrolling results obtained with SMURF and IntestLine on Visium HD mouse small intestine data.** (a-c) SMURF-derived unrolling coordinates, showing the x-axis coordinate in (a) and the y-axis coordinate on log and linear color scales in (b) and (c), respectively. (d-f) IntestLine-derived unrolling coordinates, obtained using the same SMURF-derived x-axis and center point as inputs, showing the x-axis coordinate in (d) and the thickness (y-axis) coordinate on log and linear color scales in (e) and (f), respectively. Overall, SMURF yielded unrolling coordinates that were more anatomically coherent than those produced by IntestLine on this Visium HD intestine section.

2. Regarding zonation of the villi, the villi were divided into 6 zones. Gene expression from the zones was pseudo-bulked and the Wilcoxon rank sum test was used to identify genes differentially expressed in each zone. What's the reason to use 6 discrete zones instead of the continuous values of the y-axis with a spatially variable gene method that accounts for spatial autocorrelation in this axis? And why 6 zones instead of a different number?

Thank you for this thoughtful comment. We agree that modeling gene expression continuously along the y-axis is a reasonable alternative. In the present study, we chose a discrete zonation strategy for two reasons. First, dividing the axis into six zones enabled direct comparison with established intestinal zonation literature, particularly the landmark study by *Moor et al* [8], in which the villus was partitioned using laser capture microdissection into five sub-zones in addition to the crypt (6 zones total, just as we do). This allowed us to benchmark our results to previous literature (Supplementary Figures S13-14). Second, discretization provided a simple and interpretable framework for pseudo-bulk comparison across spatial compartments in this initial demonstration.

We added the following text to our main text (p.9, l. 390-391):

"We chose 6 zones so that we could directly compare our results to a previous LCM study of jejunal zonation."

To assess whether our findings depended strongly on the chosen number of zones, we performed sensitivity analyses using 5 and 7 zones. The resulting gene expression trends and biological conclusions were highly consistent using the alternative binnings, indicating that our conclusions are not specific to the choice of six zones. We added a new section detailing these results to our Supplementary Note 1 (section title: *Sensitivity of intestinal zonation results to the number of spatial zones*, p.19–20, l.359–371).

3. Bin2cell normalizes Visium HD data row by row due to the stripy artifact at the 2 micron resolution. Do you find such normalization relevant or helpful to downstream analyses? What may be other Visium HD technical artifacts?

We thank the reviewer for this insightful technical question. Based on our analyses, we do not find row-by-row normalization to be generally helpful for downstream analysis of Visium HD data. In our view, variation in UMI counts at 2 µm resolution is driven primarily by biological differences in local cell and nuclei density, rather than by a consistent row-specific technical bias. As a result, row-wise normalization can be problematic in low-density or cell-free regions, where it may artificially inflate sparse counts and introduce downstream artifacts. This interpretation is also consistent with community discussion of Visium HD preprocessing, including reports that destriping procedures can in some cases exaggerate row/column-related effects in sparse data https://github.com/Teichlab/bin2cell/issues/45).

In contrast, we favor a segmentation-first strategy in which bins are first grouped into biologically meaningful cell units before normalization. We find that this better preserves true spatial heterogeneity while avoiding artifacts introduced by coordinate-based normalization. We have clarified this point in the revised manuscript (Supplementary Table S1, p. 5, l. 194–200). We also note that another important technical artifact in Visium HD is the normalization artifact observed at the default 8 µm binning scale, which we now discuss more explicitly in the Supplementary Note 1(section title: *Normalization artifact in the 8 µm method*, p. 17, l. 314-324).

Reviewer #4 (Remarks on code availability):

The package is available from pip and has a nice and detailed documentation website with instructions on installing and using the package.

We thank the reviewer for this positive comment and appreciate the recognition of our documentation and package availability.

Code Revision

We made a very small revision to the code base that clarifies how deconvolution handles transcripts shared by multiple cells in the deep learning algorithm. This did not change any of the major biological conclusions of the paper. All figures and tables reflect the output of this

code update. These code changes are reflected in the current stable Github/PyPI release and associated documentation.

References

1. Polański, K. *et al.* Bin2cell reconstructs cells from high resolution Visium HD data. *Bioinformatics* **40**, btae546 (2024).
2. 10x Genomics. Space Ranger Downloads. (2025).
3. 10x Genomics. Release Notes for Space Ranger. (2025).
4. Chen, H., Li, D. & Bar-Joseph, Z. SCS: cell segmentation for high-resolution spatial transcriptomics. *Nat. Methods* **20**, 1237–1243 (2023).
5. Petukhov, V. *et al.* Cell segmentation in imaging-based spatial transcriptomics. *Nat. Biotechnol.* **40**, 345–354 (2022).
6. Prabhakaran, S. Sparcle: assigning transcripts to cells in multiplexed images. *Bioinforma. Adv.* **2**, vbac048 (2022).
7. Littman, R. *et al.* Joint cell segmentation and cell type annotation for spatial transcriptomics. *Mol. Syst. Biol.* **17**, MSB202010108 (2021).
8. Moor, A. E. *et al.* Spatial reconstruction of single enterocytes uncovers broad zonation along the intestinal villus axis. *Cell* **175**, 1156–1167 (2018).
9. Allen Institute for Brain Science. MapMyCells (RRID:SCR_024672).
10. Haber, A. L. *et al.* A single-cell survey of the small intestinal epithelium. *Nature* **551**, 333–339 (2017).
11. Zwick, R. K. *et al.* Epithelial zonation along the mouse and human small intestine defines five discrete metabolic domains. *Nat. Cell Biol.* **26**, 250–262 (2024).
12. Xu, R. J. *et al.* An image-based transcriptomics atlas reveals the regional and microbiota-dependent molecular, cellular, and spatial structure of the murine gut. *Cell Host Microbe* (2025).
13. Goltsev, Y. *et al.* Deep profiling of mouse splenic architecture with CODEX multiplexed imaging. *Cell* **174**, 968–981 (2018).
14. Yuzeir, A. *et al.* IntestLine: a shiny-based application to map the rolled intestinal tissue onto a line. *Bioinformatics* **39**, btad140 (2023).
15. Ni, Z. *et al.* SpotClean adjusts for spot swapping in spatial transcriptomics data. *Nat. Commun.* **13**, 2971 (2022).
16. 10x Genomics. Your introduction to Visium HD: Spatial biology in high definition. (2024).
17. Cable, D. M. *et al.* Robust decomposition of cell type mixtures in spatial transcriptomics. *Nat. Biotechnol.* **40**, 517–526 (2022).

18. Kleshchevnikov, V. *et al.* Cell2location maps fine-grained cell types in spatial transcriptomics. *Nat. Biotechnol.* **40**, 661–671 (2022).

19. 10x Genomics. Visium Spatial Gene Expression.

20. Rodriques, S. G. *et al.* Slide-seq: A scalable technology for measuring genome-wide expression at high spatial resolution. *Science* **363**, 1463–1467 (2019).

21. Palla, G. *et al.* Squidpy: a scalable framework for spatial omics analysis. *Nat. Methods* **19**, 171–178 (2022).

22. Larsson, L., Franzén, L., Ståhl, P. L. & Lundeberg, J. Semla: a versatile toolkit for spatially resolved transcriptomics analysis and visualization. *Bioinformatics* **39**, btad626 (2023).

REVIEWER COMMENTS

Reviewer #1 (Remarks to the Author):

The authors have very comprehensively addressed all my previous questions including the key benchmarking comparisons requested. I have no further comments or suggestions. The work represents an important and useful addition to spatial tissue analysis methods.

Thank you for the positive response and your thoughtful comments on the original manuscript.

Reviewer #2 (Remarks to the Author):

My concerns were all addressed.

Thank you for the positive response and your thoughtful comments on the original manuscript.

Reviewer #3 (Remarks to the Author):

Most of my comments have been addressed in this revision, but one comment remains unresolved.

"SMURF produced spatial domains that more closely matched known hippocampal anatomy than the other methods (Supplementary Fig.S3)." This is a comparison of SMURF with other methods for spatial domain detection. While the visual results suggest that SMURF outperforms other approaches, the argument would be significantly strengthened by including a quantitative measure of how closely the inferred domains match the reference anatomy.

In spatial transcriptomics methods that perform clustering at spot resolution, such "closeness" is typically evaluated using metrics like the adjusted Rand index (ARI). Although the qualitative comparison is compelling, reporting these scores for each method would make the results more rigorous and convincing. Moreover, providing quantitative benchmarks would be useful for future studies on single-cell transcript assignment, as it would help identify areas for further improvement.

SMURF addresses a different problem compared to methods such as GraphST or Spatial-MGCN, where clustering is the primary objective. Consequently, benchmark datasets used for GraphST and Spatial-MGCN typically include clustering-specific ground truth annotations that can serve as references for evaluation using metrics such as the adjusted Rand index (ARI). In contrast, numerical evaluation of SMURF may be more challenging due to the lack of clustering-specific ground truth annotations in datasets relevant to its task. If quantitative evaluation is not feasible, the authors should clearly explain why. Otherwise, the inclusion of appropriate quantitative metrics is strongly encouraged.

Thank you for this excellent suggestion. We calculated the Adjusted Rand Index (ARI) using the mouse hippocampus to benchmark the agreement between ST clusters and anatomic reference. As the reviewer points out, the Visium HD 10x hippocampus dataset we are using does not have a “ground truth” reference domain assignment. Thus, we defined the hippocampal regions using histology and gene markers, and used them as the reference input for ARI calculation with the sklearn package. Using this reference, SMURF achieved the highest ARI among all five methods, consistent with the qualitative observation that its inferred domains more closely matched known hippocampal substructures.

We modified Supplementary Note 1 (p. 7, l. 128-134) and Supplemental Figure S3 to reflect this analysis and to provide quantitative benchmarks for future studies. That text and the modified figure are pasted here for the reviewer’s convenience.

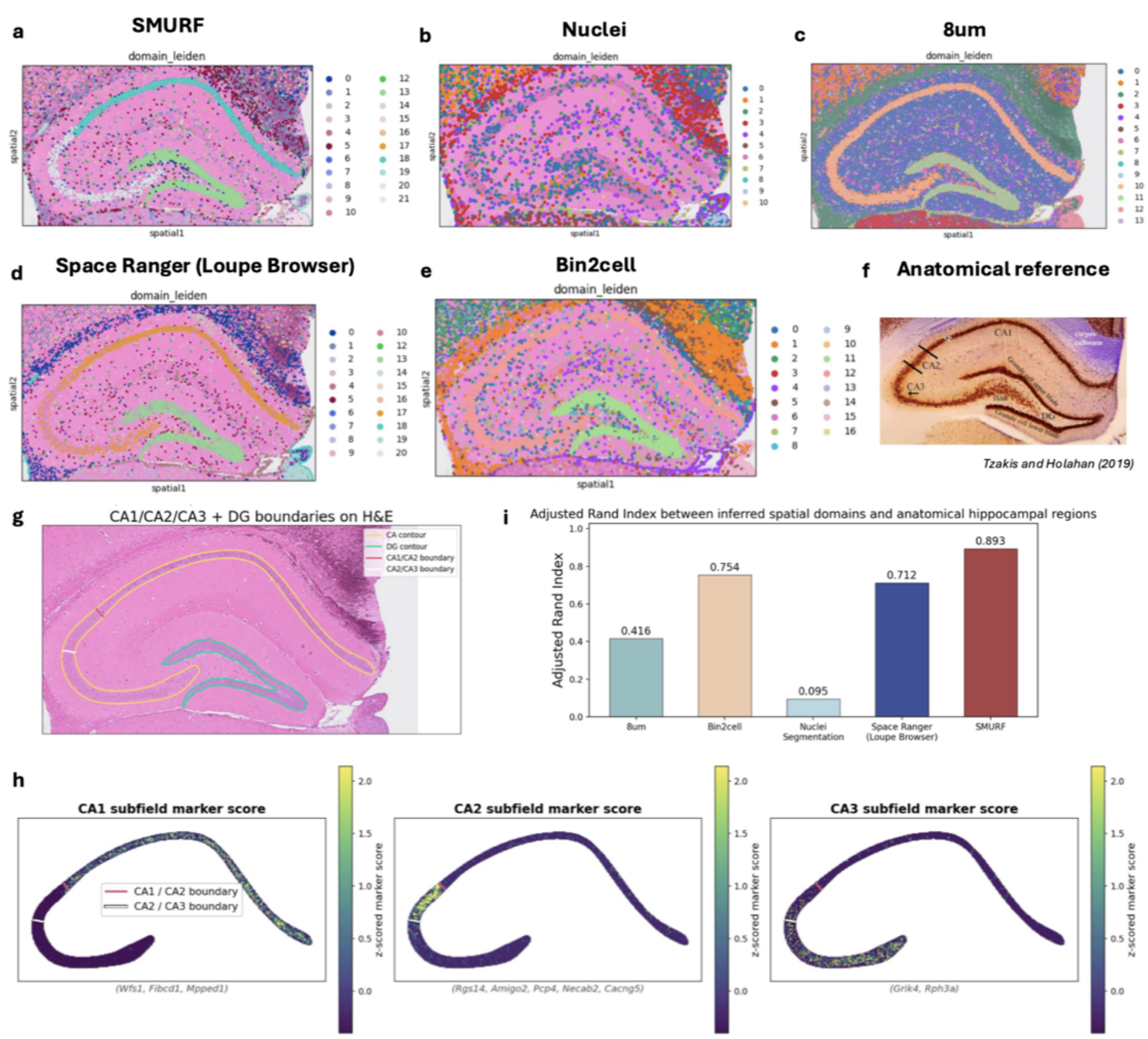

Fig. S3: **Comparison of downstream spatial domain identification across segmentation methods in the mouse brain.** Spatial Leiden domains in the hippocampal region are shown for (a) SMURF, (b) nuclei-only segmentation, (c) 8 μm binning, (d) Space Ranger (Loupe Browser), and (e) Bin2Cell, all analyzed using the same Squidpy-based downstream workflow. (f) Annotated anatomical reference image of the dorsal hippocampus, adapted from Tzakis and Holahan et al. [12]. (g) H&E image with CA and DG contours and inferred CA1/CA2 and CA2/CA3 boundaries overlaid. (h) CA1, CA2, and CA3 marker-score maps used to define the anatomy-informed hippocampal reference. (i) Adjusted Rand index (ARI) between inferred spatial domains and anatomical hippocampal regions, showing the highest agreement for SMURF.

“We used the Adjusted Rand Index (ARI) to quantify the agreement between hippocampal domains determined based on histology and the ST cluster assignments [13, 14]. We manually annotated 4 hippocampal subfields (CA1, CA2, CA3, and DG) based on the histology and canonical marker-gene patterns (Supplementary Fig. S3g,h). Using this reference, SMURF achieved the highest ARI among all five methods (Supplementary Fig. S3i), consistent with the qualitative observation that its inferred domains more closely matched known hippocampal substructures.”

Reviewer #4 (Remarks to the Author):

The other reviewers have given very insightful and helpful comments, and the authors have thoroughly and thoughtfully responded to these comments. Specifically, the benchmarks comparing SMURF to other relevant methods for assigning bins to cells and for unrolling the intestine Swiss roll convincingly show that SMURF is a valuable contribution in this area. However, the t-test comparing SMURF with deep learning and SMURF-lite does not seem very meaningful, because the effect size is so small despite the very significant p-values.

Thank you for your positive feedback. The reviewer raises an excellent point that we should emphasize. Deep learning does indeed provide a statistical advantage over the SMURF-lite method which doesn’t use deep learning. However, as the reviewer points out, the effect size is small. We have expanded the discussion about this topic, and to emphasize that these results establish SMURF-lite as a near equivalent tool for those without GPUs. We have added additional text to Supplementary Note 1 (p. 11, l. 204-211).

“A relatively small fraction of total transcripts is impacted because these methods only differ in how they handle spots shared by cells of the same cell type, so it is not surprising that the absolute difference between SMURF and SMURF-lite is modest. We recommend full SMURF for users with access to GPUs to achieve the highest performance. For those users without access to GPUs, it is reassuring that the SMURF-lite approach is a low-compute option that still provides best-in-class segmentation (Supplementary Fig. S4 and S5) along with advanced unrolling strategies to enhance biological interpretation.”
